# Updated analysis of the prescription and evaluation of protein kinase inhibitors for oncology in Germany

**DOI:** 10.1007/s00210-024-03377-0

**Published:** 2024-08-23

**Authors:** Caecilia S. Obst, Roland Seifert

**Affiliations:** Institute of Pharmacology, Hannover Medical School, Carl-Neuberg-Str. 1, 30625 Hannover, Germany

**Keywords:** Oncology, Targeted therapeutics, Early benefit assessment, Pharmaceutical costs, Health economics, AVR, Orphan drugs

## Abstract

A recent analysis from our group (https://pubmed.ncbi.nlm.nih.gov/37014400/) has shown that the immense costs of the 20 protein kinase inhibitors for oncology approved from 2015 to 2019 are largely caused by drugs whose additional benefit has not been proven. We updated our analysis by adding the newly approved protein kinase inhibitors of the years 2020 and 2021. Based on the 2021 and 2022 *Arzneiverordnungsreport* (AVR), we expanded our analysis to include a total of nine protein kinase inhibitors newly approved by the *European Medicines Agency* (EMA) in 2020 and 2021. As a result, 29 protein kinase inhibitors were identified for an update of our analysis. For these 29 drugs, all additional benefit assessments published by the *Gemeinsamer Bundesausschuss* (GBA) were analyzed. The additional benefit assessments of the GBA were compared with the corresponding assessments of the *European Society for Medical Oncology* (ESMO), the *Deutsche Gesellschaft für Hämatologie und Onkologie* (DGHO, *German Society for Hematology and Oncology*) and the *Arzneimittelkommission der deutschen Ärzteschaft* (AkdÄ, *Drug Commission of the German Medical Association*). In addition, a total number of 91 drug advertisements published in the journal *Oncology Research and Treatment* in 2022 were analyzed. The number of protein kinase inhibitors for which no additional benefit can be found by the GBA is increasing, whereas the number of drugs for which a considerable additional benefit can be found is decreasing. Thus, in the current 2022 (re)assessment of additional benefit by the GBA, no additional benefit was identified for 50% of the drugs (2020, 46%). Nineteen percent were assessed with a minor additional benefit (2020, 18%) and also 19% with a considerable additional benefit (2020, 27%). For 12% of the drugs, the additional benefit could not be quantified by the GBA (2020, 9%). The benefit assessments by other medical societies often differ significantly from those of the GBA, mainly due to different evaluations of various endpoint parameters. In addition, more and more protein kinase inhibitors are being approved as orphan drugs. However, their additional benefit cannot be quantified by the GBA in most cases (78%). In 38% of the advertisements of an oncology journal, protein kinase inhibitors are promoted, which shows the pharmacoeconomic importance of these drugs. In summary, the current additional benefit assessment procedure in Germany is very questionable, and reforms are urgently needed to maintain the stability of the German healthcare system, which is being undermined by the high cost of medicines, particularly for drugs whose additional benefits have not been proven.

**Supplementary Information:**

The online version contains supplementary material available at 10.1007/s00210-024-03377-0.

## Introduction

A recent analysis by our group showed that the immense costs of 20 protein kinase inhibitors for oncology, approved between 2015 and 2019 in Germany, were mainly caused by drugs for which no additional benefit could be found by the GBA (Obst and Seifert 2023). In view of the pharmacoeconomic importance of oncology drugs, in particular protein kinase inhibitors, the financial stability of our healthcare system is increasingly undermined by drugs for which no additional benefit can be found by the GBA. Based on the results of our initial publication, we updated our analysis by adding the newly approved protein kinase inhibitors of the years 2020 and 2021.

## Materials and methods

In our previous work, all new protein kinase inhibitors, approved by the *European Medicines Agency* (EMA) in the years 2015 to 2019 for oncological indications, were analyzed, based on the *Arzneiverordnungsreport* (AVR, *Drug Prescription Report*) for the years 2016 to 2020 (Schwabe and Paffrath 2016) (Paffrath et al. 2017) (Schwabe et al. 2018) (Schwabe et al. 2019) (Schwabe and Ludwig 2020). We expanded this analysis, based on the AVR 2021 and 2022, to include all other protein kinase inhibitors newly approved in 2020 and 2021 for oncological indications (Ludwig et al. 2021) (Ludwig et al. 2023a). As in our initial work, only newly approved drugs were considered whereas already approved protein kinase inhibitors with new indications or in new combinations were not. In addition, the drugs were classified according to the official anatomical-therapeutic-chemical classification with daily doses for Germany in 2023 (Bundesinstitut für Arzneimittel und Medizinprodukte (BfArM) 2023). According to this, only drugs that are classified under L01E as protein kinase inhibitors were examined. Taking these criteria into account, a total of 29 protein kinase inhibitors were identified for an update of our analysis. Six of these drugs (midostaurin, gilteritinib, avapritinib, fedratinib, pemigatinib and selumetinib) were approved as orphan drugs, i.e. drugs that are used for the treatment of orphan diseases. An overview of the analyzed drugs is shown in Table 1. Table S4 in the Supplement shows the pharmacological characterization of the analyzed drugs (Table S4).

**Table 1:** Overview of analyzed drugs and indications

	Drug (trading name)	Launch, indication
1a	Ceritinib (Zykadia®)	2015: ALK-positive, advanced NSCLC, previously treated with crizotinib
1b	Ceritinib (Zykadia®)	2017: ALK-positive, advanced NSCLC, first-line treatment
2	Cobimetinib (Cotellic®)	2015: metastatic melanoma with BRAF-V600-mutation
3a	Lenvatinib (Lenvima®)	2015: metastatic thyroid carcinoma
3b	Lenvatinib (Kisplyx®)	2016: advanced renal cell carcinoma
3c	Lenvatinib (Lenvima®)	2018: advanced or unresectable hepatocellular carcinoma
3d	Lenvatinib (Lenvima®)	2021: endometrial carcinoma, previously treated with platinum-containing therapy, in combination with pembrolizumab
3e	Lenvatinib (Kisplyx®)	2021: advanced renal cell carcinoma, first-line treatment, in combination with pembrolizumab
4	Nintedanib (Vargatef®)	2015: metastatic NSCLC
5a	Trametinib (Mekinist®)	2015: melanoma with BRAF-V600-mutation, in combination with dabrafenib
5b	Trametinib (Mekinist®)	2018: melanoma with BRAF-V600-mutation, in combination with dabrafenib, adjuvant therapy
5c	Trametinib (Mekinist®)	2017: advanced NSCLC with BRAF-V600-mutation, in combination with dabrafenib
6a	Osimertinib (Tagrisso®)	2016: metastatic NSCLC with T790M-EGFR-mutation
6b	Osimertinib (Tagrisso®)	2019: metastatic NSCLC with T790M-EGFR-mutation, first-line treatment
6c	Osimertinib (Tagrisso®)	2021: metastatic NSCLC with T790M-EGFR-mutation, adjuvant therapy, after complete tumour resection, pat. suitable for adjuvant platinum-based chemotherapy
6d	Osimertinib (Tagrisso®)	2021: metastatic NSCLC with T790M-EGFR-mutation, adjuvant therapy, after complete tumour resection, pat. not suitable for adjuvant platinum-based chemotherapy
7	Palbociclib (Ibrance®)	2016: hormone receptor-positive, HER2-negative, locally advanced or metastatic breast cancer
8a	Alectinib (Alecensa®)	2017: ALK-positive, advanced NSCLC, previously treated with crizotinib
8b	Alectinib (Alecensa®)	2017: ALK-positive, advanced NSCLC, first-line treatment
9a	Midostaurin (Rydapt®)	2017: AML with FLT3-mutation, *orphan drug*
9b	Midostaurin (Rydapt®)	2017: aggressive systemic mastocytosis, systemic mastocytosis with associated haematological neoplasm or mast cell leukaemia, *orphan drug*
10a	Ribociclib (Kisqali®)	2017: hormone receptor-positive, HER2-negative, locally advanced or metastatic breast cancer, in combination with an aromatase inhibitor
10b	Ribociclib (Kisqali®)	2017: hormone receptor-positive, HER2-negative, locally advanced or metastatic breast cancer, in combination with fulvestrant
11	Tivozanib (Fotivda®)	2017: advanced renal cell carcinoma, first-line treatment
12a	Abemaciclib (Verzenios®)	2018: hormone receptor-positive, HER2-negative, locally advanced or metastatic breast cancer, in combination with an aromatase inhibitor
12b	Abemaciclib (Verzenios®)	2018: hormone receptor-positive, HER2-negative, locally advanced or metastatic breast cancer, in combination with fulvestrant
12c	Abemaciclib (Verzenios®)	2018: hormone receptor-positive, HER2-negative, locally advanced or metastatic breast cancer, in combination with fulvestrant, postmenopausal pat., previously treated with endocrine therapy
12d	Abemaciclib (Verzenios®)	2022: hormone receptor-positive, HER2-negative, node-positive breast cancer, combination with endocrine therapy, early stage with high risk of recurrence, premenopausal pat.
12e	Abemaciclib (Verzenios®)	2022: hormone receptor-positive, HER2-negative, node-positive breast cancer, combination with endocrine therapy, early stage with high risk of recurrence, postmenopausal pat. and men
13	Binimetinib (Mektovi®)	2018: melanoma with BRAF-V600-mutation, in combination with encorafenib
14a	Encorafenib (Braftovi®)	2018: melanoma with BRAF-V600-mutation, in combination with binimetinib
14b	Encorafenib (Braftovi®)	2020: metastatic colorectal cancer with BRAF-V600-mutation after prior systemic therapy, in combination with cetuximab
15a	Brigatinib (Alunbrig®)	2019: ALK-positive, advanced NSCLC, previously treated with crizotinib
15b	Brigatinib (Alunbrig®)	2020: ALK-positive, advanced NSCLC, previously not treated with an ALK inhibitor, with brain metastases
15c	Brigatinib (Alunbrig®)	2020: ALK-positive, advanced NSCLC, previously not treated with an ALK inhibitor, without brain metastases
16	Dacomitinib (Vizimpro®)	2019: NSCLC with activating EGFR-mutations, first-line treatment
17	Gilteritinib (Xospata®)	2019: AML with FLT3-mutation, *orphan drug*
18	Larotrectinib (Vitrakvi®)	2019: tumours that display a Neurotrophic Tyrosine Receptor Kinase gene fusion
19a	Lorlatinib (Lorviqua®)	2019: ALK-positive, advanced NSCLC, previously treated with an ALK inhibitor
19b	Lorlatinib (Lorviqua®)	2022: ALK-positive, advanced NSCLC, previously not treated with an ALK inhibitor
20	Neratinib (Nerlynx®)	2019: hormone receptor-positive, HER2-overexpressed/amplified breast cancer
21a	Acalabrutinib (Calquence®)	2020: CLL, no 17p-deletion or TP53-mutation, pat. is suitable for FCR-therapy, monotherapy, first-line treatment
21b	Acalabrutinib (Calquence®)	2020: CLL, no 17p-deletion or TP53-mutation, pat. is not suitable for FCR-therapy, monotherapy, first-line treatment
21c	Acalabrutinib (Calquence®)	2020: CLL, with 17p-deletion or TP53-mutation, monotherapy, first-line treatment
21d	Acalabrutinib (Calquence®)	2020: CLL, no 17p-deletion or TP53-mutation, pat. is suitable for FCR-therapy, combination with obinutuzumab, first-line treatment
21e	Acalabrutinib (Calquence®)	2020: CLL, no 17p-deletion or TP53-mutation, pat. is not suitable for FCR-therapy, combination with obinutuzumab, first-line treatment
21f	Acalabrutinib (Calquence®)	2020: CLL, with 17p-deletion or TP53-mutation, combination with obinutuzumab, first-line treatment
21 g	Acalabrutinib (Calquence®)	2020: CLL, no 17p-deletion or TP53-mutation, after one previous therapy
21 h	Acalabrutinib (Calquence®)	2020: CLL, with 17p-deletion or TP53-mutation, after one previous therapy
21i	Acalabrutinib (Calquence®)	2020: CLL, after at least two previous therapies, idelalisib in combination with rituximab or rituximab in combination with bendamustine is patient-specific appropriate therapy
21j	Acalabrutinib (Calquence®)	2020: CLL, after at least two previous therapies, other therapy than idelalisib in combination with rituximab or rituximab in combination with bendamustine is patient-specific appropriate therapy
22a	Alpelisib (Piqray®)	2020: hormone receptor-positive, HER2-negative, locally advanced or metastatic breast cancer with PIK3CA-mutation, following endocrine therapy in the (neo-) adjuvant therapy situation, postmenopausal patients without liver or lung metastases
22b	Alpelisib (Piqray®)	2020: hormone receptor-positive, HER2-negative, locally advanced or metastatic breast cancer with PIK3CA-mutation, following endocrine therapy in the (neo-) adjuvant therapy situation, postmenopausal patients with liver or lung metastases
22c	Alpelisib (Piqray®)	2020: hormone receptor-positive, HER2-negative, locally advanced or metastatic breast cancer with PIK3CA-mutation, postmenopausal pat., following endocrine therapy in locally advanced or metastatic stage
22.d	Alpelisib (Piqray®)	2020: hormone receptor-positive, HER2-negative, locally advanced or metastatic breast cancer with PIK3CA-mutation, men
23a	Avapritinib (Ayvakyt®)	2020: unresectable or metastatic GIST with platelet-derived growth factor receptor alpha D842V mutation, *orphan drug*
23b	Avapritinib (Ayvakyt®)	2022: aggressive systemic mastocytosis, systemic mastocytosis with associated haematological neoplasm or mast cell leukaemia, after at least one previous systemic therapy, *orphan drug*
24a	Entrectinib (Rozlytrek®)	2020: tumours that display a Neurotrophic Tyrosine Receptor Kinase gene fusion
24b	Entrectinib (Rozlytrek®)	2020: ROS1-positve, advanced NSCLC
25	Fedratinib (Inrebic®)	2021: myelofibrosis, *orphan drug*
26	Pemigatinib (Pemazyre®)	2021: locally advanced or metastatic cholangiocarcinoma with FGFR2 fusion, *orphan drug*
27a	Selpercatinib (Retsevmo®)	2021: advanced NSCLC with RET-mutation
27b	Selpercatinib (Retsevmo®)	2021: advanced thyroid cancer with RET-mutation, previous treated with cabozantinib and/or vandetanib
27c	Selpercatinib (Retsevmo®)	2021: advanced thyroid cancer with RET-mutation, previous treated with sorafenib and/or lenvatinib
27d	Selpercatinib (Retsevmo®)	2022: advanced NSCLC with RET-mutation, first-line treatment
27e	Selpercatinib (Retsevmo®)	2023: advanced thyroid cancer, with RET-mutation, monotherapy
28	Selumetinib (Koselugo®)	2021: symptomatic, inoperable plexiform neurofibromas in paediatric patients with neurofibromatosis type 1, *orphan drug*
29	Tucatinib (Tukysa®)	2021: HER2-positive locally advanced or metastatic breast cancer in combination with trastuzumab and capecitabin

### Prescription data

For those 29 drugs, the number of prescriptions, sales, the defined daily dose (DDD) and the DDD costs were determined based on data from the *Wissenschaftliches Institut der Ortskrankenkassen* (WIdO, *Scientific Institute of the General Local Health Insurance Fund, AOK*) (https://www.wido.de, last accessed April 5, 2024). In each case, the values from the year of approval of the drug were compared with the values in 2022. For the drugs that were already analyzed in the initial work, the values for 2020 were also compared. The data refer to drugs prescribed by physicians for outpatient use and dispensed via public pharmacies at the expense of the GKV system. Due to changes in classification or DDD, there may be deviations from the AVR data. Table S1 in the Supplement provides an overview of the prescription data determined (Table S1).

### Additional benefit assessment

Furthermore, the additional benefit assessment was determined by the *Gemeinsamer Bundesausschuss* (GBA, *Federal Joint Committee)* for each drug (https://www.g-ba.de, last accessed April 5, 2024). Compared to the appropriate comparative (standard) therapy (ZVT), the GBA differentiated between six categories for the additional benefit assessment of a drug: major additional benefit, considerable additional benefit, minor additional benefit, not-quantifiable additional benefit, no additional benefit and less benefit (Gemeinsamer Bundesausschuss 2024). The initial additional benefit assessment of each drug for the indication of the marketing authorization was compared with all further additional benefit assessments for the respective drug published by the GBA until March 30, 2023. Thus, re-evaluations after the deadline of the initial evaluation, as well as additional benefit evaluations for new indications for the individual 29 drugs that resulted after the approval, were determined. For the drugs that were already part of the initial analysis, the additional benefit assessments were updated if new assessments were available. As a result, a total of 77 additional benefit assessments by the GBA could be identified for the 29 drugs in 68 indications. An overview of the analyzed additional benefit assessments is shown in Table S2 in the Supplement (Table S2).

### Further additional benefit assessments

Finally, the additional benefit assessments by the GBA were compared with the drug assessments by the *European Society for Medical Oncology* (ESMO), the *Arzneimittelkommission der deutschen Ärzteschaft* (AkdÄ, *Drug Commission of the German Medical Association*) and the *Deutsche Gesellschaft für Hämatologie und Onkologie* (DGHO, *German Society for Hematology and Oncology*), and similarities and differences were analyzed (https://www.esmo.org/guidelines/esmo-mcbs/esmo-mcbs-scorecards;https://www.akdae.de/stellungnahmen/amnog-fruehe-nutzenbewertung-nach-35a-sgb-v/wirkstoffe-a-z. and https://www.dgho.de/publikationen/stellungnahmen/fruehe-nutzenbewertung., last accessed April 5, 2024). An overview of the analyzed additional benefit assessments is shown in Table S3 in the Supplement (Table S3).

### Advertisements

In addition, the advertisements published in the oncological journal *Oncology Research and Treatment* were analyzed exemplarily (https://karger.com/ORT, last accessed April 5, 2024). For this purpose, ten issues of the journal from 2022 were examined with regard to published advertisements on drugs.

## Results

### Development of protein kinase inhibitor approvals

**Fig. 1:**
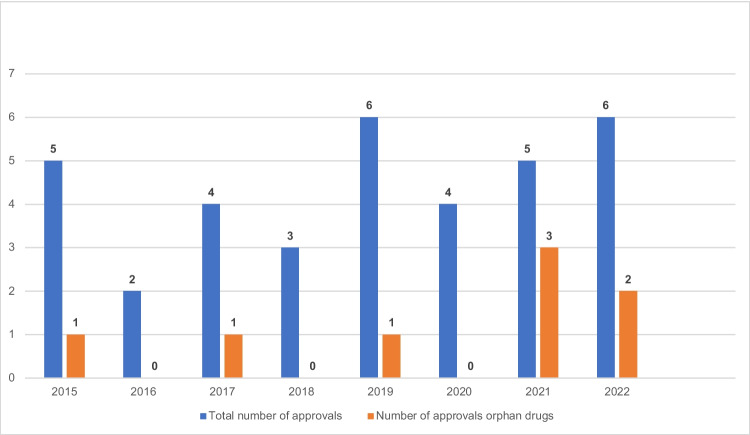
Development of protein kinase inhibitor approvals 2015–2022

Between 2015 and 2022, 35 protein kinase inhibitors were newly approved for oncological indications. A total of eight drugs were approved as orphan drugs (23%) (Fig. 1).

### Prescriptions

**Fig. 2:**
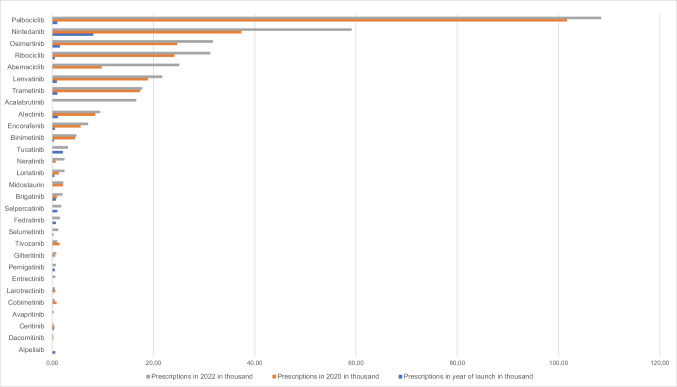
Development of prescriptions in thousand

Comparing the year of introduction and 2022, the number of prescriptions increased for each of the 29 drugs, except for ceritinib and alpelisib (Fig. 2). As in the initial analysis, palbociclib had the largest number of prescriptions in 2022 with 108.4 thousand prescriptions (compared to 2020, + 6.7%), followed by nintedanib with 59.1 thousand prescriptions (compared to 2020, + 58.1%), osimertinib with 31.7 thousand prescriptions (compared to 2020, + 28.7%) and ribociclib with 31.2 thousand prescriptions (compared to 2020, + 29.4%). The drugs dacomitinib, ceritinib, cobimetinib, larotrectinib and tivozanib were prescribed less frequently in 2022 than in 2020. For all other drugs, the number of prescriptions increased in 2022 compared to 2020.

### Sales

**Fig. 3:**
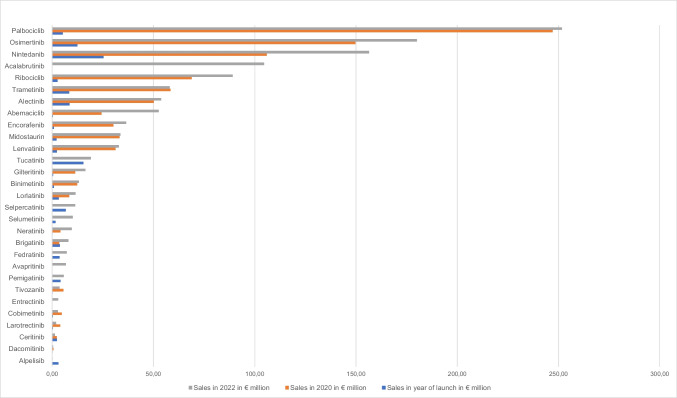
Development of sales in € million

Sales increased for 27 of the 29 drugs when comparing the year of introduction and 2022 (Fig. 3). It decreased for ceritinib and alpelisib. The drugs palbociclib (€251.66 million; compared to 2020, + 1.8%), osimertinib (€180.08 million; compared to 2020, + 20.3%) and nintedanib (€156.45 million; compared to 2020, + 47.6%) achieved the highest sales in 2022, followed by acalabrutinib (€104.67 million), which was approved in 2020. Compared to the results of our initial analysis, acalabrutinib replaced ribociclib, which follows in fifth place with 89.12 million euros (compared to 2020, + 29.5%). In 2022 dacomitinib, ceritinib, larotrectinib, cobimetinib, tivozanib and trametinib achieved lower sales than in 2020. For all other drugs, sales increased in 2022 compared to 2020.

### DDD

DDDs increased for 27 of the 29 drugs when comparing the year of introduction and 2022. They decreased for ceritinib and alpelisib. However, palbociclib (2550.90 thousand; compared to 2020, + 3.8%), nintedanib (1541.60 thousand; compared to 2020, + 55.6%), ribociclib (979.60 thousand; compared to 2020, + 31.8%) and osimertinib (858.90 thousand; compared to 2020, + 27.1%) had the most DDDs in 2022. These results are similar to those of our initial analysis.

### DDD costs

DDD costs are calculated by dividing net costs by DDD. For most drugs, both net costs and DDD increased; thus, DDD costs decreased. Exceptions were the drugs ceritinib, acalabrutinib, alpelisib, avapritinib and entrectinib. For ceritinib and alpelisib, net costs and DDD decreased; therefore, DDD costs increased. For acalabrutinib, avapritinib and entrectinib, no data on net costs and DDD were available for the year of approval, so we chose the value zero for the calculation, resulting in a mathematical increase in net costs.

### General development

The drugs palbociclib, osimertinib, nintedanib, acalabrutinib and ribociclib accounted for the highest sales in 2022, whereas the drugs palbociclib, nintedanib, osimertinib and ribociclib had the largest share of prescriptions and DDDs. Acalabrutinib followed in eighth place. For this reason, the drugs palbociclib, nintedanib, osimertinib and ribociclib will be referred as the *Top 4* in the following. These results are identical to those of our initial analysis. Palbociclib and ribociclib are CDK inhibitors used for the treatment of hormone receptor-positive, HER2-negative locally advanced or metastatic breast cancer. The angiokinase inhibitor nintedanib is used for the treatment of metastatic NSCLC, and EGFR inhibitor osimertinib is used for the treatment of metastatic NSCLC with T790M-EGFR-mutation. The four drugs alpelisib, dacomitinib, ceritinib and avapritinib had the lowest share of prescriptions and DDD in this analysis and are therefore referred to the *Flop 4* below. This result is different from that of our initial analysis, whereas the drugs alpelisib and avapritinib were approved in 2020 and were therefore not part of our initial analysis.

### Development of GBA additional benefit assessments in general

Compared with our initial analysis, in the update, a considerable additional benefit was found by the GBA for fewer drugs (27% vs. 19% (value initial analysis vs. value updated analysis)). The proportion of drugs that were assessed as having a minor additional benefit remained the same (18% vs. 19%), whereas the proportion of drugs for which no additional benefit could be found increased (46% vs. 50%). The proportion of drugs whose additional benefit could not be quantified by the GBA also increased (9% vs. 12%) (Table 2).

**Table 2:** GBA additional benefit assessments in the initial analysis and the updated analysis

GBA assessment	Initial analysis	Updated analysis
Considerable additional benefit	27%	19%
Minor additional benefit	18%	19%
No additional benefit	46%	50%
Not-quantifiable additional benefit	9%	12%

### Development of GBA additional benefit assessments—Top 4

Also within the group of the *Top 4* drugs, a considerable additional benefit was found by the GBA for less drugs in the update than in the initial analysis (29% vs. 25%). The proportion of drugs that were assessed with a minor additional benefit also decreased (43% vs. 37%), whereas the proportion of drugs for which no additional benefit could be found by the GBA increased (14% vs. 25%). The proportion of drugs whose additional benefit could not be quantified by the GBA remained the same (14% vs. 13%) (Table 3).

**Table 3:** GBA additional benefit assessments in the initial analysis and the updated analysis—Top 4

GBA assessment Top 4	Initial analysis	Updated analysis
Considerable additional benefit	29%	25%
Minor additional benefit	43%	37%
No additional benefit	14%	25%
Not-quantifiable additional benefit	14%	13%

### Development of GBA additional benefit assessments—Flop 4

Within the group of *Flop 4* drugs, the proportion of drugs for which a considerable additional benefit was found by the GBA also decreased (37% vs. 11%). A minor additional benefit was found more frequently in the updated analysis (13% vs. 22%), whereas the proportion of drugs without an additional benefit decreased (50% vs. 45%). In contrast, the proportion of drugs whose additional benefit could not be quantified by the GBA increased (0% vs. 22%) (Table 4).

**Table 4:** GBA additional benefit assessments in the initial analysis and the updated analysis—Flop 4

GBA assessment Flop 4	Initial analysis	Updated analysis
Considerable additional benefit	37%	11%
Minor additional benefit	13%	22%
No additional benefit	50%	45%
Not-quantifiable additional benefit	0%	22%

### GBA additional benefit assessments for orphan drugs

**Fig. 4:**
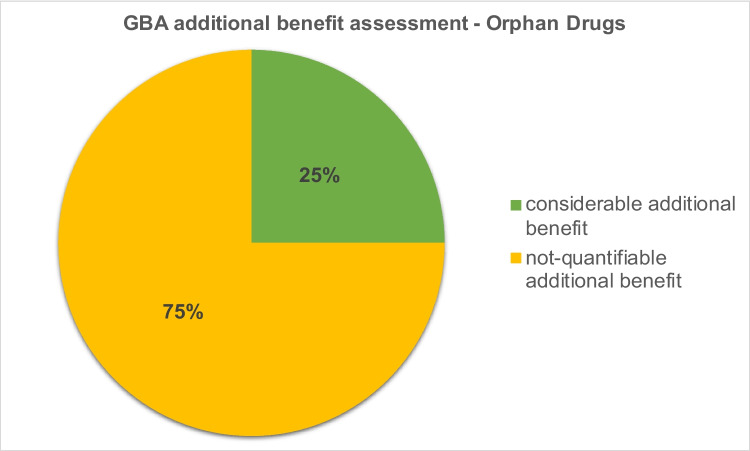
GBA additional benefit assessment of protein kinase inhibitors approved as orphan drugs 2015–2022

Compared to our initial analysis, the total number of indications for which protein kinase inhibitors were approved as orphan drugs increased (see the section “Development of protein kinase inhibitor approvals”). Our initial work included three protein kinase inhibitors in three indications. In the update, we were able to include six drugs in eight indications. In 75%, the additional benefit could not be quantified by the GBA. Twenty-five percent of the drugs were assessed with a considerable additional benefit (Fig. 4). In our initial analysis, no additional benefit could be found for 33% of the orphan drugs. Sixty-seven percent were assessed as having a considerable additional benefit.

### Evaluation by the ESMO

**Fig. 5:**
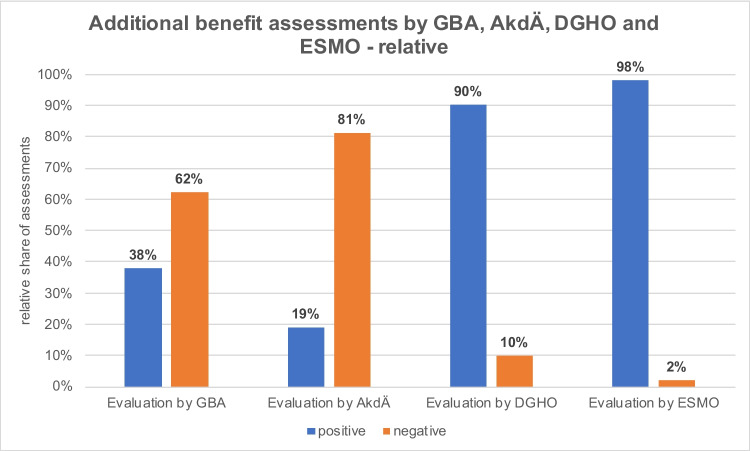
Additional benefit assessments by GBA, AkdÄ, DGHO and ESMO

ESMO evaluates drugs according to the *ESMO Magnitude of Clinical Benefit Scale* (ESMO-MCBS). The score distinguishes ratings for the palliative and curative settings. In the palliative setting, scores of 1 to 5 can be achieved, with scores of 5 and 4 rated as substantial benefit. In the curative setting, a score of A to C is assigned. Here, scores of A and B are considered substantial benefit. ESMO explicitly states that a high ESMO-MCBS score does not automatically imply a high clinical value of a drug but rather serves as an initial assessment of a drug which must be followed by further investigations in order to use available resources wisely and responsibly (https://www.esmo.org/guidelines/esmo-mcbs/about-the-esmo-mcbs, last accessed April 17, 2024). Based on this score, an evaluation was available for 23 of the 29 drugs in 50 indications. An overview of the analyzed additional benefit assessments is shown in Table S3 in the Supplement (Table S3). The ESMO assessed the additional benefit as positive for 98% of the analyzed drugs. For 2%, the assessment of additional benefit was negative (Fig. 5). In 72% of the additional benefit assessments, the ESMO evaluated better than the GBA (initial analysis, 67%). Twenty-four percent of the additional benefit assessments were the same (initial analysis, 33%), and in 4%, the ESMO assessed the additional benefit worse than the GBA (initial analysis, 0%).

### Evaluation by the DGHO

The DGHO does not perform a categorical/quantitative evaluation but expresses its opinion on the benefit of a drug in a differentiated written statement. An evaluation was available for 28 of the 29 drugs in 67 indications. An overview of the analyzed additional benefit assessments is shown in Table S3 in the Supplement (Table S3). In 90% of indications, the DGHO assessed positively. Negative additional benefit assessments were found in 10% (Fig. 5). In 54% of the assessments, the result of the DGHO was better than the one of the GBA (initial analysis, 43%). Forty-three percent of the assessments were the same (initial analysis, 57%), and in 3%, the DGHO evaluated worse than the GBA (initial analysis, 0%).

### Evaluation by the AkdÄ

The AkdÄ assesses the additional benefit of a drug in a written statement. This involves a quantitative assessment according to the six assessment categories of the GBA. An overview of the analyzed additional benefit assessments is shown in Table S3 in the Supplement (Table S3). An assessment by the AkdÄ was available for 12 of the 29 drugs in 16 indications. The additional benefit assessment was positive in 19% and negative in 81% of the evaluations (Fig. 5). In 6% of the assessments, the result of the AkdÄ was better than the one of the GBA. In 69%, the assessments were the same, and in 25%, the AkdÄ evaluated worse than the GBA. As our initial analysis did not include the assessments of the AkdÄ, no data can be compared at this point.

### Advertisements

**Fig. 6:**
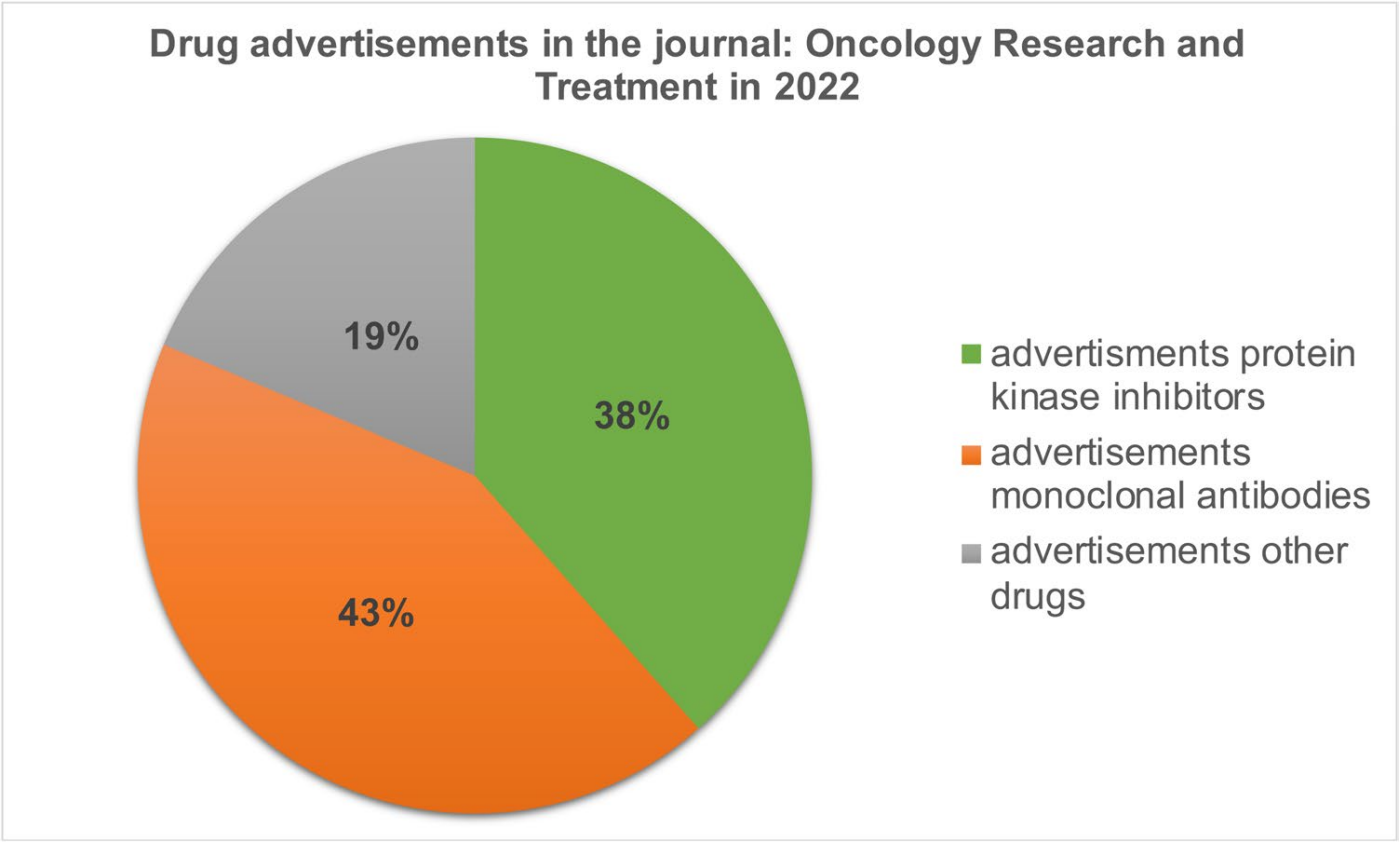
Relative share of drug groups in advertisements

In total, we analyzed 91 advertisements published in ten issues of the journal *Oncology Research and Treatment* in 2022. Thirty-eight percent of the advertisements promoted protein kinase inhibitors (2020, 39%), 43% monoclonal antibodies (2020, 32%) and 19% other drugs (2020, 29%) (Fig. 6). Twenty advertisements promoted 6 of the 29 drugs considered in this analysis. Twenty percent of the 20 advertisements promoted drugs from the *Top 4* group (2020, 23%). Drugs from the *Flop 4* group as well as orphan drugs were not advertised at all (2020: Flop 5, 44%; orphan drugs, 36%).

## Discussion

### Development of protein kinase inhibitor approvals

Oncology drugs have accounted for the largest share of newly approved drugs in recent years. In particular, many monoclonal antibodies and protein kinase inhibitors have been newly approved (Ludwig et al. 2023b). This development reflects the increasing implementation of the concept of “targeted therapy” in oncology. The trend in approvals of protein kinase inhibitors for oncological indications shows that also a significant proportion of these newly approved drugs are approved as orphan drugs.

### Prescriptions, sales and DDD

The increasing importance of protein kinase inhibitors is also reflected in the growing number of prescriptions, rising sales and increasing DDDs. In most cases, exceptions are due to the approval of second-generation drugs. For example, prescriptions of the ALK inhibitor ceritinib for the treatment of ALK-positive, advanced NSCLC decreased, but the ALK inhibitors lorlatinib and brigatinib, which were approved in 2019, and the ALK inhibitor alectinib, which was approved in 2017, are additional drugs for the treatment of NSCLC. The number of prescriptions and sales of these second-generation drugs increased accordingly. The development of second-generation drugs plays a role especially in the context of the development of resistance to the initial tumour therapy and thus represents an alternative therapeutic opportunity. Nevertheless, the high sales of protein kinase inhibitors also seem to create a pharmacoeconomic incentive for the development of “mee-too” drugs (Aronson and Green 2020). Furthermore, the additional benefit of second-generation drugs is often not better than that of first-generation drugs, as in the case of ALK inhibitors (Obst and Seifert 2023). The decrease in prescriptions, sales and DDD of the TRK inhibitor larotrectinib and the MEK inhibitor cobimetinib can also be explained by the corresponding approvals of second-generation drugs. Prescriptions and sales of the drug alpelisib also decreased. This development is discussed below (see the section “The case of alpelisib”).

In contrast to the exceptions mentioned above, especially the group of the *Top 4* showed significant increases in prescriptions, sales and DDD for 2022 compared to 2020, although often no additional benefit can be found by the GBA (see the section “Development of additional benefit assessments-Top 4 drugs”). Particularly noteworthy in this context is the drug nintedanib, whose sales increased by almost 50% in 2022 compared to 2020 and whose prescriptions increased by almost 60%. Here, it should be noted that the majority of prescriptions and sales were not achieved by the drug Vargatef® with an oncological indication (NSCLC), but rather by the drug Ofev®, which is approved for the treatment of idiopathic pulmonary fibrosis, as well as other indications. We had already discussed this in more detail in our initial analysis (Obst and Seifert 2023).

The BTK inhibitor acalabrutinib for the treatment of CLL achieved the fourth-highest sale in 2022 with the eighth-highest share of prescriptions and DDDs. No additional benefit was found by the GBA for six of the ten approved indications. In three indications, a minor additional benefit was found, and in one indication, a considerable additional benefit was found. Overall, the huge discrepancy between sales, number of prescriptions and additional benefit assessment becomes very obvious here.

### Development of additional benefit assessments

Our update shows that the number of newly approved protein kinase inhibitors for which no or a not-quantifiable additional benefit can be found by the GBA is increasing, whereas the number of drugs for which a considerable additional benefit can be found is decreasing. Often, no additional benefit is found for the respective drug because no relevant data are available, but not because the additional benefit is objectively worse than that of the ZVT (Flintrop 2024a). Therefore, it is important that pharmaceutical companies perform more studies with better quality. However, the final assessment is made more difficult by the fact that there is enormous heterogeneity between different institutions regarding which parameters are considered patient-relevant and are therefore included in the additional benefit assessment. Furthermore, drugs are increasingly being approved for secondary indications (e.g. other tumour entities). The probability that a drug has a high additional benefit in subsequent indications is significantly lower than in the initial indication (Vokinger et al. 2023).

### Development of additional benefit assessments—Top 4 drugs

Even within the group of the *Top 4*, the proportion of drugs for which no or a non-quantifiable additional benefit could be found by the GBA is increasing, whereas the proportion of drugs that were assessed with a minor or considerable additional benefit is decreasing. Although the assessments of the *Top 4* drugs are better compared to the total number of drugs assessed and compared to the *Flop 4* drugs, the increasing sales and the rising number of prescriptions must be questioned regarding the lack of additional benefit.

### Development of additional benefit assessments—Flop 4 drugs

Also within the group of *Flop 4* drugs, the additional benefit assessments are becoming increasingly worse. Compared to the total number of drugs assessed, the *Flop 4* drugs were assessed more negatively, although this appears acceptable in terms of their low share of prescriptions and sales.

### Development of additional benefit assessments—orphan drugs

Orphan drugs are drugs that are approved for the treatment of rare diseases. These are diseases that affect ≤ 5 in 10,000 people in the EU (Europäisches Parlament 2000). Once a drug has been approved as an orphan drug by the EMA, its additional benefit is assumed to be proven. Only when a sales threshold of €30 million is exceeded, the additional benefit must be quantified in a regular assessment procedure by the GBA. The main aim of orphan drug designations is to create incentives for companies to develop drugs for rare diseases that are associated with high investment costs and low profits due to the small patient population.

Our analysis shows that more and more protein kinase inhibitors are being approved as orphan drugs. This “orphanization” can be explained by the increasingly differentiated application approvals. As a result, a smaller patient population is addressed in a common tumour entity, and the definition of a rare disease is reached more frequently. Regarding accelerated and simplified approval procedures and other pharmacoeconomic incentives, the approval of drugs as orphan drugs appears to be gaining in importance, whereby the original intention of orphan drug designation seems to become less important.

During their development, many of the drugs approved as orphan drugs reach the sales threshold of €30 million and must pass GBA’s regular additional benefit assessment procedure: here, often no additional benefit can be found, even 10 years after the initial approval of the drug. Also, in our analysis, it can be seen that the additional benefit of protein kinase inhibitors approved as orphan drugs remains not-quantifiable in most cases, whereas the proportion of orphan drugs for which a considerable additional benefit was found is decreasing. Due to the discrepancy between the additional benefit and pharmacoeconomic success of orphan drugs, it is being increasingly demanded that drugs approved as orphan drugs should also initially have to pass through the GBA’s regular additional benefit assessment procedure. In addition, the definition of orphan drug status needs to be revised in the context of the concept of “targeted therapy” in oncology (Schwabe et al. 2019) (Osterloh 2022) (Schenk 2023) (Flintrop 2024b). It should be noted that our sample is limited to six analyzed drugs in eight indications. Nevertheless, trends can be derived.

### Further additional benefit assessments

#### ESMO

The assessment by the ESMO was positive in almost all cases. The GBA and ESMO are increasingly coming to different conclusions, whereby the ESMO assessment is usually better than the one of the GBA. It should be noted that no ESMO-MCBS assessments for haematological neoplasms are available so far (as of April 30, 2024). Accordingly, our analysis only includes assessments for solid tumours.

#### DGHO

The assessment of the DGHO was also positive in most cases, although the GBA and DGHO increasingly come to different conclusions, which are usually better for the DGHO than for the GBA. Overall, the differences in the assessment results between the GBA and DGHO are less significant than between the GBA and ESMO. These differences are consistent with the results of our initial analysis and may be due to different assessment criteria at the national and international levels. It should be noted that the comparability of the DGHO and GBA assessments is limited due to the lack of a quantitative assessment by the DGHO. Nevertheless, trends can be derived.

#### AkdÄ

In contrast to the ESMO and DGHO, the AkdÄ assesses negatively in most cases. The AkdÄ often comes to the same conclusion as the GBA, or the assessment is worse. Overall, the assessments of the AkdÄ and GBA can be compared very well, as the AkdÄ’s quantitative assessment is based on the GBA’s assessment criteria.

#### In general

Overall, the comparison of the additional benefit assessments by the different professional societies shows very heterogeneous results. Especially the additional benefit assessments by the GBA and the AkdÄ differ significantly from those of the DGHO and the ESMO. This raises the question of whether possible conflicts of interest influence the results of the additional benefit assessments. Possible conflicts of interest of participants in the GBA additional benefit assessment procedure must be disclosed in accordance with the rules of the GBA in the form of a special document bevor the procedure starts (Gemeinsamer Bundesausschuss 2024). The same applies to those involved in the DGHO and AkdÄ assessment procedure (https://www.dgho.de/d-g-h-o/downloads/interessenkonflikte/offenlegung-interessenskonflikte-2018.pdf, last accessed 14 July 2024) (Arzneimittelkommission der deutschen Ärzteschaft 2014). The ESMO assessment procedure is carried out within the framework of the ESMO-MCBS in the form of a structured and validated scoring system that does not allow any conflicts of interest (European Society for Medical Oncology 2024). Furthermore, the ESMO also critically addressed the disclosure and handling of potential conflicts of interest in the form of a “Declaration of Interest Policy” (European Society for Medical Oncology 2020). Overall, a partial conflict of interest of the clinical societies could not be excluded completely, but we see the varying assessment of different endpoint parameters as the main reason for the diverging results regarding the extent of the additional benefit assessment. Particularly regarding the endpoint progression-free survival (PFS), there are different opinions within and between the professional societies regarding the patient relevance of that endpoint (Gemeinsamer Bundesausschuss 2024) (European Society for Medical Oncology 2024) (Arzneimittelkommission der deutschen Ärzteschaft 2020). The differences are increasing over the years, resulting in significant challenges for clinical oncologists when making treatment decisions. Especially regarding to the planned uniform additional benefit assessment for oncological drugs at the EU level from 2025, standardized assessment criteria must be created quickly. In this context, only the assessment of the clinical benefit will be evaluated at the EU level. Price negotiations should continue to remain at the national level. The EU assessment should primarily include clinical and patient-relevant endpoints. A transparent, evidence-based and precise methodology must be developed, for which existing and validated concepts such as the ESMO-MCBS could be used (Hwang and Vokinger 2022) (Wörmann 2024). In view of the current heterogeneity in the evaluation criteria, this project appears challenging.

### The case of alpelisib

The PI3K inhibitor alpelisib was approved in 2020 for the treatment of hormone receptor-positive, HER2-negative, locally advanced, or metastatic breast cancer with a PIK3CA mutation. On May 1, 2021, the drug was withdrawn from the market by the company (Ludwig et al. 2021). The reason for this was the GBA’s early additional benefit assessment, which found no or at best a minor additional benefit in the different indications. In accordance with the AMNOG procedure, the result of the additional benefit assessment would have led to discounts on the price. Lastly, the pharmaceutical company and the health insurance funds were unable to agree in the price negotiations, and the drug was withdrawn from the market. Medical societies, including the DGHO, criticized the GBA’s decision to not consider the endpoint PFS in their assessment. Alpelisib showed a prolongation of PFS, but not of overall survival (OS), compared to the ZVT, but the endpoint PFS was not considered patient-relevant by the GBA and was therefore not included in the additional benefit assessment. The professional societies also criticized the formation of subgroups by the GBA, which would further complicate the assessment in already small patient populations. Alpelisib is currently only available in Germany through import from other European countries (Deutsche Gesellschaft für Hämatologie und Medizinische Onkologie (DGHO), Deutsch Gesellschaft für Senologie (DGS), Deutsche Gesellschaft für Gynäkologie und Geburtshilfe (DGGG), Frauenselbsthilfe Krebs Bundesverband 2021). Even though the development of alpelisib is a specific case, it illustrates the problems and hurdles of the additional benefit assessment procedure and shows the relevant clinical consequences that the heterogeneity of the individual assessments and assessment criteria can have.

### Advertisements

The analysis of the advertisements shows similar results to our initial work, with a trend towards an increase in the proportion of advertisements for “targeted therapeutics”. Particularly drugs from the group of the *Top 4* were advertised, with a similar frequency as in the initial analysis. The update also showed no correlation between the proportion of advertisements and the clinical benefit of the respective drug. In contrast to the initial analysis, neither orphan drugs nor drugs from the *Flop 4* group were advertised in 2022. This illustrates the pharmacoeconomic importance of this group of drugs and reflects the pharmacoeconomic interest of pharmaceutical companies in the development and marketing of high-priced drugs. It should be noted that not all issues of the journal from 2022 could be analyzed. However, with a total of 91 advertisements analyzed, trends can be derived.

### General trends

Our analysis shows that the drug costs of oncologicals and especially targeted therapeutics are increasing rapidly. This will result in an overload of the healthcare system’s financial resources, and stable patient care can not longer be guaranteed.

Oncological therapies achieve greater social acceptance and willingness to pay higher drug prices, e.g. due to emotional aspects, which Serra-Burriel et al. very fittingly termed as the “cancer premium” (Serra-Burriel et al. 2023). In 2022, the entire oncology drugs group reached net costs of €10 629.10 million with 8.71 million prescriptions. The drug Biso Lich® with the active drug bisoprolol was prescribed a very similar number of times (8.07 million). However, with a similar prescription volume, Biso Lich® achieved more than 100 times lower net costs (€97.19 Mio) (Ludwig et al. 2023b). Bisoprolol is used for the treatment of chronic heart failure, as well as other indications. According to the German Heart Report from 2022, the age-standardized mortality rate for chronic heart failure in Germany in 2021 was 35.8% Deutsche Herzstiftung e.V. (2023). The comparison with the cardiological disease of chronic heart failure illustrates the enormous discrepancy in terms of treatment costs with similar mortality rates. However, the great heterogeneity of tumour entities and the corresponding treatment regimens must be considered. In addition, most high-priced oncology drugs are patent-protected drugs and not generics, such as bisoprolol. Moreover, the 8.71 million prescriptions for Biso Lich ® include not only the indication of heart failure therapy but also other indications, such as the treatment of arterial hypertension. Nevertheless, the general discrepancy and the special status of oncological drugs become apparent. This special status of oncology drugs must be questioned, particularly in view of the frequent lack of correlation between the price of the drug and the respective additional benefit.

**Fig. 7:**
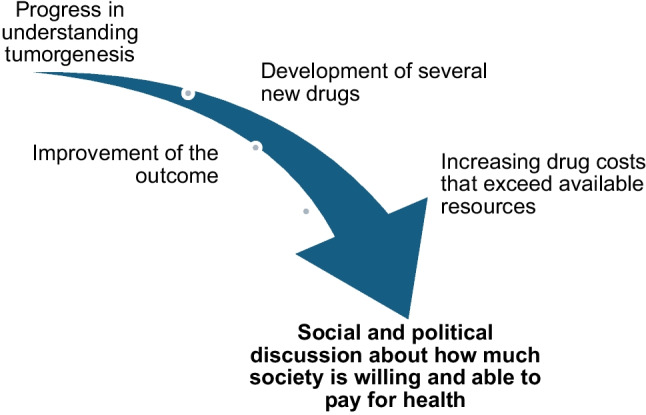
Development of modern oncology

In view of rising pharmaceutical expenditure with limited resources, a socio-political discussion is needed about how much society is willing and able to pay for healthcare. This includes not only the decision for a therapy but also for a therapy limitation. Protein kinase inhibitors are often seen by patients as the last straw of hope, which they do not want to miss. Although the adverse drug reactions (ADR) of protein kinase inhibitors differ considerably from those of classic cytostatics, the ADRs of this group of drugs should not be underestimated and must be critically weighed against the clinical benefits of the drugs (Gharwan and Groninger 2016) (Lee et al. 2018). Figure 7 shows the development of modern oncology and the resulting challenges and opportunities.

## Limitations of this study

As in our initial analysis, the data in the update is based exclusively on publicly available information. Therefore, only apparent discrepancies can be identified. In addition, the prescription, sales and DDD data we analyzed only include drugs that were prescribed by outpatient physicians at the expense of the SHI. Drugs prescribed in hospitals or reimbursed by private health insurance were not included in our analysis. Further limitations can be found in the respective sections.

## Conclusions

Our work shows that the discrepancy between prescriptions and sales and the corresponding additional benefit of a drug is increasing. Of course, the high drug costs also reflect the enormous progress that has been made in the treatment of oncological diseases, particularly in recent years. Due to those innovations, the long-term survival of many patients has been improved. Nevertheless, innovation and progress cannot justify the current level of drug prices for oncology drugs (Lau 2023). In addition, the concept of “targeted therapy” in oncology with prolonged patient survival creates further challenges regarding the increasingly long duration of treatment, e.g. in terms of maintenance therapy. New concepts are therefore needed to ensure the long-term stability of the German healthcare system and guarantee safe and sufficient patient care at the same time. For example, German Cancer Aid will fund projects in the future that investigate the influence of economic aspects on treatment decisions (Richter-Kuhlmann 2022). Adjusting and revising the statutory framework conditions are also important points for stabilizing price trends. The effects of the Financial Stabilization Act, which was passed in October 2022, remain to be seen. Further measures such as the assessment of the additional benefit of oncology drugs at the EU level from next year or a revision of the definition of orphan drug status in the context of precision medicine in oncology may represent useful but also challenging concepts. Another concept are confidential reimbursement prices for new, patent-protected drugs, provided in the draft of the new Medical Research Act (MFG). The aim is to give pharmaceutical companies more flexibility in price negotiations, which should lead to price reductions. Critics fear an increase in bureaucracy and see the project in conflict with the principle of price transparency and the economic selection of a drug provided in SGB V. There are also fears that it would lead to further price increases rather than price reductions (Lau et al. 2024) (Lau and Beerheide 2024).

It remains to be seen which concepts and measures will become practicable and effective. However, urgent action is required. Furthermore, a socio-political discussion is needed about how much society is willing and able to pay for health. It should be noted that, compared to other European countries, new drugs are approved very quickly in Germany, also due to the lucrative pricing regulation. This can change due to strict regulations and have a negative impact on patient care (Schenk 2023). This balancing act must now be addressed effectively.

## Supplementary Information

Below is the link to the electronic supplementary material.

Supplementary file1 (DOCX 172 KB)

## Data Availability

All source data for this study are available upon reasonable request from the authors.

## Abbreviations

AkdÄ: Arzneimittelkommission der deutschen Ärzteschaft (Drug Commission of the German Medical Association)
ALK: Anaplastic lymphoma kinase
AVR: Arzneiverordnungsreport (Drug prescription report)
BTK: Bruton’s tyrosine kinase
CDK: Cyclin-dependent kinase
CLL: Chronic lymphatic leukaemia
DDD: Daily defined dose
DGHO: Deutsche Gesellschaft für Hämatologie und Onkologie (German Society for Hematology and Oncology)
EGFR: Epidermal growth factor receptor
EMA: European Medicines Agency
ESMO: European Society for Medical Oncology
GBA: Gemeinsamer Bundesausschuss (Federal Joint Committee)
GKV: Gesetzliche Krankenversicherung (Statutory health insurance, SHI)
HER2: Human epidermal growth factor receptor-2
MEK: Mitogen-activated protein kinase
MFG: Medizinforschungsgesetz (Medical Research Act)
NSCLC: Non-small cell lung cancer
OS: Overall survival
PFS: Progression-free survival
SGB: Sozialgesetzbuch (German Social Code)
TRK: Tropomyosin receptor kinase
WIdO: Wissenschaftliches Institut der Ortskrankenkassen (AOK) (Scientific Institute of the General Local Health Insurance Fund, AOK)
ZVT: Appropriate comparative (standard) therapy

## References

[CR1] Aronson JK, Green AR (2020) Me-too pharmaceutical products: history, definitions, examples, and relevance to drug shortages and essential medicines lists. Br J Clin Pharmacol. 10.1111/bcp.14327 32358800 10.1111/bcp.14327 PMC7576625

[CR2] Arzneimittelkommission der deutschen Ärzteschaft (2014) Regeln zum Umgang mit Interessenkonflikten bei Mitgliedern der Arzneimittelkommission der deutschen Ärzteschaft. https://www.akdae.de/fileadmin/user_upload/akdae/Kommission/Organisation/Statuten/Interessenkonflikte/Regeln.pdf. Accessed 15 July 2024

[CR3] Arzneimittelkommission der deutschen Ärzteschaft (2020) 10 Jahre frühe Nutzenbewertung nach AMNOG - Stellungsnahmen der AkdÄ. Arzneiverordnung in der Praxis 47, 3–4. https://www.akdae.de/fileadmin/user_upload/akdae/Arzneimitteltherapie/AVP/Artikel/2020-3-4/171.pdf. Accessed 05 May 2024

[CR4] Bundesinstitut für Arzneimittel und Medizinprodukte (BfArM) (2023) Anatomisch-therapeutisch-chemische Klassifikation mit Tagesdosen - Amtliche Fassung des ATC-Index mit DDD-Angaben für Deutschland im Jahre 2023

[CR5] Deutsche Gesellschaft für Hämatologie und Medizinische Onkologie (DGHO), Deutsch Gesellschaft für Senologie (DGS), Deutsche Gesellschaft für Gynäkologie und Geburtshilfe (DGGG), Frauenselbsthilfe Krebs Bundesverband (2021) Gemeinsame Pressemitteilung von DGHO Deutsche Gesellschaft für Hämatologie und Medizinische Onkologie e.V., Deutsche Gesellschaft für Senologie e.V. (DGS), Deutsche Gesellschaft für Gynäkologie und Geburtshilfe e.V. (DGGG) und Frauenselbsthilfe Krebs Bundesverband e.V.: Marktrücknahme von Alpelisib zulasten von Brustkrebspatient*innen. https://www.dgho.de/aktuelles/presse/pressemeldungen/marktruecknahme-von-alpelisib-zulasten-von-brustkrebspatient-innen. Accessed 05 May 2024

[CR6] Deutsche Herzstiftung e.V. (2023) Deutscher Herzbericht 2022. https://epaper.herzstiftung.de/#0. Acessed 15 July 2024

[CR7] Europäisches Parlament (2000) Verordnung (EG) Nr. 141/2000 des Europäischen Parlaments und des Rates vom 16. Dezember 1999 über Arzneimittel für seltene Leiden. https://eur-lex.europa.eu/LexUriServ/LexUriServ.do?uri=OJ:L:2000:018:0001:0005:DE:PDF. Accessed 05 May 2024

[CR8] European Society for Medical Oncology (ESMO) (2020) Declaration of Interest (DOI) Policy. https://www.esmo.org/content/download/408443/7889903/1/ESMO-DOI-Policy.pdf. Accessed 15 July 2024

[CR9] European Society for Medical Oncology (ESMO) (2024) ESMO-MCBS Factsheet. https://www.esmo.org/content/download/288505/5736229/1/ESMO-MCBS-Factsheet.pdf. Accessed 05 May 2024

[CR10] Flintrop J (2024a) Neu heißt nicht immer besser. Deutsches Ärzteblatt *121*, 3. https://www.aerzteblatt.de/pdf.asp?id=237355. Accessed 05 May 2024

[CR11] Flintrop J (2024b) Arzneimittel gegen seltene Leiden: Die Spreu vom Weizen trennen. Deutsches Ärzteblatt *121*, 9. https://www.aerzteblatt.de/pdf.asp?id=238757. Accessed 05 May 2024

[CR12] Gemeinsamer Bundesausschuss (GBA) (2024) Verfahrensordnung des Gemeinsamen Bundesausschusses. https://www.g-ba.de/downloads/62-492-3375/VerfO_2023-10-19_iK_2024-02-20.pdf. Accessed 05 May 2024

[CR13] Gharwan H, Groninger H (2016) Kinase inhibitors and monoclonal antibodies in oncology: clinical implications. Nat Rev Clin Oncol. 10.1038/nrclinonc.2015.213 26718105 10.1038/nrclinonc.2015.213

[CR14] Hwang TJ, Vokinger KN (2022) New EU regulation on health technology assessment of cancer medicines. Lancet Oncol. 10.1016/S1470-2045(22)00008-0 35114127 10.1016/S1470-2045(22)00008-0

[CR15] Lau T, Beerheide R (2024) Vertrauliche Erstattungspreise: Warnung vor Bürokratiemonster. Deutsches Ärzteblatt. https://www.aerzteblatt.de/nachrichten/150357/Vertrauliche-Erstattungspreise-Warnung-vor-Buerokratiemonster. Accessed 05 May 2024

[CR16] Lau T, Ludwig W, Joachimsen K, Tebinka-Olrich A (2024) Wie senkt man Preise?. Deutsches Ärzteblatt *121*, 4. https://www.aerzteblatt.de/pdf.asp?id=237727. Accessed 05 May 2024

[CR17] Lau T (2023) Onkologikapreisen steht kein entsprechender therapeutischer Nutzen gegenüber. Deutsches Ärzteblatt. https://www.aerzteblatt.de/nachrichten/144677/Onkologikapreisen-steht-kein-entsprechender-therapeutischer-Nutzen-gegenueber. Accessed 05 May 2024

[CR18] Lee YT, Tan YJ, Oon CE (2018) Molecular targeted therapy: treating cancer with specificity. Eur J Pharmacol. 10.1016/j.ejphar.2018.07.034 30031797 10.1016/j.ejphar.2018.07.034

[CR19] Ludwig W, Mühlbauer B, Seifert R (2021) Arzneiverordnungs-Report 2021. Springer, Berlin Heidelberg, Berlin, Heidelberg

[CR20] Ludwig W, Mühlbauer B, Seifert R (2023a) Arzneiverordnungs-Report 2022. Springer, Berlin / Heidelberg, Berlin, Heidelberg

[CR21] Ludwig W, Mühlbauer B, Seifert R (2023b) Arzneiverordnungs-Report 2023. Springer, Berlin, Heidelberg

[CR22] Obst CS, Seifert R (2023) Critical analysis of the prescription and evaluation of protein kinase inhibitors for oncology in Germany. Naunyn Schmiedebergs Arch Pharmacol. 10.1007/s00210-023-02475-9 37014400 10.1007/s00210-023-02475-9 PMC10497443

[CR23] Osterloh F (2022) Wenige Informationen über den Zusatznutzen. Deutsches Ärzteblatt *119*, 3. https://www.aerzteblatt.de/pdf.asp?id=222875. Accessed 05 May 2024

[CR24] Paffrath D, Ludwig W, Klauber J, Schwabe U (2017) Arzneiverordnungs-Report 2017: aktuelle Daten, Kosten, Trends und Kommentare. Springer, Berlin

[CR25] Richter-Kuhlmann E (2022) Onkologie Eng verknüpft mit ethischen Aspekten. Deutsches Ärzteblatt 119*,* 14. https://www.aerzteblatt.de/pdf.asp?id=224566. Accessed 05 May 2024

[CR26] Schenk M (2023) Kostenexplosion in der Onkologie. Deutsches Ärzteblatt 120, 37. https://www.aerzteblatt.de/pdf.asp?id=234036. Accessed 05 May 2024

[CR27] Schwabe U, Paffrath D (2016) Arzneiverordnungs-Report 2016: Aktuelle Daten, Kosten, Trends und Kommentare. Springer Berlin Heidelberg, Berlin, Heidelberg

[CR28] Schwabe U, Paffrath D, Ludwig W, Klauber J (2018) Arzneiverordnungs-Report 2018: aktuelle Daten, Kosten, Trends und Kommentare. Springer, Berlin

[CR29] Schwabe U, Paffrath D, Ludwig W, Klauber J (2019) Arzneiverordnungs-Report 2019: akutelle Daten, Kosten, Trends und Kommentare. Springer, Berlin

[CR30] Schwabe U, Ludwig W (2020) Arzneiverordnungs-Report 2020. Springer, Berlin Heidelberg, Berlin, Heidelberg

[CR31] Serra-Burriel M, Perényi G, Laube Y, Mitchell AP, Vokinger KN (2023) The cancer premium - explaining differences in prices for cancer vs non-cancer drugs with efficacy and epidemiological endpoints in the US, Germany, and Switzerland: a cross sectional study. EClinicalMedicine. 10.1016/j.eclinm.2023.102087 37521033 10.1016/j.eclinm.2023.102087 PMC10371812

[CR32] Vokinger KN, Glaus CEG, Kesselheim AS, Serra-Burriel M, Ross JS, Hwang TJ (2023) Therapeutic value of first versus supplemental indications of drugs in US and Europe (2011–20): retrospective cohort study. BMJ. 10.1136/bmj-2022-074166 37407074 10.1136/bmj-2022-074166 PMC10320829

[CR33] Wörmann B (2024) Verlagerung der Nutzenbewertung neuer Arzneimittel auf die EU-Ebene: Risiken und Chancen für Deutschland. DGHO Mitgliederrundschreiben 01/24

