# Supplementary material for: Updated analysis of the prescription and evaluation of protein kinase inhibitors for oncology in Germany
Source: Naunyn Schmiedebergs Arch Pharmacol. 2024 Aug 23;398(2):1799–813. doi: 10.1007/s00210-024-03377-0 (PMC11825581; doi:10.1007/s00210-024-03377-0)
Supplement: Supplementary file 1 — Supplementary file1 (DOCX 172 KB) [file 210_2024_3377_MOESM1_ESM.docx]

**Supplemental tables S1-S4**

**Caecilia Sophia Obst and Roland Seifert**

**Updated analysis of the prescription and evaluation of protein kinase inhibitors for oncology in Germany**

|  | **Drug, Launch (trading name)** | **Indication** | **Mechanism of Action** | **Prescriptions in year of launch in thousand** | **Prescriptions in 2022 in thousand** | **Change in %** | **Sales in year of launch in € million** | **Sales in 2022 in € million** | **Change in %** | **DDD in years of launch in thousand** | **DDD in 2022 in thousand** | **Change in %** | **DDD-costs in year of launch in €** | **DDD-costs in 2022 in €** | **Change in %** |
| --- | --- | --- | --- | --- | --- | --- | --- | --- | --- | --- | --- | --- | --- | --- | --- |
| **1.** | Ceritinib,  **2015** (Zykadia^®^) | ALK-positive, advanced NSCLC | ALK inhibitor | 0.30 | 0.20 | - 33.33 | 2.31 | 1.36 | - 41.13 | 13.60 | 7.40 | - 45.59 | 170.06 | 183.15 | + 7.70 |
| **2.** | Cobimetinib, **2015** (Cotellic^®^) | Metastatic melanoma with BRAF-V600-mutation | MEK inhibitor | 0.10 | 0.50 | + 400.00 | 0.41 | 2.79 | + 580.49 | 1.60 | 14.30 | + 793.75 | 241.00 | 195.22 | - 18.99 |
| **3.** | Lenvatinib, **2015** (Lenvima^®^) | Metastatic thyroid carcinoma | Multiple kinase inhibitor | 0.90 | 21.70 | + 2 311.11 | 2.34 | 32.92 | +  1 306.84 | 12.60 | 273.00 | +  2 066.67 | 186.33 | 120.55 | - 35.30 |
| **4.** | Nintedanib, **2015** (Vargatef^®^) | Metastatic NSCLC | Angiokinase inhibitor | 8.10 | 59.10 | + 629.63 | 25.35 | 156.45 | + 517.16 | 216.10 | 1541.60 | + 613.37 | 117.30 | 101.49 | - 13.48 |
| **5.** | Trametinib, **2015** (Mekinist^®^) | Melanoma with BRAF-V600-mutation | MEK inhibitor | 1.00 | 17.80 | +  1 680.00 | 8.38 | 57.95 | + 591.53 | 29.10 | 396.90 | +  1 263.92 | 288.07 | 146.01 | - 49.31 |
| **6.** | Osimertinib,  **2016** (Tagrisso^®^) | Metastatic NSCLC with T790M-EGFR-mutation | EGFR inhibitor | 1.50 | 31.70 | +  2 013.33 | 12.43 | 180.08 | +  1 348.75 | 44.90 | 858.90 | +  1 812.92 | 277.08 | 209.67 | - 24.33 |
| **7.** | Palbociclib, **2016** (Ibrance^®^) | Hormone receptor-positive, HER2-negative locally advanced or metastatic breast cancer | CDK inhibitor | 1.00 | 108.40 | +  10 740.00 | 5.23 | 251.66 | +  4 711.85 | 27.70 | 2550.90 | +  9 109.03 | 188.90 | 98.66 | - 47.77 |
| **8.**  **Table S1** Development of protein kinase inhibitors after launch - prescriptions, sales, DDD and DDD-costs | Alectinib, **2017** (Alecensa^®^) | ALK-positive, advanced NSCLC, previously treated with crizotinib | ALK inhibitor | 1.10 | 9.50 | + 763.63 | 8.59 | 53.79 | + 526.19 | 30.30 | 267.30 | + 782.18 | 283.72 | 201.23 | - 29.07 |
| **9.** | Midostaurin, **2017** (Rydapt^®^) | AML with FLT3-mutation; aggressive systemic mastocytosis, systemic mastocytosis with associated haematological neoplasm or mast cell leukaemia | Multiple kinase inhibitor | 0.10 | 2.20 | +  2 100.00 | 2.13 | 33.66 | +  1 480.28 | 2.90 | 62.50 | +  2 055.17 | 726.41 | 538.51 | - 25.87 |
| **10.** | Ribociclib, **2017** (Kisqali^®^) | Hormone receptor-positive, HER2-negative locally advanced or metastatic breast cancer | CDK inhibitor | 0.50 | 31.20 | +  6 140.00 | 2.68 | 89.12 | +  3 225.37 | 13.10 | 979.60 | +  7 377.86 | 205.34 | 90.98 | - 55.69 |
| **11.** | Tivozanib, **2017** (Fotivda^®^) | Advanced renal cell carcinoma, first-line treatment | VEGFR inhibitor | 0.03 | 1.00 | +  3 233.33 | 0.14 | 3.64 | +  2 500.00 | 0.80 | 23.20 | +  2 800.00 | 166.13 | 156.67 | - 5.69 |
| **12.** | Abemaciclib, **2018** (Verzenios^®^) | Hormone receptor-positive, HER2-negative locally advanced or metastatic breast cancer | CDK inhibitor | 0.10 | 25.10 | +  25 000.00 | 0.39 | 52.57 | +  13 379.49 | 2.90 | 630.30 | +  21 634.48 | 126.38 | 83.40 | - 34.01 |
| **13.** | Binimetinib, **2018** (Mektovi^®^) | Melanoma with BRAF-V600-mutation | MEK inhibitor | 0.30 | 4.80 | +  1 500.00 | 0.86 | 13.20 | +  1 434.88 | 3.60 | 127.80 | +  3 450.00 | 238.79 | 103.26 | - 56.76 |
| **14.** | Encorafenib, **2018** (Braftovi^®^) | Melanoma with BRAF-V600-mutation | BRAF inhibitor | 0.50 | 7.10 | +  1 320.00 | 0.81 | 36.60 | +  4 418.52 | 3.30 | 164.10 | +  4 872.73 | 244.95 | 223.05 | - 8.94 |
| **15.** | Brigatinib, **2019** (Alunbrig^®^) | ALK-positive, advanced NSCLC, previously treated with crizotinib | ALK inhibitor | 0.70 | 2.00 | + 185.71 | 3.82 | 7.98 | + 108.90 | 13.60 | 39.50 | + 190.44 | 280.55 | 202.14 | - 27.95 |
| **16.**  **Table S1** (continued) | Dacomitinib, **2019** (Vizimpro^®^) | NSCLC with EGFR-activation mutations, first-line treatment | EGFR inhibitor | 0.03 | 0.19 | + 533.33 | 0.13 | 0.44 | + 238.46 | 0.60 | 4.05 | + 575.00 | 202.83 | 108.60 | - 46.46 |
| **17.** | Gilteritinib, **2019** (Xospata^®^) | AML with FLT3-mutation | FLT3 inhibitor | 0.02 | 0.08 | + 300.00 | 0.43 | 16.35 | +  3 702.33 | 0.50 | 23.70 | +  4 640.00 | 805.60 | 688.74 | - 14.51 |
| **18.** | Larotrectinib, **2019** (Vitrakvi^®^) | Tumors that display a Neurotrophic Tyrosine Receptor Kinase gene fusion | TRK inhibitor | 0.10 | 0.50 | + 400.00 | 0.40 | 1.93 | + 382.50 | 0.60 | 10.50 | +  1 650.00 | 633.83 | 183.37 | - 71.07 |
| **19.** | Lorlatinib, **2019** (Lorviqua^®^) | ALK-positive, advanced NSCLC | ALK inhibitor | 0.40 | 2.40 | + 500.00 | 3.21 | 11.51 | + 258.57 | 12.40 | 67.70 | + 445.97 | 258.19 | 170.16 | - 34.10 |
| **20.** | Neratinib, **2019** (Nerlynx^®^) | Hormone receptor positive, HER2-overexpressed/ amplified breast cancer | HER2 inhibitor | 0.00 | 2.40 | + 240.00 | 0.03 | 9.64 | +  32 033.33 | 0.10 | 71.90 | +  71 800.00 | 246.00 | 134.09 | - 45.49 |
| **21.** | Acalabrutinib, **2020**  (Calquence^®^) | CLL | BTK inhibitor | 0.00 | 16.60 | +  1 660.00 | 0.00 | 104.67 | +  10 467.00 | 0.00 | 497.90 | +  49 790.00 | 0.00 | 210.20 | +  21 020.00 |
| **22.** | Alpelisib **2020**  (Piqray^®^) | Hormone receptor positive, HER2-negative locally advanced or metastatic breast cancer with PIK3CA mutation | Pi3K inhibitor | 0.60 | 0.03 | - 95.00 | 3.06 | 0.17 | - 94.44 | 13.90 | 0.74 | - 94.68 | 220.62 | 234.50 | + 6.29 |
| **23.** | Avapritinib **2020**  (Ayvakyt^®^)  **Table S1** (continued) | Unresectable or metastatic GIST with platelet-derived growth factor receptor alpha D842V mutation; aggressive systemic mastocytosis, systemic mastocytosis with associated haematological neoplasm or mast cell leukaemia | KIT  inhibitor | 0.00 | 0.30 | + 30.00 | 0.00 | 6.72 | + 672.00 | 0.00 | 4.90 | + 490.00 | 0.00 | 1371.81 | + 137 181.00 |
| **24.** | Entrectinib **2020**  (Rozlytrec^®^) | Tumors that display a Neurotrophic Tyrosine Receptor Kinase gene fusion; ROS1-positive advanced NSCLC | TRK inhibitor | 0.00 | 0.60 | + 60.00 | 0.00 | 2.94 | + 294.00 | 0.00 | 16.50 | +  1 650.00 | 0.00 | 178.14 | +  17 814.00 |
| **25.** | Fedratinib **2021**  (Inrebic^®^) | Myelofibrosis | JAK inhibitor | 0.70 | 1.50 | + 114.29 | 3.63 | 7.16 | + 97.25 | 20.40 | 46.40 | + 127.45 | 178.10 | 154.38 | - 13.32 |
| **26.** | Pemigatinib **2021**  (Pemazyre^®^) | Locally advanced or metastatic cholangiocarcinoma with FGFR2 fusion | FGFR inhibitor | 0.50 | 0.70 | + 40.00 | 4.12 | 5.74 | + 39.32 | 8.00 | 12.10 | + 51.25 | 516.18 | 473.32 | - 8.30 |
| **27.** | Selpercatinib **2021**  (Retsevmo^®^) | Advanced RET fusion-positive NSCLC; advanced RET fusion-positive thyroid cancer | RET inhibitor | 1.00 | 1.80 | + 80.00 | 6.65 | 11.30 | + 69.92 | 14.30 | 37.00 | + 158.74 | 464.25 | 305.06 | - 34.29 |
| **28.** | Selumetinib **2021**  (Koselugo^®^) | Symptomatic, inoperable plexiform neurofibromas in paediatric patients with neurofibromatosis type 1 | MEK inhibitor | 0.20 | 1.20 | + 500.00 | 1.65 | 10.17 | + 516.34 | 3.10 | 20.30 | + 554.84 | 523.22 | 501.23 | - 4.20 |
| **29.** | Tucatinib **2021**  (Tukysa^®^) | HER2-positive locally advanced or metastatic breast cancer | HER2 inhibitor | 2.10 | 3.10 | + 47.62 | 15.40 | 19.08 | + 23.90 | 42.70 | 60.20 | + 40.98 | 360.35 | 317.16 | - 11.99 |

**Table S1** (continued)

|  | **Drug (trading name)** | **Launch, Indication** | **First GBA benefit assessment** | **GBA reassessment** |
| --- | --- | --- | --- | --- |
| **1a.** | Ceritinib (Zykadia^®^) | **2015:** ALK-positive, advanced NSCLC, previously treated with crizotinib | **2015:** No additional benefit [1] | **2017:** Considerable additional benefit [2] |
| **1b.** | Ceritinib (Zykadia^®^) | **2017:** ALK-positive, advanced NSCLC, first-line treatment | **2018**: No additional benefit [3] |  |
| **2.** | Cobimetinib (Cotellic^®^) | **2015:** Metastatic melanoma with BRAF-V600-mutation | **2016:** Considerable additional benefit [4] |  |
| **3a.** | Lenvatinib (Lenvima^®^) | **2015:** Metastatic thyroid carcinoma | **2015:** Not-quantifiable additional benefit, orphan drug [5] | **2019:** No additional benefit [6] |
| **3b.** | Lenvatinib (Kisplyx^®^) | **2016:** Advanced renal cell carcinoma | **2017:** Minor additional benefit [7] | **2021:** No additional benefit [8] |
| **3c.** | Lenvatinib  (Lenvima^®^) | **2018:** Advanced or unresectable hepatocellular carcinoma | **2019:** No additional benefit [9] |  |
| **3d.**  **Table S2** Development of protein kinase inhibitors after launch - first GBA benefit assessment and reassessment | Lenvatinib  (Lenvima^®^) | **2021:** Endometrial carcinoma, previously treated with platinum-containing therapy, in combination with pembrolizumab | **2022:** Considerable additional benefit [10] |  |
| **3e.** | Lenvatinib (Kisplyx^®^) | **2021:** Advanced renal cell carcinoma, first-line treatment, in combination with pembrolizumab | **2022:** No additional benefit [11] |  |
| **4.** | Nintedanib  (Vargatef^®^) | **2015:** Metastatic NSCLC | **2015:** Minor additional benefit [12] |  |
| **5a.** | Trametinib (Mekinist^®^) | **2015:** Melanoma with BRAF-V600-mutation, in combination with dabrafenib | **2016:** Considerable additional benefit [13] |  |
| **5b.** | Trametinib (Mekinist^®^) | **2018:** Melanoma with BRAF-V600-mutation, in combination with dabrafenib, adjuvant therapy | **2019:** Considerable additional benefit [14] |  |
| **5c.** | Trametinib (Mekinist^®^) | **2017:**  Advanced NSCLC with BRAF-V600-mutation, in combination with dabrafenib | **2017:** No additional benefit [15] |  |
| **6a.**  **Table S2** (continued) | Osimertinib (Tagrisso^®^) | **2016:** Metastatic NSCLC with T790M-EGFR-mutation | **2016:** No additional benefit [16] | **2017:** Considerable additional benefit [17] |
| **6b.** | Osimertinib  (Tagrisso^®^) | **2019:** Metastatic NSCLC with T790M-EGFR-mutation, first-line treatment | **2019:** Considerable additional benefit [18] |  |
| **6c.** | Osimertinib (Tagrisso^®^) | **2021:** Metastatic NSCLC with T790M-EGFR-mutation, adjuvant therapy, after complete tumor resection, pat. suitable for adjuvant platinum-based chemotherapy | **2021:** No additional benefit [19] |  |
| **6d.** | Osimertinib (Tagrisso^®^) | **2021:** Metastatic NSCLC with T790M-EGFR-mutation, adjuvant therapy, after complete tumor resection, pat. not suitable for adjuvant platinum-based chemotherapy | **2021:** Not-quantifiable additional benefit [19] |  |
| **7.**  **Table S2** (continued) | Palbociclib (Ibrance^®^) | **2016:** Hormone receptor-positive, HER2-negative, locally advanced or metastatic breast cancer | **2017:** No additional benefit [20] | **2022:** No additional benefit [21], [22] |
| **8a.** | Alectinib (Alecensa^®^) | **2017:** ALK-positive, advanced NSCLC, previously treated with crizotinib | **2017:** Minor additional benefit [23] |  |
| **8b.** | Alectinib  (Alecensa^®^) | **2017:** ALK-positive, advanced NSCLC, first-line treatment | **2018:** Not-quantifiable additional benefit [24] |  |
| **9a.** | Midostaurin (Rydapt^®^) | **2017:** AML with FLT3-mutation | **2018:** Considerable additional benefit, orphan drug [25] |  |
| **9b.** | Midostaurin (Rydapt^®^) | **2017:** Aggressive systemic mastocytosis, systemic mastocytosis with associated haematological neoplasm or mast cell leukaemia | **2018:** Not-quantifiable additional benefit, orphan drug [26] |  |
| **10a.** | Ribociclib (Kisqali^®^) | **2017:** Hormone receptor-positive, HER2-negative, locally advanced or metastatic breast cancer, in combination with an aromatase inhibitor | **2018:** No additional benefit [27] | **2020:** Minor additional benefit [28] |
| **10b.**  **Table S2** (continued) | Ribociclib (Kisqali^®^) | **2017:** Hormone receptor-positive, HER2-negative, locally advanced or metastatic breast cancer, in combination with fulvestrant | **2019:** No additional benefit [29] | **2020:** Minor additional benefit [30] |
| **11.** | Tivozanib (Fotivda^®^) | **2017:** Advanced renal cell carcinoma, first-line treatment | **2018:** No additional benefit [31] |  |
| **12a.** | Abemaciclib  (Verzenios^®^) | **2018:** Hormone receptor-positive, HER2-negative, locally advanced or metastatic breast cancer, in combination with an aromatase inhibitor | **2019:** No additional benefit [32] | **As at March 28th 2023:** Start of process: 01.01.2023, decision-making estimated middle of June 2023 |
| **12b.** | Abemaciclib (Verzenios^®^) | **2018:** Hormone receptor-positive, HER2-negative, locally advanced or metastatic breast cancer, in combination with fulvestrant | **2019**: No additional benefit [33] | **2022:** No additional benefit [34] |
| **12c.**  **Table S2** (continued) | Abemaciclib (Verzenios^®^) | **2018:** Hormone receptor-positive, HER2-negative, locally advanced or metastatic breast cancer, in combination with Fulvestrant, postmenopausal pat., previously treated with endocrine therapy | **2019:** No additional benefit [33] | **2022:** Minor additional benefit [34] |
| **12d.** | Abemaciclib (Verzenios^®^) | **2022:** Hormone receptor-positive, HER2-negative, node-positive breast cancer, combination with endocrine therapy, early stage with high risk of recurrence, premenopausal pat. | **2022:** Minor additional benefit [35] |  |
| **12e.** | Abemaciclib (Verzenios^®^) | **2022:** Hormone receptor-positive, HER2-negative, node-positive breast cancer, combination with endocrine therapy, early stage with high risk of recurrence, postmenopausal pat. and men | **2022:** No additional benefit [35] |  |
| **13.** | Binimetinib (Mektovi^®^) | **2018:** Melanoma with BRAF-V600-mutation, in combination with encorafenib | **2019**: No additional benefit [36] |  |
| **14a.**  **Table S2** (continued) | Encorafenib (Braftovi^®^) | **2018:** Melanoma with BRAF-V600-mutation, in combination with binimetinib | **2019:** No additional benefit [37] |  |
| **14b.** | Encorafenib (Braftovi^®^) | **2020:** Metastatic colorectal cancer with BRAF-V600-mutation after prior systemic therapy, in combination with cetuximab | **2020:** Considerable additional benefit [38] |  |
| **15a.** | Brigatinib (Alunbrig^®^) | **2019:** ALK-positive, advanced NSCLC, previously treated with crizotinib | **2019:** No additional benefit [39] |  |
| **15b.** | Brigatinib (Alunbrig^®^) | **2020:** ALK-positive, advanced NSCLC, previously not treated with an ALK inhibitor, with brain metastases | **2020:** Considerable additional benefit [40] |  |
| **15c.** | Brigatinib (Alunbrig^®^) | **2020:** ALK-positive, advanced NSCLC, previously not treated with an ALK inhibitor, without brain metastases | **2020:** Minor additional benefit [40] |  |
| **16.**  **Table S2** (continued) | Dacomitinib (Vizimpro^®^) | **2019:** NSCLC with activating EGFR-mutations, first-line treatment | **2019:** No additional benefit [41] |  |
| **17.** | Gilteritinib (Xospata^®^) | **2019:** AML with FLT3-mutation | **2020:** Considerable additional benefit, orphan drug [42] |  |
| **18.** | Larotrectinib (Vitrakvi^®^) | **2019:** Tumors that display a Neurotrophic Tyrosine Receptor Kinase gene fusion | **2020:** No additional benefit [43] |  |
| **19a.** | Lorlatinib (Lorviqua^®^) | **2019:** ALK-positive, advanced NSCLC, previously treated with an ALK inhibitor | **2019:** No additional benefit [44] |  |
| **19b.** | Lorlatinib (Lorviqua^®^) | **2022:** ALK-positive, advanced NSCLC, previously not treated with an ALK inhibitor | **2022:** No additional benefit [45] |  |
| **20.** | Neratinib (Nerlynx^®^) | **2019:** Hormone receptor-positive, HER2- overexpressed/amplified breast cancer | **2020:** Minor additional benefit [46] |  |
| **21a.**  **Table S2** (continued) | Acalabrutinib (Calquence^®^) | **2020:** CLL, no 17p-deletion or TP53-mutation, pat. os suitable for FCR-therapy, monotherapy, first-line treatment | **2021:** No additional benefit [47] |  |
| **21b.** | Acalabrutinib (Calquence^®^) | **2020:** CLL, no 17p-deletion or TP53-mutation, pat. is not suitable for FCR-therapy, monotherapy, first-line treatment | **2021:** Minor additional benefit [47] |  |
| **21c.** | Acalabrutinib (Calquence^®^) | **2020:** CLL, with 17p-deletion or TP53-mutation, monotherapy, first-line treatment | **2021:** No additional benefit [47] |  |
| **21d.** | Acalabrutinib (Calquence^®^) | **2020:** CLL, no 17p-deletion or TP53-mutation, pat. is suitable for FCR-therapy, combination with obinutuzumab, first-line treatment | **2021:** No additional benefit [48] |  |
| **21e.**  **Table S2** (continued) | Acalabrutinib (Calquence^®^) | **2020:** CLL, no 17p-deletion or TP53-mutation, pat. is not suitable for FCR-therapy, combination with obinutuzumab, first-line treatment | **2021:** Minor additional benefit [48] |  |
| **21f.** | Acalabrutinib (Calquence^®^) | **2020:** CLL, with 17p-deletion or TP53-mutation, combination with obinutuzumab, first-line treatment | **2021:** No additional benefit [48] |  |
| **21g.** | Acalabrutinib (Calquence^®^) | **2020:** CLL, no 17p-deletion or TP53-mutation, after one previous therapy | **2021:** No additional benefit [49] |  |
| **21h.** | Acalabrutinib (Calquence^®^) | **2020:** CLL, with 17p-deletion or TP53-mutation, after one previous therapy | **2021:** Considerable additional benefit [49] |  |
| **21i.** | Acalabrutinib (Calquence^®^) | **2020:** CLL, after at least two previous therapies, idelalisib in combination with rituximab or rituximab in combination with bendamustine is patient-specific appropriate therapy | **2021:** Minor additional benefit [49] |  |
| **21j.**  **Table S2** (continued) | Acalabrutinib (Calquence^®^) | **2020:** CLL, after at least two previous therapies, other therapy than idelalisib in combination with rituximab or rituximab in combination with bendamustine is patient-specific appropriate therapy | **2021:** No additional benefit [49] |  |
| **22a.** | Alpelisib (Piqray^®^) | **2020:** Hormone receptor-positive, HER2-negative, locally advanced or metastatic breast cancer with PIK3CA-mutation, following endocrine therapy in the (neo-) adjuvant therapy situation, postmenopausal patients without liver or lung metastases | **2021:** Minor additional benefit [50] |  |
| **22b.**  **Table S2** (continued) | Alpelisib (Piqray^®^) | **2020:** Hormone receptor-positive, HER2-negative, locally advanced or metastatic breast cancer with PIK3CA-mutation, following endocrine therapy in the (neo-) adjuvant therapy situation, postmenopausal patients with liver or lung metastases | **2021:** No additional benefit [50] |  |
| **22c.** | Alpelisib (Piqray^®^) | **2020:** Hormone receptor-positive, HER2-negative, locally advanced or metastatic breast cancer with PIK3CA-mutation, postmenopausal pat., following endocrine therapy in locally advanced or metastatic stage | **2021:** Minor additional benefit [50] |  |
| **22.d** | Alpelisib (Piqray^®^) | **2020:** Hormone receptor-positive, HER2-negative, locally advanced or metastatic breast cancer with PIK3CA-mutation, men | **2021:** No additional benefit [50] |  |
| **23a.** | Avapritinib (Ayvakyt^®^) | **2020:** Unresectable or metastatic GIST with platelet-derived growth factor receptor alpha D842V mutation | **2021:** Not-quantifiable additional benefit, orphan drug [51] |  |
| **23b.**  **Table S2** (continued) | Avapritinib (Ayvakyt^®^) | **2022:** aggressive systemic mastocytosis, systemic mastocytosis with associated haematological neoplasm or mast cell leukaemia, after at least one previous systemic therapy | **2022:** Not-quantifiable additional benefit, orphan drug [52] |  |
| **24a.** | Entrectinib (Rozlytrec^®^) | **2020:** Tumors that display a Neurotrophic Tyrosine Receptor Kinase gene fusion | **2021:** No additional benefit [53] |  |
| **24b.** | Entrectinib (Rozlytrec^®^) | **2020:** ROS1-positve, advanced NSCLC | **2021:** No additional benefit [54] |  |
| **25.** | Fedratinib (Inrebic^®^) | **2021:** Myelofibrosis | **2021:** Not-quantifiable additional benefit, orphan drug [55] |  |
| **26.** | Pemigatinib (Pemazyre^®^) | **2021:** Locally advanced or metastatic cholangiocarcinoma with FGFR2 fusion | **2021:** Not-quantifiable additional benefit, orphan drug [56] |  |
| **27a.** | Selpercatinib (Retsevmo^®^) | **2021:** Advanced NSCLC with RET-mutation | **2021:** No additional benefit [57] |  |
| **27b.**  **Table S2** (continued) | Selpercatinib (Retsevmo^®^) | **2021:** Advanced thyroid cancer with RET-mutation, previous treated with cabozantinib and/or vandetanib | **2021:** No additional benefit [58] |  |
| **27c.** | Selpercatinib (Retsevmo^®^) | **2021:** Advanced thyroid cancer with RET-mutation, previous treated with sorafenib and/or Lenvatinib | **2021:** No additional benefit [59] |  |
| **27d.** | Selpercatinib (Retsevmo^®^) | **2022:** Advanced NSCLC with RET-mutation, first-line treatment | **2022:** No additional benefit [60] |  |
| **27e.** | Selpercatinib (Retsevmo^®^) | **2023:** Advanced thyroid cancer, with RET-mutation, monotherapy | **2023:** No additional benefit [61] |  |
| **28.** | Selumetinib (Koselugo^®^) | **2021:** Symptomatic, inoperable plexiform neurofibromas in paediatric patients with neurofibromatosis type 1 | **2022:** Not-quantifiable additional benefit, orphan drug [62] |  |
| **29.** | Tucatinib (Tukysa^®^) | **2021:** HER2-positive locally advanced or metastatic breast cancer in combination with trastuzumab and capecitabin | **2021:** Considerable additional benefit [63] |  |

**Table S2** (continued)

|  | **Drug (trading name)** | **Launch, Indication** | **GBA benefit assessment** | **Evaluation by ESMO** | **Evaluation by AkdÄ** | **Evaluation by DGHO** |
| --- | --- | --- | --- | --- | --- | --- |
| **1a.** | Ceritinib (Zykadia^®^) | **2015:** ALK-positive, advanced NSCLC, previously treated with crizotinib | **2017:** Considerable additional benefit [1] | **2023:** 4 [56] | **2017:** No additional benefit [98] | **2017:** Increase in remission rate, prolongation of PFS and improvement in clinical symptoms [111] |
| **1b.** | Ceritinib (Zykadia^®^) | **2017:** ALK-positive, advanced NSCLC, first-line treatment | **2018**: No additional benefit [2] | **2023:** 4 [57] |  | **2017:** Increase in remission rate, prolongation of PFS [112] |
| **2.** | Cobimetinib (Cotellic^®^) | **2015:** Metastatic melanoma with BRAF-V600-mutation | **2016:** Considerable additional benefit [3] | **2023:** 4 [58] |  | **2016:** Prolongation of PFS, OS and increase in remission rate [113] |
| **3a.** | Lenvatinib (Lenvima^®^) | **2015:** Metastatic thyroid carcinoma | **2019:** No additional benefit [4] | **2023:** 2 [59] |  | **2015:** Compared to placebo prolongation of PFS and OS [114] |
| **3b.** | Lenvatinib (Kisplyx^®^) | **2016:** Advanced renal cell carcinoma | **2021:** No additional benefit [5] | **2023:** 4 [60] |  | **2017:** Prolongation of PFS and OS, increase in remission rate, increased rate of severe events [115] |
| **3c.**  **Table S3** Overview of evaluations by GBA, ESMO, AkdÄ and DGHO | Lenvatinib  (Lenvima^®^) | **2018:** Advanced or unresectable hepatocellular carcinoma | **2019:** No additional benefit [6] |  |  | **2019:** Not inferior to appropriate comparative therapy in OS, increase in remission rate and prolongation of PFS, improvement in patient-reported outcome and quality of life parameters [116] |
| **3d.** | Lenvatinib  (Lenvima^®^) | **2021:** Endometrial carcinoma, previously treated with platinum-containing therapy, in combination with pembrolizumab | **2022:** Considerable additional benefit [7] | **2023:** 4 [61] |  | **2022:** Prolongation of OS and PFS, high rate of adverse events [117] |
| **3e.** | Lenvatinib (Kisplyx^®^) | **2021:** Advanced renal cell carcinoma, first-line treatment, in combination with pembrolizumab | **2022:** No additional benefit [8] | **2023:** 4 [62] |  | **2022:** Prolongation of OS and PFS [118] |
| **4.** | Nintedanib  (Vargatef^®^) | **2015:** Metastatic NSCLC | **2015:** Minor additional benefit [9] |  |  | **2015:** Prolongation of OS and PFS [119] |
| **5a.** | Trametinib (Mekinist^®^) | **2015:** Melanoma with BRAF-V600-mutation, in combination with dabrafenib | **2016:** Considerable additional benefit [10] | **2023:** 5 [63] |  | **2016:** Extension of remission rate, prolongation of PFS and OS [20] |
| **5b.**  **Table S3** (continued) | Trametinib (Mekinist^®^) | **2018:** Melanoma with BRAF-V600-mutation, in combination with dabrafenib, adjuvant therapy | **2019:** Considerable additional benefit [11] | **2023:** A [64] | **2019:** Considerable additional benefit [99] | **2019:** Compared to placebo prolongation of RFS, OS and increase in survival rate after 3 years [121] |
| **5c.** | Trametinib (Mekinist^®^) | **2017:**  Advanced NSCLC with BRAF-V600-mutation, in combination with dabrafenib | **2017:** No additional benefit [12] | **2023:** 2 [65] |  | **2017:** Prolongation of OS and PFS, increase in remission rate [122] |
| **6a.** | Osimertinib (Tagrisso^®^) | **2016:** Metastatic NSCLC with T790M-EGFR-mutation | **2017:** Considerable additional benefit [13] | **2023:** 4 [66] | **2017:** Considerable additional benefit [100] | **2017:** Increase in remission rate, prolongation of PFS, reduction in the rate of severe adverse events [123] |
| **6b.** | Osimertinib  (Tagrisso^®^) | **2019:** Metastatic NSCLC with T790M-EGFR-mutation, first-line treatment | **2019:** Considerable additional benefit [14] | **2023:** 4 [67] |  | **2018:** Prolongation of OS and PFS [124] |
| **6c.** | Osimertinib (Tagrisso^®^) | **2021:** Metastatic NSCLC with T790M-EGFR-mutation, adjuvant therapy, after complete tumor resection, pat. suitable for adjuvant platinum-based chemotherapy | **2021:** No additional benefit [15] | **2023:** A [68] |  | **2021:** Prolongation of disease-free survival [125] |
| **6d.**  **Table S3** (continued) | Osimertinib (Tagrisso^®^) | **2021:** Metastatic NSCLC with T790M-EGFR-mutation, adjuvant therapy, after complete tumor resection, pat. not suitable for adjuvant platinum-based chemotherapy | **2021:** Not-quantifiable additional benefit [15] | **2023:** A [68] |  | **2021:** Prolongation of disease-free survival [125] |
| **7.** | Palbociclib (Ibrance^®^) | **2016:** Hormone receptor-positive, HER2-negative, locally advanced or metastatic breast cancer | **2022:** No additional benefit [16], [17] | **2023:** 3 [69] | **2022:** No additional benefit [101] | **2019:** Prolongation of PFS, increase in remission rate, [126] |
| **8a.** | Alectinib (Alecensa^®^) | **2017:** ALK-positive, advanced NSCLC, previously treated with crizotinib | **2017:** Minor additional benefit [18] | **2023:** 4 [70] |  | **2017:** Prolongation of PFS, increase in remission rate, reduction of severe adverse events [127] |
| **8b.** | Alectinib  (Alecensa^®^) | **2017:** ALK-positive, advanced NSCLC, first-line treatment | **2018:** Not-quantifiable additional benefit [19] | **2023:** 4 [71] | **2018:** Not-quantifiable additional benefit [102] | **2018:** Prolongation of PFS, prolongation of time to CNS progression, reduction in the number of patients with CNS metastases [128] |
| **9a.** | Midostaurin (Rydapt^®^) | **2017:** AML with FLT3-mutation | **2018:** Considerable additional benefit, orphan drug [20] |  |  | **2018:** Prolongation of median OS and survival rate after 5 years [129] |
| **9b.**  **Table S3** (continued) | Midostaurin (Rydapt^®^) | **2017:** Aggressive systemic mastocytosis, systemic mastocytosis with associated haematological neoplasm or mast cell leukaemia | **2018:** Not-quantifiable additional benefit, orphan drug [21] |  |  | **2018:** Prolongation of PFS and OS in non-randomized trails [129] |
| **10a.** | Ribociclib (Kisqali^®^) | **2017:** Hormone receptor-positive, HER2-negative, locally advanced or metastatic breast cancer, in combination with an aromatase inhibitor | **2020:** Minor additional benefit [22] | **2023:** 4 [72] | **2019:** No additional benefit [103] | **2020:** Increase in remission rates, prolongation of PFS and OS [130] |
| **10b.** | Ribociclib (Kisqali^®^) | **2017:** Hormone receptor-positive, HER2-negative, locally advanced or metastatic breast cancer, in combination with fulvestrant | **2020:** Minor additional benefit [23] | **2023:** 4 [73] | **2019:** Not-quantifiable additional benefit [103] | **2019:** Increase in remission rates, prolongation of PFS and OS [131] |
| **11.** | Tivozanib (Fotivda^®^) | **2017:** Advanced renal cell carcinoma, first-line treatment | **2018:** No additional benefit [24] | **2023:** 3 [74] |  | **2018:** Increase in remission rate, prolongation of PFS [132] |
| **12a.**  **Table S3** (continued) | Abemaciclib  (Verzenios^®^) | **2018:** Hormone receptor-positive, HER2-negative, locally advanced or metastatic breast cancer, in combination with an aromatase inhibitor | **2019:** No additional benefit [25] | **2023:** 3 [75] | **2023:** Minor additional benefit [104] | **2023:** Increase in remission rate, prolongation of PFS, more frequent severe adverse events [133] |
| **12b.** | Abemaciclib (Verzenios^®^) | **2018:** Hormone receptor-positive, HER2-negative, locally advanced or metastatic breast cancer, in combination with fulvestrant | **2022:** No additional benefit [26] | **2023:** 4 [76] |  | **2022:** Increase in remission rate, prolongation of PFS and OS [134] |
| **12c.** | Abemaciclib (Verzenios^®^) | **2018:** Hormone receptor-positive, HER2-negative, locally advanced or metastatic breast cancer, in combination with Fulvestrant, postmenopausal pat., previously treated with endocrine therapy | **2022:** Minor additional benefit [26] | **2023:** 4 [76] |  | **2022:** Increase in remission rate, prolongation of PFS and OS [134] |
| **12d.**  **Table S3** (continued) | Abemaciclib (Verzenios^®^) | **2022:** Hormone receptor-positive, HER2-negative, node-positive breast cancer, combination with endocrine therapy, early stage with high risk of recurrence, premenopausal pat. | **2022:** Minor additional benefit [27] | **2023:** A [77] |  | **2022:** Prolongation of invasive disease-free survival, deterioration in quality of life, higher rate of severe adverse events [135] |
| **12e.** | Abemaciclib (Verzenios^®^) | **2022:** Hormone receptor-positive, HER2-negative, node-positive breast cancer, combination with endocrine therapy, early stage with high risk of recurrence, postmenopausal pat. and men | **2022:** No additional benefit [27] | **2023:** A [77] |  | **2022:** Prolongation of invasive disease-free survival, deterioration in quality of life, higher rate of severe adverse events [135] |
| **13.** | Binimetinib (Mektovi^®^) | **2018:** Melanoma with BRAF-V600-mutation, in combination with encorafenib | **2019**: No additional benefit [28] | **2023:** A/5 [78] |  | **2019:** Increase in remission rate, extension of time to deterioration of patient-relevant symptoms, prolongation of PFS and OS [136] |
| **14a.** | Encorafenib (Braftovi^®^) | **2018:** Melanoma with BRAF-V600-mutation, in combination with binimetinib | **2019:** No additional benefit [29] | **2023:** A/5 [78] |  | **2019:** Increase in remission rate, extension of time to deterioration of patient-relevant symptoms, prolongation of PFS and OS [137] |
| **14b.**  **Table S3** (continued) | Encorafenib (Braftovi^®^) | **2020:** Metastatic colorectal cancer with BRAF-V600-mutation after prior systemic therapy, in combination with cetuximab | **2020:** Considerable additional benefit [30] | **2023:** 4 [79] |  | **2020:** Increase of remission rate, prolongation of OS and PFS [138] |
| **15a.** | Brigatinib (Alunbrig^®^) | **2019:** ALK-positive, advanced NSCLC, previously treated with crizotinib | **2019:** No additional benefit [31] | **2023:** 3 [80] | **2019:** No additional benefit [105] | **2019:** High remission rate, long PFS and OS, high CNS efficacy, but no direct comparison with alectinib or ceritinib [139] |
| **15b.** | Brigatinib (Alunbrig^®^) | **2020:** ALK-positive, advanced NSCLC, previously not treated with an ALK inhibitor, with brain metastases | **2020:** Considerable additional benefit [32] | **2023:** 3 [81] |  | **2020:** Increase of remission rate, prolongation of PFS and OS [140] |
| **15c.** | Brigatinib (Alunbrig^®^) | **2020:** ALK-positive, advanced NSCLC, previously not treated with an ALK inhibitor, without brain metastases | **2020:** Minor additional benefit [32] | **2023:** 3 [81] |  | **2020:** Increase of remission rate, prolongation of PFS, [140] |
| **16.** | Dacomitinib (Vizimpro^®^) | **2019:** NSCLC with activating EGFR-mutations, first-line treatment | **2019:** No additional benefit [33] | **2023:** 3 [82] |  | **2019:** Prolongation of PFS and OS compared to gefitinib, high rate of adverse events [141] |
| **17.**  **Table S3** (continued) | Gilteritinib (Xospata^®^) | **2019:** AML with FLT3-mutation | **2020:** Considerable additional benefit, orphan drug [34] |  |  | **2020:** Increase in the rate of complete remissions, prolongation of median overall survival, lower rate of severe adverse events, no long-term increase in survival rate after >2 years [142] |
| **18.** | Larotrectinib (Vitrakvi^®^) | **2019:** Tumors that display a Neurotrophic Tyrosine Receptor Kinase gene fusion | **2020:** No additional benefit [35] | **2023:** 3 [83] | **2020:** No additional benefit [106] | **2020:** High response rates, rapid onset of action, data still incomplete due to the rarity of aberrations and their heterogeneity [143] |
| **19a.** | Lorlatinib (Lorviqua^®^) | **2019:** ALK-positive, advanced NSCLC, previously treated with an ALK inhibitor | **2019:** No additional benefit [36] | **2023:** 4 [84] | **2019:** No additional benefit [107] | **2019:** High remission rate, high efficacy in CNS metastases [144] |
| **19b.** | Lorlatinib (Lorviqua^®^) | **2022:** ALK-positive, advanced NSCLC, previously not treated with an ALK inhibitor | **2022:** No additional benefit [37] | **2023:** 4 [85] | **2022:** No additional benefit [108] | **2022:** Increase in remission rate, prolongation of PFS, higher efficacy in CNS metastases, increased rate of severe adverse events [145] |
| **20.**  **Table S3** (continued) | Neratinib (Nerlynx^®^) | **2019:** Hormone receptor-positive, HER2- overexpressed/amplified breast cancer | **2020:** Minor additional benefit [38] | **2023:** No evaluable benefit [86] |  | **2020:** Reduction in the risk of recurrence, prolongation of invasive disease-free survival, increased rate of severe adverse events [146] |
| **21a.** | Acalabrutinib (Calquence^®^) | **2020:** CLL, no 17p-deletion or TP53-mutation, pat. is suitable for FCR-therapy, monotherapy, first-line treatment | **2021:** No additional benefit [39] |  | **2021:** No additional benefit [109] | **2021:** Prolongation of PFS, lower rate of severe adverse events [147] |
| **21b.** | Acalabrutinib (Calquence^®^) | **2020:** CLL, no 17p-deletion or TP53-mutation, pat. is not suitable for FCR-therapy, monotherapy, first-line treatment | **2021:** Minor additional benefit [39] |  | **2021:** Not-quantifiable additional benefit [109] | **2021:** Prolongation of PFS, lower rate of severe adverse events [147] |
| **21c.** | Acalabrutinib (Calquence^®^) | **2020:** CLL, with 17p-deletion or TP53-mutation, monotherapy, first-line treatment | **2021:** No additional benefit [39] |  | **2021:** No additional benefit [109] | **2021:** Prolongation of PFS, lower rate of severe adverse events [147] |
| **21d.** | Acalabrutinib (Calquence^®^) | **2020:** CLL, no 17p-deletion or TP53-mutation, pat. is suitable for FCR-therapy, combination with obinutuzumab, first-line treatment | **2021:** No additional benefit [40] |  |  | **2021:** Prolongation of PFS, lower rate of severe adverse events [148] |
| **21e.**  **Table S3** (continued) | Acalabrutinib (Calquence^®^) | **2020:** CLL, no 17p-deletion or TP53-mutation, pat. is not suitable for FCR-therapy, combination with obinutuzumab, first-line treatment | **2021:** Minor additional benefit [40] |  |  | **2021:** Prolongation of PFS, lower rate of severe adverse events [148] |
| **21f.** | Acalabrutinib (Calquence^®^) | **2020:** CLL, with 17p-deletion or TP53-mutation, combination with obinutuzumab, first-line treatment | **2021:** No additional benefit [40] |  |  | **2021:** Prolongation of PFS, lower rate of severe adverse events [148] |
| **21g.** | Acalabrutinib (Calquence^®^) | **2020:** CLL, no 17p-deletion or TP53-mutation, after one previous therapy | **2021:** No additional benefit [41] |  |  | **2021:** Not-quantifiable [149] |
| **21h.** | Acalabrutinib (Calquence^®^) | **2020:** CLL, with 17p-deletion or TP53-mutation, after one previous therapy | **2021:** Considerable additional benefit [41] |  |  | **2021:** Not-quantifiable [149] |
| **21i.**  **Table S3** (continued) | Acalabrutinib (Calquence^®^) | **2020:** CLL, after at least two previous therapies, idelalisib in combination with rituximab or rituximab in combination with bendamustine is patient-specific appropriate therapy | **2021:** Minor additional benefit [41] |  |  | **2021:** Not-quantifiable [149] |
| **21j.** | Acalabrutinib (Calquence^®^) | **2020:** CLL, after at least two previous therapies, other therapy than idelalisib in combination with rituximab or rituximab in combination with bendamustine is patient-specific appropriate therapy | **2021:** No additional benefit [41] |  |  | **2021:** Not-quantifiable [149] |
| **22a.** | Alpelisib (Piqray^®^) | **2020:** Hormone receptor-positive, HER2-negative, locally advanced or metastatic breast cancer with PIK3CA-mutation, following endocrine therapy in the (neo-) adjuvant therapy situation, postmenopausal patients without liver or lung metastases | **2021:** Minor additional benefit [42] | **2023:** 2 [87] |  | **2020:** Increase in remission rate, prolongation of PFS, increase in the rate of severe adverse events [150] |
| **22b.**  **Table S3** (continued) | Alpelisib (Piqray^®^) | **2020:** Hormone receptor-positive, HER2-negative, locally advanced or metastatic breast cancer with PIK3CA-mutation, following endocrine therapy in the (neo-) adjuvant therapy situation, postmenopausal patients with liver or lung metastases | **2021:** No additional benefit [42] | **2023:** 2 [87] |  | **2020:** Increase in remission rate, prolongation of PFS, increase in the rate of severe adverse events [150] |
| **22c.** | Alpelisib (Piqray^®^) | **2020:** Hormone receptor-positive, HER2-negative, locally advanced or metastatic breast cancer with PIK3CA-mutation, postmenopausal pat., following endocrine therapy in locally advanced or metastatic stage | **2021:** Minor additional benefit [42] | **2023:** 2 [87] |  | **2020:** Increase in remission rate, prolongation of PFS, increase in the rate of severe adverse events [150] |
| **22.d**  **Table S3** (continued) | Alpelisib (Piqray^®^) | **2020:** Hormone receptor-positive, HER2-negative, locally advanced or metastatic breast cancer with PIK3CA-mutation, men | **2021:** No additional benefit [42] | **2023:** 2 [87] |  | **2020:** Increase in remission rate, prolongation of PFS, increase in the rate of severe adverse events [150] |
| **23a.** | Avapritinib (Ayvakyt^®^) | **2020:** Unresectable or metastatic GIST with platelet-derived growth factor receptor alpha D842V mutation | **2021:** Not-quantifiable additional benefit, orphan drug [43] | **2023:** 3 [88] |  | **2021:** Prolongation of PFS [151] |
| **23b.** | Avapritinib (Ayvakyt^®^) | **2022:** aggressive systemic mastocytosis, systemic mastocytosis with associated haematological neoplasm or mast cell leukaemia, after at least one previous systemic therapy | **2022:** Not-quantifiable additional benefit, orphan drug [44] |  |  | **2022:** Not-quantifiable [152] |
| **24a.** | Entrectinib (Rozlytrek^®^) | **2020:** Tumors that display a Neurotrophic Tyrosine Receptor Kinase gene fusion | **2021:** No additional benefit [45] | **2023:** 3 [89] | **2020:** No additional benefit [110] | **2020:** High response rates, rapid onset of action, data still incomplete due to the rarity of aberrations and their heterogeneity [153] |
| **24b.**  **Table S3** (continued) | Entrectinib (Rozlytrek^®^) | **2020:** ROS1-positve, advanced NSCLC | **2021:** No additional benefit [46] | **2023:** 3 [90] |  | **2020:** Results not conclusive with regard to the quantification of added benefit, high efficacy in CNS metastases [154] |
| **25.** | Fedratinib (Inrebic^®^) | **2021:** Myelofibrosis | **2021:** Not-quantifiable additional benefit, orphan drug [47] |  |  | **2021:** Reduction in spleen size and reduction of myelofibrosis-associated symptoms [155] |
| **26.** | Pemigatinib (Pemazyre^®^) | **2021:** Locally advanced or metastatic cholangiocarcinoma with FGFR2 fusion | **2021:** Not-quantifiable additional benefit, orphan drug [48] | **2023:** 3 [91] |  | **2021:** Therapy of choice for patients with biliary carcinoma and evidence of FGFR2 gene fusion [156] |
| **27a.** | Selpercatinib (Retsevmo^®^) | **2021:** Advanced NSCLC with RET-mutation | **2021:** No additional benefit [49] | **2023:** 3 [92] |  | **2021:** Higher response rate, longer PFS, high efficacy in CNS metastases [157] |
| **27b.** | Selpercatinib (Retsevmo^®^) | **2021:** Advanced thyroid cancer with RET-mutation, previous treated with cabozantinib and/or vandetanib | **2021:** No additional benefit [50] | **2023:** 3 [93] |  | **2021:** High response rate, sustained remissions, high efficacy in CNS metastases, lower rate of severe adverse events [158] |
| **27c.** | Selpercatinib (Retsevmo^®^) | **2021:** Advanced thyroid cancer with RET-mutation, previous treated with sorafenib and/or lenvatinib | **2021:** No additional benefit [51] | **2023:** 3 [94] |  | **2021:** Increase in remission rate, more favorable adverse event profile [159] |
| **27d.**  **Table S3** (continued) | Selpercatinib (Retsevmo^®^) | **2022:** Advanced NSCLC with RET-mutation, first-line treatment | **2022:** No additional benefit [52] | **2023:** 3 [95] |  | **2022:** Not-quantifiable [160] |
| **27e.** | Selpercatinib (Retsevmo^®^) | **2023:** Advanced thyroid cancer, with RET-mutation, monotherapy | **2023:** No additional benefit [53] | **2023:** 3 [96] |  | **2023:** Very probable additional benefit [161] |
| **28.** | Selumetinib (Koselugo^®^) | **2021:** Symptomatic, inoperable plexiform neurofibromas in paediatric patients with neurofibromatosis type 1 | **2022:** Not-quantifiable additional benefit, orphan drug [54] |  |  |  |
| **29.** | Tucatinib (Tukysa^®^) | **2021:** HER2-positive locally advanced or metastatic breast cancer in combination with trastuzumab and capecitabin | **2021:** Considerable additional benefit [55] | **2023:** 4 [97] |  | **2021:** Increase in remission rate, prolongation of PFS and OS, high efficacy in CNS metastases, increase in the rate of severe adverse events [162] |

**Table S3** (continued)

|  | **Drug** | **Mechanism of Action** | **Target** | **Action** | **pIC50** |
| --- | --- | --- | --- | --- | --- |
| **1.** | Ceritinib | ALK inhibitor | ALK | Inhibition | 9.7 |
|  |  |  | Insulin receptor | Inhibition | 8.1 |
|  |  |  | IGF1 | Inhibition | 8.1 |
|  |  |  | FLT3 | Inhibition | 7.2 |
|  |  |  | TSSK1B | Inhibition | 7.6 |
| **2.** | Cobimetinib | MEK inhibitor | MEK1 | Negative allosteric modulation | 9.1 |
|  |  |  | MEK2 | Negative allosteric modulation | 6.7 |
|  |  |  | MEK7 | Negative allosteric modulation | <5.0 |
| **3.** | Lenvatinib | Multiple kinase inhibitor | VEGFR-2 | Inhibition | 8.4 |
|  |  |  | VEGFR-3 | Inhibition | 8.3 |
| **4.**  **Table S4** Pharmacological characterization of analyzed drugs | Nintedanib | Angiokinase inhibitor | VEGFR-3 | Inhibition | 7.9 |
|  |  |  | VEGFR-2 | Inhibition | 7.7 |
|  |  |  | VEGFR-1 | Inhibition | 7.5 |
|  |  |  | FGFR2 | Inhibition | 7.4 |
|  |  |  | PDGFR α | Inhibition | 7.2 |
|  |  |  | PDGFR β | Inhibition | 7.2 |
|  |  |  | FGFR1 | Inhibition | 7.2 |
|  |  |  | FGFR3 | Inhibition | 7.0 |
|  |  |  | FGFR4 | Inhibition | 6.2 |
| **5.** | Trametinib | MEK inhibitor | MEK1 | Inhibition | 9.0-9.1 |
|  |  |  | MEK2 | Inhibition | 8.7 |
| **6.** | Osimertinib | EGFR inhibitor | EGFR | Inhibition | 6.3 |
| **7.** | Palbociclib | CDK inhibitor | CDK4 | Inhibition | 7.4-8.5 |
|  |  |  | CDK6 | Inhibition | 7.4-8.0 |
| **8.** | Alectinib | ALK inhibitor | ALK | Inhibition | 8.7 |
| **9.** | Midostaurin | Multiple kinase inhibitor | FLT3 | Inhibition | 6.3 |
| **10.** | Ribociclib | CDK inhibitor | CDK4 | Inhibition | 8.0 |
| **11.** | Tivozanib | VEGFR inhibitor | VEGFR-2 | Inhibition | 9.8 |
|  |  |  | VEGFR-1 | Inhibition | 9.7 |
|  |  |  | VEGFR-3 | Inhibition | 9.6 |
| **12.** | Abemaciclib | CDK inhibitor | CDK4 | Inhibition | 8.7 |
|  |  |  | CDK6 | Inhibition | 8.0 |
| **13.** | Binimetinib | MEK inhibitor | MEK1 | Negative allosteric modulation | 7.9 |
|  |  |  | MEK2 | Negative allosteric modulation | 7.9 |
| **14.**  **Table S4** (continued) | Encorafenib | BRAF inhibitor | BRAF-V600 | Inhibition | 8.4 |
| **15.** | Brigatinib | ALK inhibitor | ALK | Inhibition | 9.2-9.4 |
|  |  |  | IGF1R | Inhibition | 7.6 |
|  |  |  | EGFR | Inhibition | 6.9 |
|  |  |  | Insulin receptor | Inhibition | 6.7 |
| **16.** | Dacomitinib | EGFR inhibitor | EGFR | Inhibition | 8.2 |
|  |  |  | HER2/neu | Inhibition | 7.3 |
|  |  |  | ERBB4 | Inhibition | 7.1 |
| **17.** | Gilteritinib | FLT3 inhibitor | LTK | Inhibition | 9.7 |
|  |  |  | FLT3 | Inhibition | 9.5 |
|  |  |  | ALK | Inhibition | 9.3 |
|  |  |  | AXL | Inhibition | 9.1 |
|  |  |  | NTRK1 | Inhibition | 9.0 |
|  |  |  | ROS1 | Inhibition | 8.8 |
|  |  |  | Ret proto-oncogene | Inhibition | 8.8 |
|  |  |  | MERTK | Inhibition | 8.5 |
| **18.** | Larotrectinib | TRK inhibitor | NTRK1 | Inhibition | 8.0 |
| **19.**  **Table S4** (continued) | Lorlatinib | ALK inhibitor | FES | Inhibition | 8.2 |
|  |  |  | ROS1 | Inhibition | 11.3 (pKi) |
|  |  |  | ALK | Inhibition | 9.1 (pKi) |
| **20.** | Neratinib | HER2 inhibitor | HER2/neu | Inhibition | 7.2 |
|  |  |  | EGFR | Inhibition | 7.0 |
| **21.** | Acalabrutinib | BTK inhibitor | BTK | Inhibition | 8.3 |
|  |  |  | BMX | Inhibition | 7.3 |
|  |  |  | TEC | Inhibition | 7.0 |
|  |  |  | TXK | Inhibition | 6.4 |
|  |  |  | BLK | Inhibition | <6.0 |
|  |  |  | ITK | Inhibition | <6.0 |
|  |  |  | JAK3 | Inhibition | <6.0 |
|  |  |  | LYN | Inhibition | <6.0 |
|  |  |  | ERBB4 | Inhibition | 7.8 |
|  |  |  | EGFR | Inhibition | <6.0 |
|  |  |  | HER2/neu | Inhibition | <6.0 |
| **22.** | Alpelisib | PIK3 inhibitor | PIK3CA | Inhibition | 8.3 |
|  |  |  | PIK3CG | Inhibition | 6.6 |
|  |  |  | PIK3CD | Inhibition | 6.5 |
|  |  |  | PIK3CB | Inhibition | 5.9 |
| **23.**  **Table S4** (continued) | Avapritinib | KIT inhibitor | KIT | Inhibition | 7.1 |
| **24.** | Entrectinib | TRK inhibitor | ALK | Inhibition | 1nM (K_i_) |
|  |  |  | NTRK1 | Inhibition | 1.58nM (K_i_) |
|  |  |  | NTRK2 | Inhibition | 2.51nM (K_i_) |
|  |  |  | NTRK3 | Inhibition | 1.58nM (K_i_) |
| **25.** | Fedratinib | JAK inhibitor | JAK2 | Inhibition | 8.5 |
|  |  |  | JAK1 | Inhibition | 7.0 |
|  |  |  | TYK2 | Inhibition | 6.8 |
|  |  |  | JAK3 | Inhibition | 6.0 |
| **26.** | Pemigatinib | FGFR inhibitor | FGFR1 | Inhibition | >7.0 |
|  |  |  | FGFR2 | Inhibition | >7.0 |
|  |  |  | FGFR3 | Inhibition | >7.0 |
| **27.** | Selpercatinib | RET inhibitor | RET | Inhibition | 7.8 |
| **28.** | Selumetinib | MEK inhibitor | MEK1 | Negative allosteric modulation | 7.8-7.9 |
| **29.** | Tucatinib | HER2 inhibitor | HER2/neu | Inhibition | 8.2 |
|  |  |  | ERBB4 | Inhibition | 6.5 |
|  |  |  | EGFR | Inhibition | 6.3 |

**Table S4** (continued)

**Abbreviations:**

ALK ALK receptor tyrosine kinase

AXL AXL receptor tyrosine kinase

BLK B lymphocyte kinase

BMX BMX non-receptor tyrosine kinase

BRAF-V600 BRAF^V600E^ kinase

BTK bruton´s tyrosine kinase

CDK4 cyclin dependent kinase 4

CDK6 cyclin dependent kinase 6

EGFR epidermal growth factor receptor

ERBB4 erb-b2 receptor tyrosine kinase 4

FES FES proto-oncogene, receptor tyrosine kinase

FGFR1 fibroblast growth factor receptor 1

FGFR2 fibroblast growth factor receptor 2

FGFR3 fibroblast growth factor receptor 3

FGFR4 fibroblast growth factor receptor 4

FLT3 Fms related receptor tyrosine kinase 3

HER2/neu human epidermal growth factor receptor-2

IGF1 Insulin-like growth factor I receptor

ITK interleukin-2-inducible T-cell kinase

JAK1 Janus kinase 1

JAK2 Janus kinase 2

JAK3 Janus kinase 3

KIT KIT protein tyrosine kinase

LTK leukocyte receptor tyrosine kinase

LYN LYN protein tyrosine kinase

MEK1 mitogen-activated protein kinase 1

MEK2 mitogen-activated protein kinase 2

MEK7 mitogen-activated protein kinase 7

MERTK MER proto-oncogene, tyrosine kinase

NTRK1 neurotrophic receptor kinase 1

NTRK2 neurotrophic receptor kinase 2

NTRK3 neurotrophic receptor kinase 3

PDGFR α platelet derived growth factor receptor alpha

PDGFR β platelet derived growth factor receptor beta

PIK3CA phosphatidylinositol-4,5-biphosphate 3-kinase catalytic subunit alpha

PIK3CB phosphatidylinositol-4,5-biphosphate 3-kinase catalytic subunit beta

PIK3CD phosphatidylinositol-4,5-biphosphate 3-kinase catalytic subunit delta

PIK3CG phosphatidylinositol-4,5-biphosphate 3-kinase catalytic subunit gamma

RET RET protein tyrosine kinase

ROS1 c-ros oncogene 1, receptor tyrosine kinase

TEC TEC protein tyrosine kinase

TRK tropomyosin receptor kinase

TXK TXK tyrosine kinase

TYK2 tyrosine kinase 2

TSSK1B testis specific serine kinase 1B

VEGFR-1 vascular endothelial growth factor receptor 1

VEGFR-2 vascular endothelial growth factor receptor 2

VEGFR-3 vascular endothelial growth factor receptor 3

**References Table S2:**

[1]: Gemeinsamer Bundesausschuss (GBA) (2015) Beschluss des Gemeinsamen Bundesausschusses über eine Änderung der Arzneimittel-Richtlinie (AM-RL): Anlage XII - Beschlüsse über die Nutzenbewertung von Arzneimitteln mit neuen Wirkstoffen nach § 35a SGB V - Ceritinib. https://www.g-ba.de/downloads/39-261-2414/2015-12-17_AM-RL-XII_Ceritinib_2015-07-01-D-171_BAnz.pdf. Accessed 29 March 2023

[2]: Gemeinsamer Bundesausschuss (GBA) (2017) Beschluss des Gemeinsamen Bundesausschusses über eine Änderung der Arzneimittel-Richtlinie (AM-RL): Anlage XII - Beschlüsse über die Nutzenbewertung von Arzneimitteln mit neuen Wirkstoffen nach § 35a SGB V - Ceritinib (Ablauf der Befristung). https://www.g-ba.de/downloads/39-261-2876/2017-03-16_AM-RL-XII_Ceritinib_D-259_BAnz.pdf. Accessed 29 March 2023

[3]: Gemeinsamer Bundesausschuss (GBA) (2018) Beschluss des Gemeinsamen Bundesausschusses über eine Änderung der Arzneimittel-Richtlinie (AM-RL): Anlage XII - Beschlüsse über die Nutzenbewertung von Arzneimitteln mit neuen Wirkstoffen nach § 35a SGB V - Ceritinib (neues Anwendungsgebiet: Erstlinienbehandlung, nicht-kleinzelliges Lungenkarzinom). https://www.g-ba.de/downloads/39-261-3214/2018-02-01_AM-RL-XII_Ceritinib_nAWG_D-296_BAnz.pdf. Accessed 29 March 2023

[4]: Gemeinsamer Bundesausschuss (GBA) (2016) Beschluss des Gemeinsamen Bundesausschusses über eine Änderung der Arzneimittel-Richtlinie (AM-RL): Anlage XII - Beschlüsse über die Nutzenbewertung von Arzneimitteln mit neuen Wirkstoffen nach § 35a SGB V - Cobimetinib. https://www.g-ba.de/downloads/39-261-2607/2016-06-02_AM-RL-XII_Cobimetinib_D-196_BAnz.pdf. Accessed 29 March 2023

[5]: Gemeinsamer Bundesausschuss (GBA) (2015) Beschluss des Gemeinsamen Bundesausschusses über eine Änderung der Arzneimittel-Richtlinie (AM-RL): Anlage XII - Beschlüsse über die Nutzenbewertung von Arzneimitteln mit neuen Wirkstoffen nach § 35a SGB V - Lenvatinib. https://www.g-ba.de/downloads/39-261-2413/2015-12-17_AM-RL-XII_Lenvatinib_2015-07-01-D-164_BAnz.pdf. Accessed 29 March 2023

[6]: Gemeinsamer Bundesausschuss (GBA) (2015) Beschluss des Gemeinsamen Bundesausschusses über eine Änderung der Arzneimittel-Richtlinie (AM-RL): Anlage XII - Nutzenbewertung von Arzneimitteln mit neuen Wirkstoffen nach § 35a SGB V Lenvatinib (Bewertung nach Aufhebung des Orphan Drug-Status). https://www.g-ba.de/downloads/39-261-3922/2019-08-15_AM-RL-XII_Lenvatinib_D-428_BAnz.pdf. Accessed 29 March 2023

[7]: Gemeinsamer Bundesausschuss (GBA) (2017) Beschluss des Gemeinsamen Bundesausschusses über eine Änderung der Arzneimittel-Richtlinie (AM-RL): Anlage XII - Beschlüsse über die Nutzenbewertung von Arzneimitteln mit neuen Wirkstoffen nach § 35a SGB V - Lenvatinib (neues Anwendungsgebiet: fortgeschrittenes Nierenzellkarzinom). https://www.g-ba.de/downloads/39-261-2878/2017-03-16_AM-RL-XII_Lenvatinib_D-257_BAnz.pdf. Accessed 29 March 2023

[8]: Gemeinsamer Bundesausschuss (GBA) (2021) Beschluss des Gemeinsamen Bundesausschusses über eine Änderung der Arzneimittel-Richtlinie (AM-RL): Anlage XII - Nutzenbewertung von Arzneimitteln mit neuen Wirkstoffen nach § 35a SGB V, Lenvatinib (Neubewertung nach Fristablauf: fortgeschrittenes Nierenzellkarzinom, Kombination mit Everolimus). https://www.g-ba.de/downloads/39-261-4908/2021-07-01_AM-RL-XII_Lenvatinib_D-620_BAnz.pdf. Accessed 29 March 2023

[9]: Gemeinsamer Bundesausschuss (GBA) (2019) Beschluss des Gemeinsamen Bundesausschusses über eine Änderung der Arzneimittel-Richtlinie (AM-RL): Anlage XII - Beschlüsse über die Nutzenbewertung von Arzneimitteln mit neuen Wirkstoffen nach § 35a SGB V - Lenvatinib (neues Anwendungsgebiet Hepatozelluläres Karzinom). https://www.g-ba.de/downloads/39-261-3715/2019-03-22_AM-RL-XII_Lenvatinib-nAWG_D-379_BAnz.pdf. Accessed 29 March 2023

[10]: Gemeinsamer Bundesausschuss (GBA) (2022) Beschluss des Gemeinsamen Bundesausschusses über eine Änderung der Arzneimittel-Richtlinie: Anlage XII - Nutzenbewertung von Arzneimitteln mit neuen Wirkstoffen nach § 35a des Fünften Buches Sozialgesetzbuch (SGB V) Lenvatinib (neues Anwendungsgebiet: Endometriumkarzinom, nach vorheriger Platin-basierter Therapie, Kombination mit Pembrolizumab). https://www.g-ba.de/downloads/39-261-5518/2022-07-07_AM-RL-XII_Lenvantinib_D-755_BAnz.pdf. Accessed 29 March 2023

[11]: Gemeinsamer Bundesausschuss (GBA) (2022) Beschluss des Gemeinsamen Bundesausschusses über eine Änderung der Arzneimittel-Richtlinie: Anlage XII - Nutzenbewertung von Arzneimitteln mit neuen Wirkstoffen nach § 35a des Fünften Buches Sozialgesetzbuch (SGB V) Lenvatinib (neues Anwendungsgebiet: fortgeschrittenes Nierenzellkarzinom, Erstlinie, Kombination mit Pembrolizumab). https://www.g-ba.de/downloads/39-261-5521/2022-07-07_AM-RL-XII_Lenvatinib_D-749_BAnz.pdf. Accessed 29 March 2023

[12]: Gemeinsamer Bundesausschuss (GBA) (2015) Beschluss des Gemeinsamen Bundesausschusses über eine Änderung der Arzneimittel-Richtlinie (AM-RL): Anlage XII - Beschlüsse über die Nutzenbewertung von Arzneimitteln mit neuen Wirkstoffen nach § 35a SGB V - Nintedanib. https://www.g-ba.de/downloads/39-261-2262/2015-06-18_AM-RL-XII_Nintedanib_2015-01-01-D-147_BAnz.pdf. Accessed 29 March 2023

[13]: Gemeinsamer Bundesausschuss (GBA) (2016) Beschluss des Gemeinsamen Bundesausschusses über eine Änderung der Arzneimittel-Richtlinie (AM-RL): Anlage XII - Beschlüsse über die Nutzenbewertung von Arzneimitteln mit neuen Wirkstoffen nach § 35a SGB V - Trametinib. https://www.g-ba.de/downloads/39-261-2531/2016-03-17_AM-RL-XII_Trametinib_2015-10-01-D-183_BAnz.pdf. Accessed 29 March 2023

[14]: Gemeinsamer Bundesausschuss (GBA) (2019) Beschluss des Gemeinsamen Bundesausschusses über eine Änderung der Arzneimittel-Richtlinie (AM-RL): Anlage XII - Beschlüsse über die Nutzenbewertung von Arzneimitteln mit neuen Wirkstoffen nach § 35a SGB V - Trametinib (neues Anwendungsgebiet: Melanom, in Kombination mit Dabrafenib, BRAF-V600-Mutation, adjuvante Behandlung). https://www.g-ba.de/downloads/39-261-3720/2019-03-22_AM-RL-XII_Trametinib_D-384_BAnz.pdf. Accessed 29 March 2023

[15]: Gemeinsamer Bundesausschuss (GBA) (2017) Beschluss des Gemeinsamen Bundesausschusses über eine Änderung der Arzneimittel-Richtlinie (AM-RL): Anlage XII - Beschlüsse über die Nutzenbewertung von Arzneimitteln mit neuen Wirkstoffen nach § 35a SGB V - Trametinib (neues Anwendungsgebiet: nicht-kleinzelliges Lungenkarzinom). https://www.g-ba.de/downloads/39-261-3095/2017-10-19_AM-RL-XII_Trametinib_D-284_BAnz.pdf. Accessed 29 March 2023

[16]: Gemeinsamer Bundesausschuss (GBA) (2016) Beschluss des Gemeinsamen Bundesausschusses über eine Änderung der Arzneimittel-Richtlinie (AM-RL): Anlage XII - Beschlüsse über die Nutzenbewertung von Arzneimitteln mit neuen Wirkstoffen nach § 35a SGB V - Osimertinib. https://www.g-ba.de/downloads/39-261-2700/2016-09-15_AM-RL-XII_Osimertinib_D-219_BAnz.pdf. Accessed 29 March 2023

[17]: Gemeinsamer Bundesausschuss (GBA) (2017) Beschluss des Gemeinsamen Bundesausschusses über eine Änderung der Arzneimittel-Richtlinie (AM-RL): Anlage XII - Beschlüsse über die Nutzenbewertung von Arzneimitteln mit neuen Wirkstoffen nach § 35a SGB V - Osimertinib (Ablauf der Befristung). https://www.g-ba.de/downloads/39-261-3092/2017-10-19_AM-RL-XII_Osimertinib_D-282_BAnz.pdf. Accessed 29 March 2023

[18]: Gemeinsamer Bundesausschuss (GBA) (2019) Beschluss des Gemeinsamen Bundesausschusses über eine Änderung der Arzneimittel-Richtlinie (AM-RL): Anlage XII - Beschlüsse über die Nutzenbewertung von Arzneimitteln mit neuen Wirkstoffen nach § 35a SGB V - Osimertinib (neues Anwendungsgebiet: Erstlinientherapie des lokal fortgeschrittenen oder metastasierten nicht-kleinzelligen Lungenkarzinoms). https://www.g-ba.de/downloads/39-261-3646/2019-01-17_AM-RL-XII_Osimertinib_D-369_BAnz.pdf. Accessed 29 March 2023

[19]: Gemeinsamer Bundesausschuss (GBA) (2021) Beschluss des Gemeinsamen Bundesausschusses über eine Änderung der Arzneimittel-Richtlinie (AM-RL): Anlage XII - Beschlüsse über die Nutzenbewertung von Arzneimitteln mit neuen Wirkstoffen nach § 35a SGB V - Osimertinib (neues Anwendungsgebiet: Nicht-kleinzelliges Lungenkarzinom, EGFR Mutation, adjuvante Therapie). https://www.g-ba.de/downloads/39-261-5177/2021-12-16_AM-RL-XII_Osimertinib_D-701.pdf. Accessed 29 March 2023

[20]: Gemeinsamer Bundesausschuss (GBA) (2017) Beschluss des Gemeinsamen Bundesausschusses über eine Änderung der Arzneimittel-Richtlinie (AM-RL): Anlage XII - Beschlüsse über die Nutzenbewertung von Arzneimitteln mit neuen Wirkstoffen nach § 35a SGB V - Palbociclib. https://www.g-ba.de/downloads/39-261-2947/2017-05-18_AM-RL-XII_Palbociclib_D-264_BAnz.pdf. Accessed 29 March 2023

[21]: Gemeinsamer Bundesausschuss (GBA) (2019) Beschluss des Gemeinsamen Bundesausschusses über eine Änderung der Arzneimittel-Richtlinie (AM-RL): Anlage XII - Beschlüsse über die Nutzenbewertung von Arzneimitteln mit neuen Wirkstoffen nach § 35a SGB V - Palbociclib (Brustkrebs; in Kombination mit Fulvestrant nach endokriner Therapie; Neubewertung nach Fristablauf). https://www.g-ba.de/downloads/39-261-3713/2019-03-22_AM-RL-XII_Palbociclib_D-395_BAnz.pdf. Accessed 29 March 2023

[22]: Gemeinsamer Bundesausschuss (GBA) (2022) Beschluss des Gemeinsamen Bundesausschusses über eine Änderung der Arzneimittel-Richtlinie: Anlage XII - Nutzenbewertung von Arzneimitteln mit neuen Wirkstoffen nach § 35a des Fünften Buches Sozialgesetzbuch (SGB V) Palbociclib (Neubewertung nach Fristablauf: Mammakarzinom, HR+, HER2-, Kombination mit Aromatasehemmern). https://www.g-ba.de/downloads/39-261-5773/2022-12-15_AM-RL-XII_Palbociclib_D-834_BAnz.pdf. Accessed 29 March 2023

[23]: Gemeinsamer Bundesausschuss (GBA) (2017) Beschluss des Gemeinsamen Bundesausschusses über eine Änderung der Arzneimittel-Richtlinie (AM-RL): Anlage XII - Beschlüsse über die Nutzenbewertung von Arzneimitteln mit neuen Wirkstoffen nach § 35a SGB V - Alectinib. https://www.g-ba.de/downloads/39-261-3090/2017-10-19_AM-RL-XII_Alectinib_D-281_BAnz.pdf. Accessed 29 March 2023

[24]: Gemeinsamer Bundesausschuss (GBA) (2018) Beschluss des Gemeinsamen Bundesausschusses über eine Änderung der Arzneimittel-Richtlinie (AM-RL): Anlage XII - Beschlüsse über die Nutzenbewertung von Arzneimitteln mit neuen Wirkstoffen nach § 35a SGB V - Alectinib (neues Anwendungsgebiet: Erstlinienbehandlung nicht-kleinzelliges Lungenkarzinom). https://www.g-ba.de/downloads/39-261-3368/2018-06-21_AM-RL-XII_Alectinib_D-326_BAnz.pdf. Accessed 29 March 2023

[25]: Gemeinsamer Bundesausschuss (GBA) (2018) Beschluss des Gemeinsamen Bundesausschusses über eine Änderung der Arzneimittel-Richtlinie (AM-RL): Anlage XII - Beschlüsse über die Nutzenbewertung von Arzneimitteln mit neuen Wirkstoffen nach § 35a SGB V - Midostaurin (akute myeloische Leukämie). https://www.g-ba.de/downloads/39-261-3277/2018-04-05_AM-RL-XII_Midostaurin_AML_D-319_BAnz.pdf. Accessed 29 March 2023

[26]: Gemeinsamer Bundesausschuss (GBA) (2018) Beschluss des Gemeinsamen Bundesausschusses über eine Änderung der Arzneimittel-Richtlinie (AM-RL): Anlage XII - Beschlüsse über die Nutzenbewertung von Arzneimitteln mit neuen Wirkstoffen nach § 35a SGB V - Midostaurin (aggressive systemische Mastozytose). https://www.g-ba.de/downloads/39-261-3279/2018-04-05_AM-RL-XII_Midostaurin_ASM_D-319_BAnz.pdf. Accessed 29 March 2023

[27]: Gemeinsamer Bundesausschuss (GBA) (2018) Beschluss des Gemeinsamen Bundesausschusses über eine Änderung der Arzneimittel-Richtlinie (AM-RL): Anlage XII - Beschlüsse über die Nutzenbewertung von Arzneimitteln mit neuen Wirkstoffen nach § 35a SGB V - Ribociclib. https://www.g-ba.de/downloads/39-261-3253/2018-03-16_AM-RL-XII_Ribociclib_D-307_BAnz.pdf. Accessed 29 March 2023

[28]: Gemeinsamer Bundesausschuss (GBA) (2020) Beschluss des Gemeinsamen Bundesausschusses über eine Änderung der Arzneimittel-Richtlinie (AM-RL): Anlage XII - Nutzenbewertung von Arzneimitteln mit neuen Wirkstoffen nach § 35a SGB V - Ribociclib (Neubewertung nach Fristablauf (Mammakarzinom, HR+, HER2-, Kombination mit einem Aromatasehemmer)). https://www.g-ba.de/downloads/39-261-4423/2020-08-20_AM-RL-XII_Ribociclib_D-517_BAnz.pdf. Accessed 29 March 2023

[29]: Gemeinsamer Bundesausschuss (GBA) (2019) Beschluss des Gemeinsamen Bundesausschusses über eine Änderung der Arzneimittel-Richtlinie (AM-RL): Anlage XII - Beschlüsse über die Nutzenbewertung von Arzneimitteln mit neuen Wirkstoffen nach § 35a SGB V - Ribociclib (neues Anwendungsgebiet: Brustkrebs, in Kombination mit Fulvestrant). https://www.g-ba.de/downloads/39-261-3863/2019-07-04_AM-RL-XII_Ribociclib-Fulvestrant_D-430_BAnz.pdf. Accessed 29 March 2023

[30]: Gemeinsamer Bundesausschuss (GBA) (2020) Beschluss des Gemeinsamen Bundesausschusses über eine Änderung der Arzneimittel-Richtlinie (AM-RL): Anlage XII - Nutzenbewertung von Arzneimitteln mit neuen Wirkstoffen nach § 35a SGB V - Ribociclib (Neubewertung nach Fristablauf (Mammakarzinom, HR+, HER2-, Kombination mit Fulvestrant)). https://www.g-ba.de/downloads/39-261-4428/2020-08-20_AM-RL-XII_Ribociclib_D-518_BAnz.pdf. Accessed 29 March 2023

[31]: Gemeinsamer Bundesausschuss (GBA) (2018) Beschluss des Gemeinsamen Bundesausschusses über eine Änderung der Arzneimittel-Richtlinie (AM-RL): Anlage XII - Beschlüsse über die Nutzenbewertung von Arzneimitteln mit neuen Wirkstoffen nach § 35a SGB V - Tivozanib. https://www.g-ba.de/downloads/39-261-3291/2018-04-19_AM-RL-XII_Tivozanib_D-323_BAnz.pdf. Accessed 29 March 2023

[32]: Gemeinsamer Bundesausschuss (GBA) (2019) Beschluss des Gemeinsamen Bundesausschusses über eine Änderung der Arzneimittel-Richtlinie (AM-RL): Anlage XII - Beschlüsse über die Nutzenbewertung von Arzneimitteln mit neuen Wirkstoffen nach § 35a SGB V - Abemaciclib (Brustkrebs; in Kombination mit einem Aromatasehemmer). https://www.g-ba.de/downloads/39-261-3767/2019-05-02_AM-RL-XII_Abemaciclib_D-400_BAnz.pdf. Accessed 29 March 2023

[33]: Gemeinsamer Bundesausschuss (GBA) (2019) Beschluss des Gemeinsamen Bundesausschusses über eine Änderung der Arzneimittel-Richtlinie (AM-RL): Anlage XII - Beschlüsse über die Nutzenbewertung von Arzneimitteln mit neuen Wirkstoffen nach § 35a SGB V - Abemaciclib (Brustkrebs; in Kombination mit Fulvestrant). https://www.g-ba.de/downloads/39-261-3768/2019-05-02_AM-RL-XII_Abemaciclib_D-401_BAnz.pdf. Accessed 29 March 2023

[34]: Gemeinsamer Bundesausschuss (GBA) (2022) Beschluss des Gemeinsamen Bundesausschusses über eine Änderung der Arzneimittel-Richtlinie: Anlage XII - Nutzenbewertung von Arzneimitteln mit neuen Wirkstoffen nach § 35a des Fünften Buches Sozialgesetzbuch (SGB V) Abemaciclib (Neubewertung nach Fristablauf: Mammakarzinom, HR+, HER2-, Kombination mit Fulvestrant). https://www.g-ba.de/downloads/39-261-5433/2022-05-19_AM-RL-XII_Abemaciclib_D-754_BAnz.pdf. Accessed 29 March 2023

[35]: Gemeinsamer Bundesausschuss (GBA) (2022) Beschluss des Gemeinsamen Bundesausschusses über eine Änderung der Arzneimittel-Richtlinie: Anlage XII - Nutzenbewertung von Arzneimitteln mit neuen Wirkstoffen nach § 35a des Fünften Buches Sozialgesetzbuch (SGB V) Abemaciclib (Neues Anwendungsgebiet: Mammakarzinom, HR+, HER2-, früh mit hohem Rezidivrisiko, adjuvante Therapie, Kombination mit endokriner Therapie). https://www.g-ba.de/downloads/39-261-5662/2022-10-20_AM-RL-XII_Abemaciclib_D-811_BAnz.pdf. Accessed 29 March 2023

[36]: Gemeinsamer Bundesausschuss (GBA) (2019) Beschluss des Gemeinsamen Bundesausschusses über eine Änderung der Arzneimittel-Richtlinie (AM-RL): Anlage XII - Beschlüsse über die Nutzenbewertung von Arzneimitteln mit neuen Wirkstoffen nach § 35a SGB V - Binimetinib. https://www.g-ba.de/downloads/39-261-3725/2019-03-22_AM-RL-XII_Binimetinib_D-388_BAnz.pdf. Accessed 29 March 2023

[37]: Gemeinsamer Bundesausschuss (GBA) (2019) Beschluss des Gemeinsamen Bundesausschusses über eine Änderung der Arzneimittel-Richtlinie (AM-RL): Anlage XII - Beschlüsse über die Nutzenbewertung von Arzneimitteln mit neuen Wirkstoffen nach § 35a SGB V - Encorafenib. https://www.g-ba.de/downloads/39-261-3710/2019-03-22_AM-RL-XII_Encorafenib_D-389_BAnz.pdf. Accessed 29 March 2023

[38]: Gemeinsamer Bundesausschuss (GBA) (2020) Beschluss des Gemeinsamen Bundesausschusses über eine Änderung der Arzneimittel-Richtlinie (AM-RL): Anlage XII - Nutzenbewertung von Arzneimitteln mit neuen Wirkstoffen nach § 35a SGB V - Encorafenib (neues Anwendungsgebiet: metastasiertes Kolorektalkarzinom mit BRAF-V600E-Mutation nach systemischer Vortherapie; in Kombination mit Cetuximab). https://www.g-ba.de/downloads/39-261-4609/2020-12-17_AM-RL-XII_Encorafenib_D-551_BAnz.pdf. Accessed 29 March 2023

[39]: Gemeinsamer Bundesausschuss (GBA) (2019) Beschluss des Gemeinsamen Bundesausschusses über eine Änderung der Arzneimittel-Richtlinie (AM-RL): Anlage XII - Beschlüsse über die Nutzenbewertung von Arzneimitteln mit neuen Wirkstoffen nach § 35a SGB V - Brigatinib. https://www.g-ba.de/downloads/39-261-3859/2019-07-04_AM-RL-XII_Brigatinib_D-434_BAnz.pdf. Accessed 29 March 2023

[40]: Gemeinsamer Bundesausschuss (GBA) (2020) Beschluss des Gemeinsamen Bundesausschusses über eine Änderung der Arzneimittel-Richtlinie (AM-RL): Anlage XII - Nutzenbewertung von Arzneimitteln mit neuen Wirkstoffen nach § 35a SGB V - Brigatinib (neues Anwendungsgebiet: NSCLC, ALK+, ALK-Inhibitor-naive Patienten). https://www.g-ba.de/downloads/39-261-4498/2020-10-15_AM-RL_XII_Brigatinib_D-542_BAnz.pdf. Accessed 29 March 2023

[41]: Gemeinsamer Bundesausschuss (GBA) (2019) Beschluss des Gemeinsamen Bundesausschusses über eine Änderung der Arzneimittel-Richtlinie (AM-RL): Anlage XII - Nutzenbewertung von Arzneimitteln mit neuen Wirkstoffen nach § 35a SGB V - Dacomitinib. https://www.g-ba.de/downloads/39-261-3992/2019-10-17_AM-RL-XII_Dacomitinib_D-442_BAnz.pdf. Accessed 29 March 2023

[42]: Gemeinsamer Bundesausschuss (GBA) (2020) Beschluss des Gemeinsamen Bundesausschusses über eine Änderung der Arzneimittel-Richtlinie (AM-RL): Anlage XII - Nutzenbewertung von Arzneimitteln mit neuen Wirkstoffen nach § 35a SGB V - Gilteritinib (rezidivierte oder refraktäre akute myeloische Leukämie mit FLT3-Mutation). https://www.g-ba.de/downloads/39-261-4287/2020-05-14_AM-RL-XII_Gilteritinib_D-503_BAnz.pdf. Accessed 29 March 2023

[43]: Gemeinsamer Bundesausschuss (GBA) (2020) Beschluss des Gemeinsamen Bundesausschusses über eine Änderung der Arzneimittel-Richtlinie (AM-RL): Anlage XII - Nutzenbewertung von Arzneimitteln mit neuen Wirkstoffen nach § 35a SGB V - Larotrectinib (solide Tumore, Histologie-unabhängig). https://www.g-ba.de/downloads/39-261-4242/2020-04-02_AM-RL-XII_Larotrectinib_D-495_BAnz.pdf. Accessed 29 March 2023

[44]: Gemeinsamer Bundesausschuss (GBA) (2019) Beschluss des Gemeinsamen Bundesausschusses über eine Änderung der Arzneimittel-Richtlinie (AM-RL): Anlage XII - Nutzenbewertung von Arzneimitteln mit neuen Wirkstoffen nach § 35a SGB V - Lorlatinib. https://www.g-ba.de/downloads/39-261-4029/2019-11-22_AM-RL-XII_Lorlatinib_D-451_BAnz.pdf. Accessed 29 March 2023

[45]: Gemeinsamer Bundesausschuss (GBA) (2022) Beschluss des Gemeinsamen Bundesausschusses über eine Änderung der Arzneimittel-Richtlinie: Anlage XII - Nutzenbewertung von Arzneimitteln mit neuen Wirkstoffen nach § 35a des Fünften Buches Sozialgesetzbuch (SGB V) Lorlatinib (neues Anwendungsgebiet: Nicht-kleinzelliges Lungenkarzinom, ALK+, Erstlinie). https://www.g-ba.de/downloads/39-261-5607/2022-09-01_AM-RL-XII_Lorlatinib_D-792_BAnz.pdf. Accessed 29 March 2023

[46]: Gemeinsamer Bundesausschuss (GBA) (2020) Beschluss des Gemeinsamen Bundesausschusses über eine Änderung der Arzneimittel-Richtlinie (AM-RL): Anlage XII - Nutzenbewertung von Arzneimitteln mit neuen Wirkstoffen nach § 35a SGB V - Neratinib (Mammakarzinom, HR-positiv, HER2-positiv, adjuvante Behandlung). https://www.g-ba.de/downloads/39-261-4290/2020-05-14_AM-RL_XII_Neratinib_D-506_BAnz.pdf. Accessed 29 March 2023

[47]: Gemeinsamer Bundesausschuss (GBA) (2021) Beschluss des Gemeinsamen Bundesausschusses über eine Änderung der Arzneimittel-Richtlinie (AM-RL): Anlage XII - Nutzenbewertung von Arzneimitteln mit neuen Wirkstoffen nach § 35a SGB V Acalabrutinib (Chronisch lymphatische Leukämie, Monotherapie, Erstlinie). https://www.g-ba.de/downloads/39-261-4867/2021-06-03_AM-RL-XII_Acalabrutinib_D-592_BAnz.pdf. Accessed 29 March 2023

[48]: Gemeinsamer Bundesausschuss (GBA) (2021) Beschluss des Gemeinsamen Bundesausschusses über eine Änderung der Arzneimittel-Richtlinie (AM-RL): Anlage XII - Nutzenbewertung von Arzneimitteln mit neuen Wirkstoffen nach § 35a SGB V Acalabrutinib (Chronisch lymphatische Leukämie, Kombination mit Obinutuzumab, Erstlinie). https://www.g-ba.de/downloads/39-261-4868/2021-06-03_AM-RL-XII_Acalabrutinib_D-593_BAnz.pdf. Accessed 29 March 2023

[49]: Gemeinsamer Bundesausschuss (GBA) (2021) Beschluss des Gemeinsamen Bundesausschusses über eine Änderung der Arzneimittel-Richtlinie (AM-RL) Anlage XII - Nutzenbewertung von Arzneimitteln mit neuen Wirkstoffen nach § 35a SGB V: Acalabrutinib (chronisch lymphatische Leukämie, nach mindestens 1 Vorbehandlung). https://www.g-ba.de/downloads/39-261-4963/2021-08-05_AM-RL-XII_Acalabrutinib_D-594_BAnz.pdf. Accessed 29 March 2023

[50]: Gemeinsamer Bundesausschuss (GBA) (2021) Beschluss des Gemeinsamen Bundesauschusses über eine Änderung der Arzneimittel-Richtlinie (AM-RL): Anlage XII - Nutzenbewertung von Arzneimitteln mit neue Wirkstoffen nach § 35a SGB V Alpelisib in Kombination mit Fulvestrant (Mammakarzinom mit PIK3CA-Mutation, HR+, HER2-, Kombination mit Fulvestrant). https://www.g-ba.de/downloads/39-261-4706/2021-02-18_AM-RL-XII_Alpelisib_D-574_BAnz.pdf. Accessed 29 March 2023

[51]: Gemeinsamer Bundesausschuss (GBA) (2021) Beschluss des Gemeinsamen Bundesausschusses über eine Änderung der Arzneimittel-Richtlinie (AM-RL): Anlage XII - Nutzenbewertung von Arzneimitteln mit neuen Wirkstoffen nach § 35a SGB V Avapritinib (Gastrointestinale Stromatumoren). https://www.g-ba.de/downloads/39-261-4782/2021-04-15_AM-RL-XII_Avapritinib_D-583_BAnz.pdf. Accessed 29 March 2023

[52]: Gemeinsamer Bundesausschuss (GBA) (2022) Beschluss des Gemeinsamen Bundesausschusses über eine Änderung der Arzneimittel-Richtline: Anlage XII - Nutzenbewertung von Arzneimitteln mit neuen Wirkstoffen nach § 35a des Fünften Buches Sozialgesetzbuch (SGB V) Avapritinib (Neues Anwendungsgebiet: systemische Mastozytose, nach mind. 1 Vortherapie). https://www.g-ba.de/downloads/39-261-5616/2022-09-15_AM-RL-XII_Avapritinib_D-798_BAnz.pdf. Accessed 29 March 2023

[53]: Gemeinsamer Bundesausschuss (GBA) (2021) Beschluss des Gemeinsamen Bundesausschusses über eine Änderung der Arzneimittel-Richtlinie (AM-RL): Anlage XII - Nutzenbewertung von Arzneimitteln mit neuen Wirkstoffen nach § 35a SGB V Entrectinib (solide Tumore; Histologie-unabhängig). https://www.g-ba.de/downloads/39-261-4715/2021-02-18_AM-RL-XII_Entrectinib_D-559_BAnz.pdf. Accessed 29 March 2023

[54]: Gemeinsamer Bundesausschuss (GBA) (2021) Beschluss des Gemeinsamen Bundesausschusses über eine Änderung der Arzneimittel-Richtlinie (AM-RL): Anlage XII - Nutzenbewertung von Arzneimitteln mit neuen Wirkstoffe nach § 35a SGB V Entrectinib (ROS1-positives, fortgeschrittenes nicht kleinzelliges Lungenkarzinom). https://www.g-ba.de/downloads/39-261-4714/2021-02-18_AM-RL-XII_Entrectinib_D-558_BAnz.pdf. Accessed 29 March 2023

[55]: Gemeinsamer Bundesausschuss (GBA) (2021) Beschluss des Gemeinsamen Bundesausschusses über eine Änderung der Arzneimittel-Richtlinie (AM-RL) Anlage XII - Nutzenbewertung von Arzneimitteln mit neuen Wirkstoffen nach § 35a SGB V: Fedratinib (Myelofibrose). https://www.g-ba.de/downloads/39-261-5003/2021-09-02_AM-RL-XII_Fedratinib_D-650_BAnz.pdf. Accessed 29 March 2023

[56]: Gemeinsamer Bundesausschuss (GBA) (2021) Beschluss des Gemeinsamen Bundesauschusses über eine Änderung der Arzneimittel-Richtlinie: Anlage XII - Nutzenbewertung von Arzneimitteln mit neuen Wirkstoffen nach § 35a SGB V Pemigatinib (Cholangiokarzinom mit FGFR2-Fusion oder FGFR2-Rearrangement, nach mindestens 1 Vortherapie). https://www.g-ba.de/downloads/39-261-5049/2021-10-07_AM-RL-XII_Pemigatinib_D-670_BAnz.pdf. Accessed 29 March 2023

[57]: Gemeinsamer Bundesausschuss (GBA) (2021) Beschluss des Gemeinsamen Bundesausschusses über eine Änderung der Arzneimittel-Richtlinie: Anlage XII - Nutzenbewertung von Arzneimitteln mit neuen Wirkstoffen nach § 35a SGB V Selpercatinib (Lungenkarzinom, nicht-kleinzelliges, RET-Fusion-positiv, nach Platin-basierter Chemo- und/oder Immuntherapie). https://www.g-ba.de/downloads/39-261-4998/2021-09-02_AM-RL-XII_Selperacitinib_D-655_BAnz.pdf. Accessed 29 March 2023

[58]: Gemeinsamer Bundesauschuss (GBA) (2021) Beschluss des Gemeinsamen Bundesausschusses über eine Änderung der Arzneimittel-Richtlinie (AM-RL): Anlage XII - Nutzenbewertung von Arzneimitteln mit neuen Wirkstoffen nach § 35a SGB V Selpercatinib (Schilddrüsenkarzinom, RET-mutiert, nach Cabozantinib und/oder Vandetanib Vortherapie, $\geq$ 12 Jahre). https://www.g-ba.de/downloads/39-261-4999/2021-09-02_AM-RL-XII_Selpercatinib_D-656_BAnz.pdf. Accessed 29 March 2023

[59]: Gemeinsamer Bundesausschuss (GBA) (2021) Beschluss des Gemeinsamen Bundesauschusses über eine Änderung der Arzneimittel-Richtlinie (AM-RL): Anlage XII - Nutzenbewertung von Arzneimitteln mit neuen Wirkstoffen nach § 35a SGB V Selpercatinib (Schilddrüsenkarzinom, RET-Fusion+, nach Sorafenib und/oder Lenvatinib Vortherapie). https://www.g-ba.de/downloads/39-261-5001/2021-09-02_AM-RL-XII_Selpercatinib_D-657_BAnz.pdf. Accessed 29 March 2023

[60]: Gemeinsamer Bundesauschuss (GBA) (2023) Beschluss des Gemeinsamen Bundesauschusses über eine Änderung der Arzneimittel-Richtlinie: Anlage XII - Nutzenbewertung von Arzneimitteln mit neuen Wirkstoffen nach § 35a des Fünften Buches Sozialgesetzbuch (SGB V) Selpercatinib (neues Anwendungsgebiet: Lungenkarzinom, nicht-kleinzelliges, RET-Fusion+, Erstlinie). https://www.g-ba.de/downloads/39-261-5770/2022-12-15_AM-RL-XII_Selpercatinib_D-832_BAnz.pdf. Accessed 29 March 2023

[61]: Gemeinsamer Bundesauschuss (GBA) (2023) Beschluss des Gemeinsamen Bundesauschusses über eine Änderung der Arzneimittel-Richtlinie: Anlage XII - Nutzenbewertung von Arzneimitteln mit neuen Wirkstoffen nach § 35a des Fünften Buches Sozialgesetzbuch (SGB V) Selpercatinib (neues Anwendungsgebiet: medulläres Schilddrüsenkarzinom, RET-mutiert, Monotherapie, ab 12 Jahren). https://www.g-ba.de/downloads/39-261-5910/2023-03-16_AM-RL-XII_Selpercatinib_D-874.pdf. Accessed 29 March 2023

[62]: Gemeinsamer Bundesauschuss (GBA) (2022) Beschluss des Gemeinsamen Bundesauschusses über eine Änderung der Arzneimittel-Richtlinie (AM-RL): Anlage XII - Nutzenbewertung von Arzneimitteln mit neuen Wirkstoffen nach § 35a SGB V Selumetinib (Neurofibromatose ($\geq$ 3 bis < 18 Jahre, Typ 1)). https://www.g-ba.de/downloads/39-261-5266/2022-02-03_AM-RL-XII_Selumetinib_D-714_BAnz.pdf. Accessed 29 March 2023

[63]: Gemeinsamer Bundesausschuss (GBA) (2021) Beschluss des Gemeinsamen Bundesausschusses über eine Änderung der Arzneimittel-Richtlinie (AM-RL) Anlage XII - Nutzenbewertung von Arzneimitteln mit neuen Wirkstoffen nach § 35a des Fünften Buches Sozialgesetzbuch (SGB V): Tucatinib (Mammakarzinom, HER2+, mind. 2 Vortherapien, Kombination mit Trastuzumab und Capecitabin). https://www.g-ba.de/downloads/39-261-5000/2021-09-02_AM-RL-XII_Tucatinib_D-654_BAnz.pdf. Accessed 29 March 2023

**References Table S 3:**

[1]: Gemeinsamer Bundesausschuss (GBA) (2017) Beschluss des Gemeinsamen Bundesausschusses über eine Änderung der Arzneimittel-Richtlinie (AM-RL): Anlage XII - Beschlüsse über die Nutzenbewertung von Arzneimitteln mit neuen Wirkstoffen nach § 35a SGB V - Ceritinib (Ablauf der Befristung). https://www.g-ba.de/downloads/39-261-2876/2017-03-16_AM-RL-XII_Ceritinib_D-259_BAnz.pdf. Accessed 29 March 2023

[2]: Gemeinsamer Bundesausschuss (GBA) (2018) Beschluss des Gemeinsamen Bundesausschusses über eine Änderung der Arzneimittel-Richtlinie (AM-RL): Anlage XII - Beschlüsse über die Nutzenbewertung von Arzneimitteln mit neuen Wirkstoffen nach § 35a SGB V - Ceritinib (neues Anwendungsgebiet: Erstlinienbehandlung, nicht-kleinzelliges Lungenkarzinom). https://www.g-ba.de/downloads/39-261-3214/2018-02-01_AM-RL-XII_Ceritinib_nAWG_D-296_BAnz.pdf. Accessed 29 March 2023

[3]: Gemeinsamer Bundesausschuss (GBA) (2016) Beschluss des Gemeinsamen Bundesausschusses über eine Änderung der Arzneimittel-Richtlinie (AM-RL): Anlage XII - Beschlüsse über die Nutzenbewertung von Arzneimitteln mit neuen Wirkstoffen nach § 35a SGB V - Cobimetinib. https://www.g-ba.de/downloads/39-261-2607/2016-06-02_AM-RL-XII_Cobimetinib_D-196_BAnz.pdf. Accessed 29 March 2023

[4]: Gemeinsamer Bundesausschuss (GBA) (2015) Beschluss des Gemeinsamen Bundesausschusses über eine Änderung der Arzneimittel-Richtlinie (AM-RL): Anlage XII - Nutzenbewertung von Arzneimitteln mit neuen Wirkstoffen nach § 35a SGB V Lenvatinib (Bewertung nach Aufhebung des Orphan Drug-Status). https://www.g-ba.de/downloads/39-261-3922/2019-08-15_AM-RL-XII_Lenvatinib_D-428_BAnz.pdf. Accessed 29 March 2023

[5]: Gemeinsamer Bundesausschuss (GBA) (2021) Beschluss des Gemeinsamen Bundesausschusses über eine Änderung der Arzneimittel-Richtlinie (AM-RL): Anlage XII - Nutzenbewertung von Arzneimitteln mit neuen Wirkstoffen nach § 35a SGB V, Lenvatinib (Neubewertung nach Fristablauf: fortgeschrittenes Nierenzellkarzinom, Kombination mit Everolimus). https://www.g-ba.de/downloads/39-261-4908/2021-07-01_AM-RL-XII_Lenvatinib_D-620_BAnz.pdf. Accessed 29 March 2023

[6]: Gemeinsamer Bundesausschuss (GBA) (2019) Beschluss des Gemeinsamen Bundesausschusses über eine Änderung der Arzneimittel-Richtlinie (AM-RL): Anlage XII - Beschlüsse über die Nutzenbewertung von Arzneimitteln mit neuen Wirkstoffen nach § 35a SGB V - Lenvatinib (neues Anwendungsgebiet Hepatozelluläres Karzinom). https://www.g-ba.de/downloads/39-261-3715/2019-03-22_AM-RL-XII_Lenvatinib-nAWG_D-379_BAnz.pdf. Accessed 29 March 2023

[7]: Gemeinsamer Bundesausschuss (GBA) (2022) Beschluss des Gemeinsamen Bundesausschusses über eine Änderung der Arzneimittel-Richtlinie: Anlage XII - Nutzenbewertung von Arzneimitteln mit neuen Wirkstoffen nach § 35a des Fünften Buches Sozialgesetzbuch (SGB V) Lenvatinib (neues Anwendungsgebiet: Endometriumkarzinom, nach vorheriger Platin-basierter Therapie, Kombination mit Pembrolizumab). https://www.g-ba.de/downloads/39-261-5518/2022-07-07_AM-RL-XII_Lenvantinib_D-755_BAnz.pdf. Accessed 29 March 2023

[8]: Gemeinsamer Bundesausschuss (GBA) (2022) Beschluss des Gemeinsamen Bundesausschusses über eine Änderung der Arzneimittel-Richtlinie: Anlage XII - Nutzenbewertung von Arzneimitteln mit neuen Wirkstoffen nach § 35a des Fünften Buches Sozialgesetzbuch (SGB V) Lenvatinib (neues Anwendungsgebiet: fortgeschrittenes Nierenzellkarzinom, Erstlinie, Kombination mit Pembrolizumab). https://www.g-ba.de/downloads/39-261-5521/2022-07-07_AM-RL-XII_Lenvatinib_D-749_BAnz.pdf. Accessed 29 March 2023

[9]: Gemeinsamer Bundesausschuss (GBA) (2015) Beschluss des Gemeinsamen Bundesausschusses über eine Änderung der Arzneimittel-Richtlinie (AM-RL): Anlage XII - Beschlüsse über die Nutzenbewertung von Arzneimitteln mit neuen Wirkstoffen nach § 35a SGB V - Nintedanib. https://www.g-ba.de/downloads/39-261-2262/2015-06-18_AM-RL-XII_Nintedanib_2015-01-01-D-147_BAnz.pdf. Accessed 29 March 2023

[10]: Gemeinsamer Bundesausschuss (GBA) (2016) Beschluss des Gemeinsamen Bundesausschusses über eine Änderung der Arzneimittel-Richtlinie (AM-RL): Anlage XII - Beschlüsse über die Nutzenbewertung von Arzneimitteln mit neuen Wirkstoffen nach § 35a SGB V - Trametinib. https://www.g-ba.de/downloads/39-261-2531/2016-03-17_AM-RL-XII_Trametinib_2015-10-01-D-183_BAnz.pdf. Accessed 29 March 2023

[11]: Gemeinsamer Bundesausschuss (GBA) (2019) Beschluss des Gemeinsamen Bundesausschusses über eine Änderung der Arzneimittel-Richtlinie (AM-RL): Anlage XII - Beschlüsse über die Nutzenbewertung von Arzneimitteln mit neuen Wirkstoffen nach § 35a SGB V - Trametinib (neues Anwendungsgebiet: Melanom, in Kombination mit Dabrafenib, BRAF-V600-Mutation, adjuvante Behandlung). https://www.g-ba.de/downloads/39-261-3720/2019-03-22_AM-RL-XII_Trametinib_D-384_BAnz.pdf. Accessed 29 March 2023

[12]: Gemeinsamer Bundesausschuss (GBA) (2017) Beschluss des Gemeinsamen Bundesausschusses über eine Änderung der Arzneimittel-Richtlinie (AM-RL): Anlage XII - Beschlüsse über die Nutzenbewertung von Arzneimitteln mit neuen Wirkstoffen nach § 35a SGB V - Trametinib (neues Anwendungsgebiet: nicht-kleinzelliges Lungenkarzinom). https://www.g-ba.de/downloads/39-261-3095/2017-10-19_AM-RL-XII_Trametinib_D-284_BAnz.pdf. Accessed 29 March 2023

[13]: Gemeinsamer Bundesausschuss (GBA) (2016) Beschluss des Gemeinsamen Bundesausschusses über eine Änderung der Arzneimittel-Richtlinie (AM-RL): Anlage XII - Beschlüsse über die Nutzenbewertung von Arzneimitteln mit neuen Wirkstoffen nach § 35a SGB V - Osimertinib. https://www.g-ba.de/downloads/39-261-2700/2016-09-15_AM-RL-XII_Osimertinib_D-219_BAnz.pdf. Accessed 29 March 2023

[14]: Gemeinsamer Bundesausschuss (GBA) (2019) Beschluss des Gemeinsamen Bundesausschusses über eine Änderung der Arzneimittel-Richtlinie (AM-RL): Anlage XII - Beschlüsse über die Nutzenbewertung von Arzneimitteln mit neuen Wirkstoffen nach § 35a SGB V - Osimertinib (neues Anwendungsgebiet: Erstlinientherapie des lokal fortgeschrittenen oder metastasierten nicht-kleinzelligen Lungenkarzinoms). https://www.g-ba.de/downloads/39-261-3646/2019-01-17_AM-RL-XII_Osimertinib_D-369_BAnz.pdf. Accessed 29 March 2023

[15]: Gemeinsamer Bundesausschuss (GBA) (2021) Beschluss des Gemeinsamen Bundesausschusses über eine Änderung der Arzneimittel-Richtlinie (AM-RL): Anlage XII - Beschlüsse über die Nutzenbewertung von Arzneimitteln mit neuen Wirkstoffen nach § 35a SGB V - Osimertinib (neues Anwendungsgebiet: Nicht-kleinzelliges Lungenkarzinom, EGFR Mutation, adjuvante Therapie). https://www.g-ba.de/downloads/39-261-5177/2021-12-16_AM-RL-XII_Osimertinib_D-701.pdf. Accessed 29 March 2023

[16]: Gemeinsamer Bundesausschuss (GBA) (2019) Beschluss des Gemeinsamen Bundesausschusses über eine Änderung der Arzneimittel-Richtlinie (AM-RL): Anlage XII - Beschlüsse über die Nutzenbewertung von Arzneimitteln mit neuen Wirkstoffen nach § 35a SGB V - Palbociclib (Brustkrebs; in Kombination mit Fulvestrant nach endokriner Therapie; Neubewertung nach Fristablauf). https://www.g-ba.de/downloads/39-261-3713/2019-03-22_AM-RL-XII_Palbociclib_D-395_BAnz.pdf. Accessed 29 March 2023

[17]: Gemeinsamer Bundesausschuss (GBA) (2022) Beschluss des Gemeinsamen Bundesausschusses über eine Änderung der Arzneimittel-Richtlinie: Anlage XII - Nutzenbewertung von Arzneimitteln mit neuen Wirkstoffen nach § 35a des Fünften Buches Sozialgesetzbuch (SGB V) Palbociclib (Neubewertung nach Fristablauf: Mammakarzinom, HR+, HER2-, Kombination mit Aromatasehemmern). https://www.g-ba.de/downloads/39-261-5773/2022-12-15_AM-RL-XII_Palbociclib_D-834_BAnz.pdf. Accessed 29 March 2023

[18]: Gemeinsamer Bundesausschuss (GBA) (2017) Beschluss des Gemeinsamen Bundesausschusses über eine Änderung der Arzneimittel-Richtlinie (AM-RL): Anlage XII - Beschlüsse über die Nutzenbewertung von Arzneimitteln mit neuen Wirkstoffen nach § 35a SGB V - Alectinib. https://www.g-ba.de/downloads/39-261-3090/2017-10-19_AM-RL-XII_Alectinib_D-281_BAnz.pdf. Accessed 29 March 2023

[19]: Gemeinsamer Bundesausschuss (GBA) (2018) Beschluss des Gemeinsamen Bundesausschusses über eine Änderung der Arzneimittel-Richtlinie (AM-RL): Anlage XII - Beschlüsse über die Nutzenbewertung von Arzneimitteln mit neuen Wirkstoffen nach § 35a SGB V - Alectinib (neues Anwendungsgebiet: Erstlinienbehandlung nicht-kleinzelliges Lungenkarzinom). https://www.g-ba.de/downloads/39-261-3368/2018-06-21_AM-RL-XII_Alectinib_D-326_BAnz.pdf. Accessed 29 March 2023

[20]: Gemeinsamer Bundesausschuss (GBA) (2018) Beschluss des Gemeinsamen Bundesausschusses über eine Änderung der Arzneimittel-Richtlinie (AM-RL): Anlage XII - Beschlüsse über die Nutzenbewertung von Arzneimitteln mit neuen Wirkstoffen nach § 35a SGB V - Midostaurin (akute myeloische Leukämie). https://www.g-ba.de/downloads/39-261-3277/2018-04-05_AM-RL-XII_Midostaurin_AML_D-319_BAnz.pdf. Accessed 29 March 2023

[21]: Gemeinsamer Bundesausschuss (GBA) (2018) Beschluss des Gemeinsamen Bundesausschusses über eine Änderung der Arzneimittel-Richtlinie (AM-RL): Anlage XII - Beschlüsse über die Nutzenbewertung von Arzneimitteln mit neuen Wirkstoffen nach § 35a SGB V - Midostaurin (aggressive systemische Mastozytose). https://www.g-ba.de/downloads/39-261-3279/2018-04-05_AM-RL-XII_Midostaurin_ASM_D-319_BAnz.pdf. Accessed 29 March 2023

[22]: Gemeinsamer Bundesausschuss (GBA) (2020) Beschluss des Gemeinsamen Bundesausschusses über eine Änderung der Arzneimittel-Richtlinie (AM-RL): Anlage XII - Nutzenbewertung von Arzneimitteln mit neuen Wirkstoffen nach § 35a SGB V - Ribociclib (Neubewertung nach Fristablauf (Mammakarzinom, HR+, HER2-, Kombination mit einem Aromatasehemmer)). https://www.g-ba.de/downloads/39-261-4423/2020-08-20_AM-RL-XII_Ribociclib_D-517_BAnz.pdf. Accessed 29 March 2023

[23]: Gemeinsamer Bundesausschuss (GBA) (2020) Beschluss des Gemeinsamen Bundesausschusses über eine Änderung der Arzneimittel-Richtlinie (AM-RL): Anlage XII - Nutzenbewertung von Arzneimitteln mit neuen Wirkstoffen nach § 35a SGB V - Ribociclib (Neubewertung nach Fristablauf (Mammakarzinom, HR+, HER2-, Kombination mit Fulvestrant)). https://www.g-ba.de/downloads/39-261-4428/2020-08-20_AM-RL-XII_Ribociclib_D-518_BAnz.pdf. Accessed 29 March 2023

[24]: Gemeinsamer Bundesausschuss (GBA) (2018) Beschluss des Gemeinsamen Bundesausschusses über eine Änderung der Arzneimittel-Richtlinie (AM-RL): Anlage XII - Beschlüsse über die Nutzenbewertung von Arzneimitteln mit neuen Wirkstoffen nach § 35a SGB V - Tivozanib. https://www.g-ba.de/downloads/39-261-3291/2018-04-19_AM-RL-XII_Tivozanib_D-323_BAnz.pdf. Accessed 29 March 2023

[25]: Gemeinsamer Bundesausschuss (GBA) (2019) Beschluss des Gemeinsamen Bundesausschusses über eine Änderung der Arzneimittel-Richtlinie (AM-RL): Anlage XII - Beschlüsse über die Nutzenbewertung von Arzneimitteln mit neuen Wirkstoffen nach § 35a SGB V - Abemaciclib (Brustkrebs; in Kombination mit einem Aromatasehemmer). https://www.g-ba.de/downloads/39-261-3767/2019-05-02_AM-RL-XII_Abemaciclib_D-400_BAnz.pdf. Accessed 29 March 2023

[26]: Gemeinsamer Bundesausschuss (GBA) (2022) Beschluss des Gemeinsamen Bundesausschusses über eine Änderung der Arzneimittel-Richtlinie: Anlage XII - Nutzenbewertung von Arzneimitteln mit neuen Wirkstoffen nach § 35a des Fünften Buches Sozialgesetzbuch (SGB V) Abemaciclib (Neubewertung nach Fristablauf: Mammakarzinom, HR+, HER2-, Kombination mit Fulvestrant). https://www.g-ba.de/downloads/39-261-5433/2022-05-19_AM-RL-XII_Abemaciclib_D-754_BAnz.pdf. Accessed 29 March 2023

[27]: Gemeinsamer Bundesausschuss (GBA) (2022) Beschluss des Gemeinsamen Bundesausschusses über eine Änderung der Arzneimittel-Richtlinie: Anlage XII - Nutzenbewertung von Arzneimitteln mit neuen Wirkstoffen nach § 35a des Fünften Buches Sozialgesetzbuch (SGB V) Abemaciclib (Neues Anwendungsgebiet: Mammakarzinom, HR+, HER2-, früh mit hohem Rezidivrisiko, adjuvante Therapie, Kombination mit endokriner Therapie). https://www.g-ba.de/downloads/39-261-5662/2022-10-20_AM-RL-XII_Abemaciclib_D-811_BAnz.pdf. Accessed 29 March 2023

[28]: Gemeinsamer Bundesausschuss (GBA) (2019) Beschluss des Gemeinsamen Bundesausschusses über eine Änderung der Arzneimittel-Richtlinie (AM-RL): Anlage XII - Beschlüsse über die Nutzenbewertung von Arzneimitteln mit neuen Wirkstoffen nach § 35a SGB V - Binimetinib. https://www.g-ba.de/downloads/39-261-3725/2019-03-22_AM-RL-XII_Binimetinib_D-388_BAnz.pdf. Accessed 29 March 2023

[29]: Gemeinsamer Bundesausschuss (GBA) (2019) Beschluss des Gemeinsamen Bundesausschusses über eine Änderung der Arzneimittel-Richtlinie (AM-RL): Anlage XII - Beschlüsse über die Nutzenbewertung von Arzneimitteln mit neuen Wirkstoffen nach § 35a SGB V - Encorafenib. https://www.g-ba.de/downloads/39-261-3710/2019-03-22_AM-RL-XII_Encorafenib_D-389_BAnz.pdf. Accessed 29 March 2023

[30]: Gemeinsamer Bundesausschuss (GBA) (2020) Beschluss des Gemeinsamen Bundesausschusses über eine Änderung der Arzneimittel-Richtlinie (AM-RL): Anlage XII - Nutzenbewertung von Arzneimitteln mit neuen Wirkstoffen nach § 35a SGB V - Encorafenib (neues Anwendungsgebiet: metastasiertes Kolorektalkarzinom mit BRAF-V600E-Mutation nach systemischer Vortherapie; in Kombination mit Cetuximab). https://www.g-ba.de/downloads/39-261-4609/2020-12-17_AM-RL-XII_Encorafenib_D-551_BAnz.pdf. Accessed 29 March 2023

[31]: Gemeinsamer Bundesausschuss (GBA) (2019) Beschluss des Gemeinsamen Bundesausschusses über eine Änderung der Arzneimittel-Richtlinie (AM-RL): Anlage XII - Beschlüsse über die Nutzenbewertung von Arzneimitteln mit neuen Wirkstoffen nach § 35a SGB V - Brigatinib. https://www.g-ba.de/downloads/39-261-3859/2019-07-04_AM-RL-XII_Brigatinib_D-434_BAnz.pdf. Accessed 29 March 2023

[32]: Gemeinsamer Bundesausschuss (GBA) (2020) Beschluss des Gemeinsamen Bundesausschusses über eine Änderung der Arzneimittel-Richtlinie (AM-RL): Anlage XII - Nutzenbewertung von Arzneimitteln mit neuen Wirkstoffen nach § 35a SGB V - Brigatinib (neues Anwendungsgebiet: NSCLC, ALK+, ALK-Inhibitor-naive Patienten). https://www.g-ba.de/downloads/39-261-4498/2020-10-15_AM-RL_XII_Brigatinib_D-542_BAnz.pdf. Accessed 29 March 2023

[33]: Gemeinsamer Bundesausschuss (GBA) (2019) Beschluss des Gemeinsamen Bundesausschusses über eine Änderung der Arzneimittel-Richtlinie (AM-RL): Anlage XII - Nutzenbewertung von Arzneimitteln mit neuen Wirkstoffen nach § 35a SGB V - Dacomitinib. https://www.g-ba.de/downloads/39-261-3992/2019-10-17_AM-RL-XII_Dacomitinib_D-442_BAnz.pdf. Accessed 29 March 2023

[34]: Gemeinsamer Bundesausschuss (GBA) (2020) Beschluss des Gemeinsamen Bundesausschusses über eine Änderung der Arzneimittel-Richtlinie (AM-RL): Anlage XII - Nutzenbewertung von Arzneimitteln mit neuen Wirkstoffen nach § 35a SGB V - Gilteritinib (rezidivierte oder refraktäre akute myeloische Leukämie mit FLT3-Mutation). https://www.g-ba.de/downloads/39-261-4287/2020-05-14_AM-RL-XII_Gilteritinib_D-503_BAnz.pdf. Accessed 29 March 2023

[35]: Gemeinsamer Bundesausschuss (GBA) (2020) Beschluss des Gemeinsamen Bundesausschusses über eine Änderung der Arzneimittel-Richtlinie (AM-RL): Anlage XII - Nutzenbewertung von Arzneimitteln mit neuen Wirkstoffen nach § 35a SGB V - Larotrectinib (solide Tumore, Histologie-unabhängig). https://www.g-ba.de/downloads/39-261-4242/2020-04-02_AM-RL-XII_Larotrectinib_D-495_BAnz.pdf. Accessed 29 March 2023

[36]: Gemeinsamer Bundesausschuss (GBA) (2019) Beschluss des Gemeinsamen Bundesausschusses über eine Änderung der Arzneimittel-Richtlinie (AM-RL): Anlage XII - Nutzenbewertung von Arzneimitteln mit neuen Wirkstoffen nach § 35a SGB V - Lorlatinib. https://www.g-ba.de/downloads/39-261-4029/2019-11-22_AM-RL-XII_Lorlatinib_D-451_BAnz.pdf. Accessed 29 March 2023

[37]: Gemeinsamer Bundesausschuss (GBA) (2022) Beschluss des Gemeinsamen Bundesausschusses über eine Änderung der Arzneimittel-Richtlinie: Anlage XII - Nutzenbewertung von Arzneimitteln mit neuen Wirkstoffen nach § 35a des Fünften Buches Sozialgesetzbuch (SGB V) Lorlatinib (neues Anwendungsgebiet: Nicht-kleinzelliges Lungenkarzinom, ALK+, Erstlinie). https://www.g-ba.de/downloads/39-261-5607/2022-09-01_AM-RL-XII_Lorlatinib_D-792_BAnz.pdf. Accessed 29 March 2023

[38]: Gemeinsamer Bundesausschuss (GBA) (2020) Beschluss des Gemeinsamen Bundesausschusses über eine Änderung der Arzneimittel-Richtlinie (AM-RL): Anlage XII - Nutzenbewertung von Arzneimitteln mit neuen Wirkstoffen nach § 35a SGB V - Neratinib (Mammakarzinom, HR-positiv, HER2-positiv, adjuvante Behandlung). https://www.g-ba.de/downloads/39-261-4290/2020-05-14_AM-RL_XII_Neratinib_D-506_BAnz.pdf. Accessed 29 March 2023

[39]: Gemeinsamer Bundesausschuss (GBA) (2021) Beschluss des Gemeinsamen Bundesausschusses über eine Änderung der Arzneimittel-Richtlinie (AM-RL): Anlage XII - Nutzenbewertung von Arzneimitteln mit neuen Wirkstoffen nach § 35a SGB V Acalabrutinib (Chronisch lymphatische Leukämie, Monotherapie, Erstlinie). https://www.g-ba.de/downloads/39-261-4867/2021-06-03_AM-RL-XII_Acalabrutinib_D-592_BAnz.pdf. Accessed 29 March 2023

[40]: Gemeinsamer Bundesausschuss (GBA) (2021) Beschluss des Gemeinsamen Bundesausschusses über eine Änderung der Arzneimittel-Richtlinie (AM-RL): Anlage XII - Nutzenbewertung von Arzneimitteln mit neuen Wirkstoffen nach § 35a SGB V Acalabrutinib (Chronisch lymphatische Leukämie, Kombination mit Obinutuzumab, Erstlinie). https://www.g-ba.de/downloads/39-261-4868/2021-06-03_AM-RL-XII_Acalabrutinib_D-593_BAnz.pdf. Accessed 29 March 2023

[41]: Gemeinsamer Bundesausschuss (GBA) (2021) Beschluss des Gemeinsamen Bundesausschusses über eine Änderung der Arzneimittel-Richtlinie (AM-RL) Anlage XII - Nutzenbewertung von Arzneimitteln mit neuen Wirkstoffen nach § 35a SGB V: Acalabrutinib (chronisch lymphatische Leukämie, nach mindestens 1 Vorbehandlung). https://www.g-ba.de/downloads/39-261-4963/2021-08-05_AM-RL-XII_Acalabrutinib_D-594_BAnz.pdf. Accessed 29 March 2023

[42]: Gemeinsamer Bundesausschuss (GBA) (2021) Beschluss des Gemeinsamen Bundesauschusses über eine Änderung der Arzneimittel-Richtlinie (AM-RL): Anlage XII - Nutzenbewertung von Arzneimitteln mit neue Wirkstoffen nach § 35a SGB V Alpelisib in Kombination mit Fulvestrant (Mammakarzinom mit PIK3CA-Mutation, HR+, HER2-, Kombination mit Fulvestrant). https://www.g-ba.de/downloads/39-261-4706/2021-02-18_AM-RL-XII_Alpelisib_D-574_BAnz.pdf. Accessed 29 March 2023

[43]: Gemeinsamer Bundesausschuss (GBA) (2021) Beschluss des Gemeinsamen Bundesausschusses über eine Änderung der Arzneimittel-Richtlinie (AM-RL): Anlage XII - Nutzenbewertung von Arzneimitteln mit neuen Wirkstoffen nach § 35a SGB V Avapritinib (Gastrointestinale Stromatumoren). https://www.g-ba.de/downloads/39-261-4782/2021-04-15_AM-RL-XII_Avapritinib_D-583_BAnz.pdf. Accessed 29 March 2023

[44]: Gemeinsamer Bundesausschuss (GBA) (2022) Beschluss des Gemeinsamen Bundesausschusses über eine Änderung der Arzneimittel-Richtline: Anlage XII - Nutzenbewertung von Arzneimitteln mit neuen Wirkstoffen nach § 35a des Fünften Buches Sozialgesetzbuch (SGB V) Avapritinib (Neues Anwendungsgebiet: systemische Mastozytose, nach mind. 1 Vortherapie). https://www.g-ba.de/downloads/39-261-5616/2022-09-15_AM-RL-XII_Avapritinib_D-798_BAnz.pdf. Accessed 29 March 2023

[45]: Gemeinsamer Bundesausschuss (GBA) (2021) Beschluss des Gemeinsamen Bundesausschusses über eine Änderung der Arzneimittel-Richtlinie (AM-RL): Anlage XII - Nutzenbewertung von Arzneimitteln mit neuen Wirkstoffen nach § 35a SGB V Entrectinib (solide Tumore; Histologie-unabhängig). https://www.g-ba.de/downloads/39-261-4715/2021-02-18_AM-RL-XII_Entrectinib_D-559_BAnz.pdf. Accessed 29 March 2023

[46]: Gemeinsamer Bundesausschuss (GBA) (2021) Beschluss des Gemeinsamen Bundesausschusses über eine Änderung der Arzneimittel-Richtlinie (AM-RL): Anlage XII - Nutzenbewertung von Arzneimitteln mit neuen Wirkstoffe nach § 35a SGB V Entrectinib (ROS1-positives, fortgeschrittenes nicht kleinzelliges Lungenkarzinom). https://www.g-ba.de/downloads/39-261-4714/2021-02-18_AM-RL-XII_Entrectinib_D-558_BAnz.pdf. Accessed 29 March 2023

[47]: Gemeinsamer Bundesausschuss (GBA) (2021) Beschluss des Gemeinsamen Bundesausschusses über eine Änderung der Arzneimittel-Richtlinie (AM-RL) Anlage XII - Nutzenbewertung von Arzneimitteln mit neuen Wirkstoffen nach § 35a SGB V: Fedratinib (Myelofibrose). https://www.g-ba.de/downloads/39-261-5003/2021-09-02_AM-RL-XII_Fedratinib_D-650_BAnz.pdf. Accessed 29 March 2023

[48]: Gemeinsamer Bundesausschuss (GBA) (2021) Beschluss des Gemeinsamen Bundesauschusses über eine Änderung der Arzneimittel-Richtlinie: Anlage XII - Nutzenbewertung von Arzneimitteln mit neuen Wirkstoffen nach § 35a SGB V Pemigatinib (Cholangiokarzinom mit FGFR2-Fusion oder FGFR2-Rearrangement, nach mindestens 1 Vortherapie). https://www.g-ba.de/downloads/39-261-5049/2021-10-07_AM-RL-XII_Pemigatinib_D-670_BAnz.pdf. Accessed 29 March 2023

[49]: Gemeinsamer Bundesausschuss (GBA) (2021) Beschluss des Gemeinsamen Bundesausschusses über eine Änderung der Arzneimittel-Richtlinie: Anlage XII - Nutzenbewertung von Arzneimitteln mit neuen Wirkstoffen nach § 35a SGB V Selpercatinib (Lungenkarzinom, nicht-kleinzelliges, RET-Fusion-positiv, nach Platin-basierter Chemo- und/oder Immuntherapie). https://www.g-ba.de/downloads/39-261-4998/2021-09-02_AM-RL-XII_Selperacitinib_D-655_BAnz.pdf. Accessed 29 March 2023

[50]: Gemeinsamer Bundesauschuss (GBA) (2021) Beschluss des Gemeinsamen Bundesausschusses über eine Änderung der Arzneimittel-Richtlinie (AM-RL): Anlage XII - Nutzenbewertung von Arzneimitteln mit neuen Wirkstoffen nach § 35a SGB V Selpercatinib (Schilddrüsenkarzinom, RET-mutiert, nach Cabozantinib und/oder Vandetanib Vortherapie, $\geq$ 12 Jahre). https://www.g-ba.de/downloads/39-261-4999/2021-09-02_AM-RL-XII_Selpercatinib_D-656_BAnz.pdf. Accessed 29 March 2023

[51]: Gemeinsamer Bundesausschuss (GBA) (2021) Beschluss des Gemeinsamen Bundesauschusses über eine Änderung der Arzneimittel-Richtlinie (AM-RL): Anlage XII - Nutzenbewertung von Arzneimitteln mit neuen Wirkstoffen nach § 35a SGB V Selpercatinib (Schilddrüsenkarzinom, RET-Fusion+, nach Sorafenib und/oder Lenvatinib Vortherapie). https://www.g-ba.de/downloads/39-261-5001/2021-09-02_AM-RL-XII_Selpercatinib_D-657_BAnz.pdf. Accessed 29 March 2023

[52]: Gemeinsamer Bundesauschuss (GBA) (2023) Beschluss des Gemeinsamen Bundesauschusses über eine Änderung der Arzneimittel-Richtlinie: Anlage XII - Nutzenbewertung von Arzneimitteln mit neuen Wirkstoffen nach § 35a des Fünften Buches Sozialgesetzbuch (SGB V) Selpercatinib (neues Anwendungsgebiet: Lungenkarzinom, nicht-kleinzelliges, RET-Fusion+, Erstlinie). https://www.g-ba.de/downloads/39-261-5770/2022-12-15_AM-RL-XII_Selpercatinib_D-832_BAnz.pdf. Accessed 29 March 2023

[53]: Gemeinsamer Bundesauschuss (GBA) (2023) Beschluss des Gemeinsamen Bundesauschusses über eine Änderung der Arzneimittel-Richtlinie: Anlage XII - Nutzenbewertung von Arzneimitteln mit neuen Wirkstoffen nach § 35a des Fünften Buches Sozialgesetzbuch (SGB V) Selpercatinib (neues Anwendungsgebiet: medulläres Schilddrüsenkarzinom, RET-mutiert, Monotherapie, ab 12 Jahren). https://www.g-ba.de/downloads/39-261-5910/2023-03-16_AM-RL-XII_Selpercatinib_D-874.pdf. Accessed 29 March 2023

[54]: Gemeinsamer Bundesauschuss (GBA) (2022) Beschluss des Gemeinsamen Bundesauschusses über eine Änderung der Arzneimittel-Richtlinie (AM-RL): Anlage XII - Nutzenbewertung von Arzneimitteln mit neuen Wirkstoffen nach § 35a SGB V Selumetinib (Neurofibromatose ($\geq$ 3 bis < 18 Jahre, Typ 1)). https://www.g-ba.de/downloads/39-261-5266/2022-02-03_AM-RL-XII_Selumetinib_D-714_BAnz.pdf. Accessed 29 March 2023

[55]: Gemeinsamer Bundesausschuss (GBA) (2021) Beschluss des Gemeinsamen Bundesausschusses über eine Änderung der Arzneimittel-Richtlinie (AM-RL) Anlage XII - Nutzenbewertung von Arzneimitteln mit neuen Wirkstoffen nach § 35a des Fünften Buches Sozialgesetzbuch (SGB V): Tucatinib (Mammakarzinom, HER2+, mind. 2 Vortherapien, Kombination mit Trastuzumab und Capecitabin). https://www.g-ba.de/downloads/39-261-5000/2021-09-02_AM-RL-XII_Tucatinib_D-654_BAnz.pdf. Accessed 29 March 2023

[56]: European Society for Medical Oncology (ESMO) (2023) ESMO-MCBS Scorecards: Ceritinib ASCEND-5. https://www.esmo.org/guidelines/esmo-mcbs/esmo-mcbs-for-solid-tumours/esmo-mcbs-scorecards/scorecard-127-1. Accessed 12 January 2024

[57]: European Society for Medical Oncology (ESMO) (2023) ESMO-MCBS Scorecards: Ceritinib ASCEND-4. https://www.esmo.org/guidelines/esmo-mcbs/esmo-mcbs-for-solid-tumours/esmo-mcbs-scorecards/scorecard-128-1. Accessed 12 January 2024

[58]: European Society for Medical Oncology (ESMO) (2023) ESMO-MCBS Scorecards: Cobimetinib coBRIM. https://www.esmo.org/guidelines/esmo-mcbs/esmo-mcbs-for-solid-tumours/esmo-mcbs-scorecards/scorecard-88-1. Accessed 12 January 2024

[59]: European Society for Medical Oncology (ESMO) (2023) ESMO-MCBS Scorecards: Lenvatinib SELECT. https://www.esmo.org/guidelines/esmo-mcbs/esmo-mcbs-for-solid-tumours/esmo-mcbs-scorecards/scorecard-135-1. Accessed 12 January 2024

[60]: European Society for Medical Oncology (ESMO) (2023) ESMO-MCBS Scorecards: Lenvatinib. https://www.esmo.org/guidelines/esmo-mcbs/esmo-mcbs-for-solid-tumours/esmo-mcbs-scorecards/scorecard-112-1. Accessed 12 January 2024

[61]: European Society for Medical Oncology (ESMO) (2023) ESMO-MCBS Scorecards: Lenvatinib KEYNOTE-775. https://www.esmo.org/guidelines/esmo-mcbs/esmo-mcbs-for-solid-tumours/esmo-mcbs-scorecards/scorecard-336-1. Accessed 12 January 2024

[62]: European Society for Medical Oncology (ESMO) (2023) ESMO-MCBS Scorecards: Lenvatinib CLEAR. https://www.esmo.org/guidelines/esmo-mcbs/esmo-mcbs-for-solid-tumours/esmo-mcbs-scorecards/scorecard-284-1. Accessed 12 January 2024

[63]: European Society for Medical Oncology (ESMO) (2023) ESMO-MCBS Scorecards: Dab/Tram (dabrafenib and trametinib) COMBI-v. https://www.esmo.org/guidelines/esmo-mcbs/esmo-mcbs-for-solid-tumours/esmo-mcbs-scorecards/scorecard-87-1. Accessed 12 January 2024

[64]: European Society for Medical Oncology (ESMO) (2023) ESMO-MCBS Scorecards: Dab/Tram (dabrafenib and trametinib) COMBI-AD. https://www.esmo.org/guidelines/esmo-mcbs/esmo-mcbs-for-solid-tumours/esmo-mcbs-scorecards/scorecard-172-1. Accessed 12 January 2024

[65]: European Society for Medical Oncology (ESMO) (2023) ESMO-MCBS Scorecards: Dab/Tram (dabrafenib and trametinib). https://www.esmo.org/guidelines/esmo-mcbs/esmo-mcbs-for-solid-tumours/esmo-mcbs-scorecards/scorecard-130-1. Accessed 12 January 2024

[66]: European Society for Medical Oncology (ESMO) (2023) ESMO-MCBS Scorecards: Osimertinib AURA3. https://www.esmo.org/guidelines/esmo-mcbs/esmo-mcbs-for-solid-tumours/esmo-mcbs-scorecards/scorecard-62-1. Accessed 12 January 2024

[67]: European Society for Medical Oncology (ESMO) (2023) ESMO-MCBS Scorecards: Osimertinib FLAURA. https://www.esmo.org/guidelines/esmo-mcbs/esmo-mcbs-for-solid-tumours/esmo-mcbs-scorecards/scorecard-123-1. Accessed 12 January 2024

[68|: European Society for Medical Oncology (ESMO) (2023) ESMO-MCBS Scorecards: Osimertinib ADAURA. https://www.esmo.org/guidelines/esmo-mcbs/esmo-mcbs-for-solid-tumours/esmo-mcbs-scorecards/scorecard-241-1. Accessed 12 January 2024

[69]: European Society for Medical Oncology (ESMO) (2023) ESMO-MCBS Scorecards: Palbociclib PALOMA-2. https://www.esmo.org/guidelines/esmo-mcbs/esmo-mcbs-for-solid-tumours/esmo-mcbs-scorecards/scorecard-7-1. Accessed 12 January 2024

[70]: European Society for Medical Oncology (ESMO) (2023) ESMO-MCBS Scorecards: Alectinib ALUR. https://www.esmo.org/guidelines/esmo-mcbs/esmo-mcbs-for-solid-tumours/esmo-mcbs-scorecards/scorecard-125-1. Accessed 12 January 2024

[71]: European Society for Medical Oncology (ESMO) (2023) ESMO-MCBS Scorecards: Alectinib ALEX. https://www.esmo.org/guidelines/esmo-mcbs/esmo-mcbs-for-solid-tumours/esmo-mcbs-scorecards/scorecard-124-1. Accessed 12 January 2024

[72]: European Society for Medical Oncology (ESMO) (2023) ESMO-MCBS Scorecards: Ribociclib MONALEESA-2. https://www.esmo.org/guidelines/esmo-mcbs/esmo-mcbs-for-solid-tumours/esmo-mcbs-scorecards/scorecard-9-1. Accessed 12 January 2024

[73]: European Society for Medical Oncology (ESMO) (2023) ESMO-MCBS Scorecards: Ribociclib MONALEESA-3. https://www.esmo.org/guidelines/esmo-mcbs/esmo-mcbs-for-solid-tumours/esmo-mcbs-scorecards/scorecard-161-1. Accessed 12 January 2024

[74]: European Society for Medical Oncology (ESMO) (2023) ESMO-MCBS Scorecards: Tivozanib TIVO-3. https://www.esmo.org/guidelines/esmo-mcbs/esmo-mcbs-for-solid-tumours/esmo-mcbs-scorecards/scorecard-268-1. Accessed 12 January 2024

[75]: European Society for Medical Oncology (ESMO) (2023) ESMO-MCBS Scorecards: Abemaciclib MONARCH 3. https://www.esmo.org/guidelines/esmo-mcbs/esmo-mcbs-for-solid-tumours/esmo-mcbs-scorecards/scorecard-159-1. Accessed 12 January 2024

[76]: European Society for Medical Oncology (ESMO) (2023) ESMO-MCBS Scorecards: Abemaciclib MONARCH 2. https://www.esmo.org/guidelines/esmo-mcbs/esmo-mcbs-for-solid-tumours/esmo-mcbs-scorecards/scorecard-160-1. Accessed 12 January 2024

[77]: European Society for Medical Oncology (ESMO) (2023) ESMO-MCBS Scorecards: Abemaciclib monarchE. https://www.esmo.org/guidelines/esmo-mcbs/esmo-mcbs-for-solid-tumours/esmo-mcbs-scorecards/scorecard-307-1. Accessed 12 January 2024

[78]: European Society for Medical Oncology (ESMO) (2023) ESMO-MCBS Scorecards: Binimetinib COLUMBUS. https://www.esmo.org/guidelines/esmo-mcbs/esmo-mcbs-for-solid-tumours/esmo-mcbs-scorecards/scorecard-168-1. Accessed 12 January 2024

[79]: European Society for Medical Oncology (ESMO) (2023) ESMO-MCBS Scorecards: Encorafenib BEACON CRC. https://www.esmo.org/guidelines/esmo-mcbs/esmo-mcbs-for-solid-tumours/esmo-mcbs-scorecards/scorecard-219-1. Accessed 12 January 2024

[80]: European Society for Medical Oncology (ESMO) (2023) ESMO-MCBS Scorecards: Brigatinib ALTA. https://www.esmo.org/guidelines/esmo-mcbs/esmo-mcbs-for-solid-tumours/esmo-mcbs-scorecards/scorecard-153-1. Accessed 12 January 2024

[81]: European Society for Medical Oncology (ESMO) (2023) ESMO-MCBS Scorecards: Brigatinib ALTA-1L. https://www.esmo.org/guidelines/esmo-mcbs/esmo-mcbs-for-solid-tumours/esmo-mcbs-scorecards/scorecard-213-1. Accessed 12 January 2024

[82]: European Society for Medical Oncology (ESMO) (2023) ESMO-MCBS Scorecards: Dacomitinib ARCHER 1050. https://www.esmo.org/guidelines/esmo-mcbs/esmo-mcbs-for-solid-tumours/esmo-mcbs-scorecards/scorecard-141-1. Accessed 12 January 2024

[83|: European Society for Medical Oncology (ESMO) (2023) ESMO-MCBS Scorecards: Larotrectinib SCOUT and NAVIGATE. https://www.esmo.org/guidelines/esmo-mcbs/esmo-mcbs-for-solid-tumours/esmo-mcbs-scorecards/scorecard-143-1. Accessed 12 January 2024

[84]: European Society for Medical Oncology (ESMO) (2023) ESMO-MCBS Scorecards: Lorlatinib B7461001. https://www.esmo.org/guidelines/esmo-mcbs/esmo-mcbs-for-solid-tumours/esmo-mcbs-scorecards/scorecard-142-1. Accessed 12 January 2024

[85]: European Society for Medical Oncology (ESMO) (2023) ESMO-MCBS Scorecards: Lorlatinib CROWN. https://www.esmo.org/guidelines/esmo-mcbs/esmo-mcbs-for-solid-tumours/esmo-mcbs-scorecards/scorecard-270-1. Accessed 12 January 2024

[86]: European Society for Medical Oncology (ESMO) (2023) ESMO-MCBS Scorecards: Neratinib ExteNET. https://www.esmo.org/guidelines/esmo-mcbs/esmo-mcbs-for-solid-tumours/esmo-mcbs-scorecards/scorecard-134-1. Accessed 12 January 2024

[87]: European Society for Medical Oncology (ESMO) (2023) ESMO-MCBS Scorecards: Alpelisib SOLAR-1. https://www.esmo.org/guidelines/esmo-mcbs/esmo-mcbs-for-solid-tumours/esmo-mcbs-scorecards/scorecard-163-1. Accessed 12 January 2024

[88]: European Society for Medical Oncology (ESMO) (2023) ESMO-MCBS Scorecards: Avapritinib NAVIGATOR. https://www.esmo.org/guidelines/esmo-mcbs/esmo-mcbs-for-solid-tumours/esmo-mcbs-scorecards/scorecard-211-1. Accessed 12 January 2024

[89]: European Society for Medical Oncology (ESMO) (2023) ESMO-MCBS Scorecards: Entrectinib STARTRK-1, STARTRK-2, ALKA-372-001. https://www.esmo.org/guidelines/esmo-mcbs/esmo-mcbs-for-solid-tumours/esmo-mcbs-scorecards/scorecard-209-1. Accessed 12 January 2024

[90]: European Society for Medical Oncology (ESMO) (2023) ESMO-MCBS Scorecards: Entrectinib STARTRK-1, STARTRK-2, ALKA-372-001. https://www.esmo.org/guidelines/esmo-mcbs/esmo-mcbs-for-solid-tumours/esmo-mcbs-scorecards/scorecard-210-1. Accessed 12 January 2024

[91]: European Society for Medical Oncology (ESMO) (2023) ESMO-MCBS Scorecards: Pemigatinib FIGHT-202. https://www.esmo.org/guidelines/esmo-mcbs/esmo-mcbs-for-solid-tumours/esmo-mcbs-scorecards/scorecard-231-1. Accessed 12 January 2024

[92]: European Society for Medical Oncology (ESMO) (2023) ESMO-MCBS Scorecards: Selpercatinib LIBRETTO-001. https://www.esmo.org/guidelines/esmo-mcbs/esmo-mcbs-for-solid-tumours/esmo-mcbs-scorecards/scorecard-223-1. Accessed 12 January 2024

[93]: European Society for Medical Oncology (ESMO) (2023) ESMO-MCBS Scorecards: Selpercatinib LIBRETTO-001 (Cohort 3). https://www.esmo.org/guidelines/esmo-mcbs/esmo-mcbs-for-solid-tumours/esmo-mcbs-scorecards/scorecard-226-1. Accessed 12 January 2024

[94]: European Society for Medical Oncology (ESMO) (2023) ESMO-MCBS Scorecards: Selpercatinib LIBRETTO-001 (Cohort 1). https://www.esmo.org/guidelines/esmo-mcbs/esmo-mcbs-for-solid-tumours/esmo-mcbs-scorecards/scorecard-218-1. Accessed 12 January 2024

[95]: European Society for Medical Oncology (ESMO) (2023) ESMO-MCBS Scorecards: Selpercatinib LIBRETTO-001. https://www.esmo.org/guidelines/esmo-mcbs/esmo-mcbs-for-solid-tumours/esmo-mcbs-scorecards/scorecard-341-1. Accessed 12 January 2024

[96]: European Society for Medical Oncology (ESMO) (2023) ESMO-MCBS Scorecards: Selpercatinib LIBRETTO-001 (Cohort 4). https://www.esmo.org/guidelines/esmo-mcbs/esmo-mcbs-for-solid-tumours/esmo-mcbs-scorecards/scorecard-227-1. Accessed 12 January 2024

[97]: European Society for Medical Oncology (ESMO) (2023) ESMO-MCBS Scorecards: Tucatinib HER2CLIMB. https://www.esmo.org/guidelines/esmo-mcbs/esmo-mcbs-for-solid-tumours/esmo-mcbs-scorecards/scorecard-167-1. Accessed 12 January 2024

[98]: Arzneimittelkommission der deutschen Ärzteschaft, Fachausschuss der Bundesärztekammer (2017) Anlage III – Vorlage zur Abgabe einer schriftlichen Stellungnahme zur Nutzenbewertung nach § 35a SGB V und Kosten-Nutzen-Bewertung nach § 35b SGB V: Ceritinib (Ablauf Befristung), Nr. 471, A16-62, Version 1.0, Stand: 22. Dezember 2016. https://www.akdae.de/fileadmin/user_upload/akdae/Stellungnahmen/AMNOG/A-Z/Ceritinib/Ceritinib-NB.pdf. Accessed 12 January 2024

[99]: Arzneimittelkommission der deutschen Ärzteschaft, Fachausschuss der Bundesärztekammer (2019) Anlage III – Vorlage zur Abgabe einer schriftlichen Stellungnahme zur Nutzenbewertung nach § 35a SGB V und Kosten-Nutzen-Bewertung nach § 35b SGB V: Trametinib (Melanom), Nr. 697, A18-60, Version 1.0, Stand: 20.12.2018. https://www.akdae.de/fileadmin/user_upload/akdae/Stellungnahmen/AMNOG/A-Z/Trametinib/Trametinib-IE.pdf. Accessed 12 January 2024

[100]: Arzneimittelkommission der deutschen Ärzteschaft, Fachausschuss der Bundesärztekammer (2017) Anlage III – Vorlage zur Abgabe einer schriftlichen Stellungnahme zur Nutzenbewertung nach § 35a SGB V und Kosten-Nutzen-Bewertung nach § 35b SGB V: Osimertinib (nicht-kleinzelliges Lungenkarzinom) [Ablauf Befristung], Nr. 527, A17-20, Version 1.0, Stand: 28.07.2017. https://www.akdae.de/fileadmin/user_upload/akdae/Stellungnahmen/AMNOG/A-Z/Osimertinib/Osimertinib-NB.pdf. Accessed 12 January 2024

[101]: Arzneimittelkommission der deutschen Ärzteschaft, Fachausschuss der Bundesärztekammer (2022) Stellungnahme der Arzneimittelkommission der deutschen Ärzteschaft zur frühen Nutzenbewertung gemäß § 35a SGB V: Palbociclib Neubewertung nach Fristablauf: Mammakarzinom, Patientenpopulation a1. https://www.akdae.de/fileadmin/user_upload/akdae/Stellungnahmen/AMNOG/A-Z/Palbociclib/Palbociclib-221025.pdf. Accessed 12 January 2024

[102]: Arzneimittelkommission der deutschen Ärzteschaft, Fachausschuss der Bundesärztekammer (2018) Anlage III – Vorlage zur Abgabe einer schriftlichen Stellungnahme zur Nutzenbewertung nach § 35a SGB V und Kosten-Nutzen-Bewertung nach § 35b SGB V: Alectinib (nicht-kleinzelliges Lungenkarzinom) (neues Anwendungsgebiet), Nr. 612, A17-67, Version 1.0, Stand: 28.03.2018. https://www.akdae.de/fileadmin/user_upload/akdae/Stellungnahmen/AMNOG/A-Z/Alectinib/Alectinib-EB.pdf. Accessed 12 January 2024

[103]: Arzneimittelkommission der deutschen Ärzteschaft, Fachausschuss der Bundesärztekammer (2019) Anlage III – Vorlage zur Abgabe einer schriftlichen Stellungnahme zur Nutzenbewertung nach § 35a SGB V und Kosten-Nutzen-Bewertung nach § 35b SGB V: Ribociclib (Mammakarzinom), Nr. 752, A19-06, Version 1.0, Stand: 11.04.2019. https://www.akdae.de/fileadmin/user_upload/akdae/Stellungnahmen/AMNOG/A-Z/Ribociclib/Ribociclib-IE.pdf. Accessed 12 January 2024

[104]: Arzneimittelkommission der deutschen Ärzteschaft, Fachausschuss der Bundesärztekammer (2023) Stellungnahme der Arzneimittelkommission der deutschen Ärzteschaft zur frühen Nutzenbewertung gemäß § 35a SGB V: Abemaciclib Neubewertung nach Fristablauf: Mammakarzinom, HR+, HER2-, Kombination mit Aromatasehemmer. https://www.akdae.de/fileadmin/user_upload/akdae/Stellungnahmen/AMNOG/A-Z/Abemaciclib/Abemaciclib-230424.pdf. Accessed 12 January 2024

[105]: Arzneimittelkommission der deutschen Ärzteschaft, Fachausschuss der Bundesärztekammer (2019) Anlage III – Vorlage zur Abgabe einer schriftlichen Stellungnahme zur Nutzenbewertung nach § 35a SGB V und Kosten-Nutzen-Bewertung nach § 35b SGB V: Brigatinib (NSCLC), Nr. 754, A19-08, Version 1.0, Stand: 11.04.2019. https://www.akdae.de/fileadmin/user_upload/akdae/Stellungnahmen/AMNOG/A-Z/Brigatinib/Brigatinib-EB.pdf. Accessed 12 January 2024

[106]: Arzneimittelkommission der deutschen Ärzteschaft, Fachausschuss der Bundesärztekammer (2020) Stellungnahme der Arzneimittelkommission der deutschen Ärzteschaft zur frühen Nutzenbewertung gemäß § 35a SGB V: Larotrectinib solide Tumoren mit einer neurotrophen Tyrosin-Rezeptor-Kinase [NTRK]-Genfusion. https://www.akdae.de/fileadmin/user_upload/akdae/Stellungnahmen/AMNOG/A-Z/Larotrectinib/Larotrectinib-EB.pdf. Accessed 12 January 2024

[107]: Arzneimittelkommission der deutschen Ärzteschaft, Fachausschuss der Bundesärztekammer (2019) Anlage III – Vorlage zur Abgabe einer schriftlichen Stellungnahme zur Nutzenbewertung nach § 35a SGB V und Kosten-Nutzen-Bewertung nach § 35b SGB V: Lorlatinib (NSCLC), Nr. 809, A19-48, Version 1.0, Stand: 29.08.2019. https://www.akdae.de/fileadmin/user_upload/akdae/Stellungnahmen/AMNOG/A-Z/Lorlatinib/Lorlatinib-EB.pdf. Accessed 12 January 2024

[108]: Arzneimittelkommission der deutschen Ärzteschaft, Fachausschuss der Bundesärztekammer (2022) Stellungnahme der Arzneimittelkommission der deutschen Ärzteschaft zur frühen Nutzenbewertung gemäß § 35a SGB V: Lorlatinib Neues Anwendungsgebiet: Nicht-kleinzelliges Lungenkarzinom, ALK+, Erstlinie. https://www.akdae.de/fileadmin/user_upload/akdae/Stellungnahmen/AMNOG/A-Z/Lorlatinib/Lorlatinib-220622.pdf. Accessed 12 January 2024

[109]: Arzneimittelkommission der deutschen Ärzteschaft, Fachausschuss der Bundesärztekammer (2021) Stellungnahme der Arzneimittelkommission der deutschen Ärzteschaft zur frühen Nutzenbewertung gemäß § 35a SGB V: Acalabrutinib chronisch lymphatische Leukämie; Monotherapie, Erstlinie. https://www.akdae.de/fileadmin/user_upload/akdae/Stellungnahmen/AMNOG/A-Z/Acalabrutinib/Acalabrutinib-EB.pdf. Accessed 12 January 2024

[110]: Arzneimittelkommission der deutschen Ärzteschaft, Fachausschuss der Bundesärztekammer (2020) Stellungnahme der Arzneimittelkommission der deutschen Ärzteschaft zur frühen Nutzenbewertung gemäß § 35a SGB V: Entrectinib solide Tumoren mit einer neurotrophen Tyrosin-Rezeptor-Kinase [NTRK]-Genfusion. https://www.akdae.de/fileadmin/user_upload/akdae/Stellungnahmen/AMNOG/A-Z/Entrectinib/Entrectinib-EB.pdf. Accessed 12 January 2024

[111]: Deutsche Gesellschaft für Hämatologie und medizinische Onkologie (2017) Stellungnahme zur Nutzenbewertung gemäß § 35a SGB V: Ceritinib (Neubewertung nach Fristablauf), Vorgangsnummer 2016-10-01-D-259, IQWiG Bericht Nr. 471. https://www.dgho.de/publikationen/stellungnahmen/fruehe-nutzenbewertung/ceritinib/ceritinib-neubewertung-nach-fristablauf-dgho-stellungnahme-20170123.pdf. Accessed 13 January 2024

[112]: Deutsche Gesellschaft für Hämatologie und medizinische Onkologie (2017) Stellungnahme zur Nutzenbewertung des G-BA von Arzneimitteln gemäß § 35a SGB V: Ceritinib, Vorgangsnummer 2017-08-01-D-296, IQWiG Bericht Nr. 552. https://www.dgho.de/publikationen/stellungnahmen/fruehe-nutzenbewertung/ceritinib/ceritinib-dgho-stellungnahme-20171122.pdf. Accessed 13 January 2024

[113]: Deutsche Gesellschaft für Hämatologie und medizinische Onkologie (2016) Stellungnahme zur Nutzenbewertung gemäß § 35a SGB V: Cobimetinib, Vorgangsnummer 2015-12-15-D-196, IQWiG Bericht Nr. 375. https://www.dgho.de/publikationen/stellungnahmen/fruehe-nutzenbewertung/cobimetinib/cobimetinib-dgho-stellungnahme-20160405.pdf. Accessed 14 January 2024

[114]: Deutsche Gesellschaft für Hämatologie und medizinische Onkologie (2015) Stellungnahme zur Nutzenbewertung des IQWiG gemäß § 35a SGB V: Lenvatinib, Vorgangsnummer 2015-07-01-D-164, IQWiG Bericht Nr. 321. https://www.dgho.de/publikationen/stellungnahmen/fruehe-nutzenbewertung/lenvatinib/lenvatinib-dgho-stellungnahme-20151022.pdf. Accessed 13 January 2024

[115]: Deutsche Gesellschaft für Hämatologie und medizinische Onkologie (2017) Stellungnahme zur Nutzenbewertung gemäß § 35a SGB V: Lenvatinib (neues Anwendungsgebiet, Nierenzellkarzinom), Vorgangsnummer 2016-10-01-D-257, IQWiG Bericht Nr. 473. https://www.dgho.de/publikationen/stellungnahmen/fruehe-nutzenbewertung/lenvatinib/lenvatinib-nierenzellkarzinom-dgho-stellungnahme-20170123.pdf. Accessed 13 January 2024

[116]: Deutsche Gesellschaft für Hämatologie und medizinische Onkologie (2019) Stellungnahme zur Nutzenbewertung des G-BA von Arzneimitteln gemäß § 35a SGB V: Lenvatinib (neues Anwendungsgebiet: hepatozelluläres Karzinom), Vorgangsnummer 2018-10-01-D-379, IQWiG Bericht Nr. 694. https://www.dgho.de/publikationen/stellungnahmen/fruehe-nutzenbewertung/lenvatinib/lenvatinib-dgho-stellungnahme-20190123.pdf. Accessed 13 January 2024

[117]: Deutsche Gesellschaft für Hämatologie und medizinische Onkologie (2022) Stellungnahme zur Nutzenbewertung des G-BA von Arzneimitteln gemäß § 35a SGB V: Lenvatinib in Kombination mit Pembrolizumab (Endometriumkarzinom), Vorgangsnummer 2021-12-15-D-755, IQWiG Bericht Nr. 1331. https://www.dgho.de/publikationen/stellungnahmen/fruehe-nutzenbewertung/lenvatinib/lenvatinib-pembrolizumab-endometriumkarzinom-20220510.pdf. Accessed 02 February 2024

[118]: Deutsche Gesellschaft für Hämatologie und medizinische Onkologie (2022) Stellungnahme zur Nutzenbewertung gemäß § 35a SGB V: Lenvatinib in Kombination mit Pembrolizumab (Nierenzellkarzinom), Vorgangsnummer 2021-12-15-D-749, IQWiG Bericht Nr. 1327. https://www.dgho.de/publikationen/stellungnahmen/fruehe-nutzenbewertung/lenvatinib/lenvatinib-pembrolizumab-dgho-dgu-stellungnahme-20220510.pdf. Accessed 02 February 2024

[119]: Deutsche Gesellschaft für Hämatologie und medizinische Onkologie (2015) Stellungnahme zur Nutzenbewertung gemäß § 35a SGB V: Nintedanib, Vorgangsnummer 2015-01-01-D-147, IQWiG Bericht Nr. 290. https://www.dgho.de/publikationen/stellungnahmen/fruehe-nutzenbewertung/nintedanib/nintetanib-dgho-stellungnahme-20150422.pdf. Accessed 13 January 2024

[120]: Deutsche Gesellschaft für Hämatologie und medizinische Onkologie (2016) Stellungnahme zur Nutzenbewertung gemäß § 35a SGB V: Trametinib, Vorgangsnummer 2015-10-01-D-183, IQWiG Bericht Nr. 354. https://www.dgho.de/publikationen/stellungnahmen/fruehe-nutzenbewertung/trametinib/trametinib-dgho-stellungnahme-20160125.pdf. Accessed 13 January 2024

[121]: Deutsche Gesellschaft für Hämatologie und medizinische Onkologie (2019) Stellungnahme zur Nutzenbewertung des G-BA von Arzneimitteln gemäß § 35a SGB V: Trametinib, in Kombination mit Dabrafenib (Melanom: neues Anwendungsgebiet, adjuvante Therapie), Vorgangsnummer 2018-10-01-D-384, IQWiG Bericht Nr. 697. https://www.dgho.de/publikationen/stellungnahmen/fruehe-nutzenbewertung/trametinib/trametinib-melanom-adjuvante-therapie-dgho-stellungnahme-20190123.pdf. Accessed 13 January 2024

[122]: Deutsche Gesellschaft für Hämatologie und medizinische Onkologie (2017) Stellungnahme zur Nutzenbewertung des G-BA von Arzneimitteln gemäß § 35a SGB V: Trametinib, Vorgangsnummer 2017-05-01-D-284, IQWiG Bericht Nr. 523. https://www.dgho.de/publikationen/stellungnahmen/fruehe-nutzenbewertung/trametinib/trametinib-nsclc-dgho-stellungnahme-20170822.pdf. Accessed 02 February 2024

[123]: Deutsche Gesellschaft für Hämatologie und medizinische Onkologie (2017) Stellungnahme zur Nutzenbewertung des G-BA von Arzneimitteln gemäß § 35a SGB V: Osimertinib (Neubewertung nach Fristablauf), Vorgangsnummer 2017-05-01-D-282, IQWiG Bericht Nr. 527. https://www.dgho.de/publikationen/stellungnahmen/fruehe-nutzenbewertung/osimertinib/osimertinib-neubewertung-dgho-stellungnahme-20170822.pdf. Accessed 13 January 2024

[124]: Deutsche Gesellschaft für Hämatologie und medizinische Onkologie (2018) Stellungnahme zur Nutzenbewertung des G-BA von Arzneimitteln gemäß § 35a SGB V: Osimertinib (neues Anwendungsgebiet, Erstlinie), Vorgangsnummer 2018-07-15-D-369, IQWiG Bericht Nr. 674. https://www.dgho.de/publikationen/stellungnahmen/fruehe-nutzenbewertung/osimertinib/osimertinib-erstlinie-dgho-stellungnahme-20181105.pdf. Accessed 13 January 2024

[125]: Deutsche Gesellschaft für Hämatologie und medizinische Onkologie (2021) Stellungnahme zur Nutzenbewertung des G-BA von Arzneimitteln gemäß § 35a SGB V: Osimertinib (neues Anwendungsgebiet, EGFR-Mutationen, adjuvant), Vorgangsnummer 2018-07-01-D-701, IQWiG Bericht Nr. 1207. https://www.dgho.de/publikationen/stellungnahmen/fruehe-nutzenbewertung/osimertinib/osimertinib-adjuvant-dgho-aio-dgp-stellungnahme-20211022.pdf. Accessed 02 February 2024

[126]: Deutsche Gesellschaft für Hämatologie und medizinische Onkologie (2019) Stellungnahme zur Nutzenbewertung des G-BA von Arzneimitteln gemäß § 35a SGB V: Palbociclib (Neubewertung nach Fristablauf), Vorgangsnummer 2018-10-01-D-395, IQWiG Bericht Nr. 701. https://www.dgho.de/publikationen/stellungnahmen/fruehe-nutzenbewertung/palbociclib/palbociclib-dgho-stellungnahme-20190123.pdf. Accessed 14 January 2024

[127]: Deutsche Gesellschaft für Hämatologie und medizinische Onkologie (2017) Stellungnahme zur Nutzenbewertung des G-BA von Arzneimitteln gemäß § 35a SGB V: Alectinib, Vorgangsnummer 2017-05-01-D-281, IQWiG Bericht Nr. 526. https://www.dgho.de/publikationen/stellungnahmen/fruehe-nutzenbewertung/alectinib/alectinib-dgho-stellungnahme-20170822.pdf. Accessed 14 January 2024

[128]: Deutsche Gesellschaft für Hämatologie und medizinische Onkologie (2018) Stellungnahme zur Nutzenbewertung des G-BA von Arzneimitteln gemäß § 35a SGB V: Alectinib (neues Anwendungsgebiet, Erstlinie), Vorgangsnummer 2018-01-01-D-326, IQWiG Bericht Nr. 612. https://www.dgho.de/publikationen/stellungnahmen/fruehe-nutzenbewertung/alectinib/alectinib-erstlinie-dgho-stellungnahme-20180424.pdf. Accessed 14 January 2024

[129]: Deutsche Gesellschaft für Hämatologie und medizinische Onkologie (2018) Stellungnahme zur Nutzenbewertung des G-BA von Arzneimitteln für seltene Erkrankungen gemäß § 35a SGB V: Midostaurin A. Akute Myeloische Leukämie, B. Fortgeschrittene systemische Mastozytose, Vorgangsnummer 2017-10-15-D-319, IQWiG Bericht Nr. 580. https://www.dgho.de/publikationen/stellungnahmen/fruehe-nutzenbewertung/midostaurin/midostaurin-dgho-stellungnahme-20180205.pdf. Accessed 14 January 2024

[130]: Deutsche Gesellschaft für Hämatologie und medizinische Onkologie (2020) Stellungnahme zur Nutzenbewertung des G-BA von Arzneimitteln gemäß § 35a SGB V: Ribociclib (Kombination mit Aromatasehemmer, Neubewertung nach Fristablauf), Vorgangsnummer 2020-03-01-D-518, IQWiG Bericht Nr. 918. https://www.dgho.de/publikationen/stellungnahmen/fruehe-nutzenbewertung/ribociclib/ribociclib-ai-dgho-stellungnahme-20200623.pdf. Accessed 14 January 2024

[131]: Deutsche Gesellschaft für Hämatologie und medizinische Onkologie (2019) Stellungnahme zur Nutzenbewertung des G-BA von Arzneimitteln gemäß § 35a SGB V: Ribociclib (neues Anwendungsgebiet, Kombination mit Fulvestrant, Behandlung prä-/perimenopausaler Patientinnen), Vorgangsnummer 2019-01-15-D-430, IQWiG Bericht Nr. 752. https://www.dgho.de/publikationen/stellungnahmen/fruehe-nutzenbewertung/ribociclib/ribociclib-dgho-stellungnahme-20190506.pdf. Accessed 14 January 2024

[132]: Deutsche Gesellschaft für Hämatologie und medizinische Onkologie (2018) Stellungnahme zur Nutzenbewertung gemäß § 35a SGB V: Tivozanib (Nierenzellkarzinom, Neubewertung nach Fristablauf), Vorgangsnummer 2017-11-01-D-3323, IQWiG Bericht Nr. 591. https://www.dgho.de/publikationen/stellungnahmen/fruehe-nutzenbewertung/tivozanib/tivozanib-nierenzellkarzinom-dgho-dgu-stellungnahme-20180222.pdf. Accessed 14 January 2024

[133]: Deutsche Gesellschaft für Hämatologie und medizinische Onkologie (2023) Stellungnahme zur Nutzenbewertung des G-BA von Arzneimitteln gemäß § 35a SGB V: Abemaciclib (in Kombination mit einem Aromatasehemmer), Vorgangsnummer 2023-01-01-D-899, IQWiG Bericht Nr. 1530. https://www.dgho.de/publikationen/stellungnahmen/fruehe-nutzenbewertung/abemaciclib/abemaciclib-aromatasehemmer-dgho-stellungnahme-20230424.pdf. Accessed 02 February 2024

[134]: Deutsche Gesellschaft für Hämatologie und medizinische Onkologie (2022) Stellungnahme zur Nutzenbewertung gemäß § 35a SGB V: Abemaciclib (Kombination mit Fulvestrant, Neubewertung nach Fristablauf), Vorgangsnummer 2021-12-01-D-754, IQWiG Bericht Nr. 1304. https://www.dgho.de/publikationen/stellungnahmen/fruehe-nutzenbewertung/abemaciclib/abemaciclib-dgho-stellungnahme-20220322.pdf. Accessed 02 February 2024

[135]: Deutsche Gesellschaft für Hämatologie und medizinische Onkologie (2022) Stellungnahme zur Nutzenbewertung gemäß § 35a SGB V: Abemaciclib (neues Anwendungsgebiet: adjuvante endokrine Kombinationstherapie beim HR+/HER2- Mammakarzinom und hohem Rezidivrisiko), Vorgangsnummer 2022-05-01-D-811, IQWiG Bericht Nr. 1394. https://www.dgho.de/publikationen/stellungnahmen/fruehe-nutzenbewertung/abemaciclib/abemaciclib-dgho-stellungnahme-20220822.pdf. Accessed 02 February 2024

[136]: Deutsche Gesellschaft für Hämatologie und medizinische Onkologie (2019) Stellungnahme zur Nutzenbewertung des G-BA von Arzneimitteln gemäß § 35a SGB V: Binimetinib, in Kombination mit Encorafenib, Vorgangsnummer 2018-10-01-D-388, IQWiG Bericht Nr. 708. https://www.dgho.de/publikationen/stellungnahmen/fruehe-nutzenbewertung/binimetinib/binimetinib-dgho-stellungnahme-20190123.pdf. Accessed 02 February 2024

[137]: Deutsche Gesellschaft für Hämatologie und medizinische Onkologie (2019) Stellungnahme zur Nutzenbewertung des G-BA von Arzneimitteln gemäß § 35a SGB V: Encorafenib, in Kombination mit Binimetinib, Vorgangsnummer 2018-10-01-D-389, IQWiG Bericht Nr. 707. https://www.dgho.de/publikationen/stellungnahmen/fruehe-nutzenbewertung/encorafenib/encorafenib-dgho-stellungnahme-20190123.pdf. Accessed 02 February 2024

[138]: Deutsche Gesellschaft für Hämatologie und medizinische Onkologie (2020) Stellungnahme zur Nutzenbewertung gemäß § 35a SGB V: Encorafenib (Neues Anwendungsgebiet, metastasiertes kolorektales Karzinom), Vorgangsnummer 2020-07-01-D-551, IQWiG Bericht Nr. 976. https://www.dgho.de/publikationen/stellungnahmen/fruehe-nutzenbewertung/encorafenib/encorafenib-neues-anwendungsgebiet-kolorektales-karzinom-stellungnahme-20201022.pdf. Accessed 02 February 2024

[139]: Deutsche Gesellschaft für Hämatologie und medizinische Onkologie (2019) Stellungnahme zur Nutzenbewertung des G-BA von Arzneimitteln gemäß § 35a SGB V: Brigatinib, Vorgangsnummer 2019-01-15-D-434, IQWiG Bericht Nr. 754. https://www.dgho.de/publikationen/stellungnahmen/fruehe-nutzenbewertung/brigatinib/brigatinib-zweitlinie-dgho-aio-stellungnahme-20190506.pdf. Accessed 02 February 2024

[140]: Deutsche Gesellschaft für Hämatologie und medizinische Onkologie (2020) Stellungnahme zur Nutzenbewertung des G-BA von Arzneimitteln gemäß § 35a SGB V: Brigatinib (neues Anwendungsgebiet, Erstlinie), Vorgangsnummer 2020-05-01-D-542, IQWiG Bericht Nr. 950. https://www.dgho.de/publikationen/stellungnahmen/fruehe-nutzenbewertung/brigatinib/brigatinib-erstlinie-dgho-aio-dgp-stellungnahme-20200824.pdf. Accessed 02 February 2024

[141]: Deutsche Gesellschaft für Hämatologie und medizinische Onkologie (2019) Stellungnahme zur Nutzenbewertung des G-BA von Arzneimitteln gemäß § 35a SGB V: Dacomitinib, Vorgangsnummer 2019-05-01-D-442, IQWiG Bericht Nr. 800. https://www.dgho.de/publikationen/stellungnahmen/fruehe-nutzenbewertung/dacomitinib/dacomitinib-dgho-aio-stellungnahme-20190822.pdf. Accessed 02 February 2024

[142]: Deutsche Gesellschaft für Hämatologie und medizinische Onkologie (2020) Stellungnahme zur Nutzenbewertung des G-BA von Arzneimitteln für seltene Erkrankungen gemäß § 35a SGB V: Gilteritinib, Vorgangsnummer 2018-12-01-D-503, IQWiG Bericht Nr. 884. https://www.dgho.de/publikationen/stellungnahmen/fruehe-nutzenbewertung/gilteritinib/gilterinib-dgho-stellungnahme-20200323.pdf. Accessed 03 February 2024

[143]: Deutsche Gesellschaft für Hämatologie und medizinische Onkologie (2020) Stellungnahme zur Nutzenbewertung gemäß § 35a SGB V: Larotrectinib, Vorgangsnummer 2019-10-15-D-495, IQWiG Bericht Nr. 866. https://www.dgho.de/publikationen/stellungnahmen/fruehe-nutzenbewertung/larotrectinib/larotrectinib-dgho-dgp-gpoh-stellungnahme-20200205.pdf. Accessed 03 February 2024

[144]: Deutsche Gesellschaft für Hämatologie und medizinische Onkologie (2019) Stellungnahme zur Nutzenbewertung des G-BA von Arzneimitteln gemäß § 35a SGB V: Lorlatinib, Vorgangsnummer 2019-06-01-D-451, IQWiG Bericht Nr. 809. https://www.dgho.de/publikationen/stellungnahmen/fruehe-nutzenbewertung/lorlatinib/lorlatinib-dgho-aio-stellungnahme-20190923.pdf. Accessed 03 February 2024

[145]: Deutsche Gesellschaft für Hämatologie und medizinische Onkologie (2022) Stellungnahme zur Nutzenbewertung des G-BA von Arzneimitteln gemäß § 35a SGB V: Lorlatinib (neues Anwendungsgebiet, Erstlinie), Vorgangsnummer 2022-02-01-D-792, IQWiG Bericht Nr. 1365. https://www.dgho.de/publikationen/stellungnahmen/fruehe-nutzenbewertung/lorlatinib/lorlatinib-erstlinie-dgho-aio-dgp-stellungnahme-20220622.pdf. Accessed 03 February 2024

[146]: Deutsche Gesellschaft für Hämatologie und medizinische Onkologie (2020) Stellungnahme zur Nutzenbewertung des G-BA von Arzneimitteln gemäß § 35a SGB V: Neratinib, Vorgangsnummer 2019-12-01-D-506, IQWiG Bericht Nr. 886. https://www.dgho.de/publikationen/stellungnahmen/fruehe-nutzenbewertung/neratinib/neratinib-dgho-stellungnahme-20200323.pdf. Accessed 03 February 2024

[147]: Deutsche Gesellschaft für Hämatologie und medizinische Onkologie (2021) Stellungnahme zur Nutzenbewertung gemäß § 35a SGB V: Acalabrutinib Monotherapie (Erstlinie), Vorgangsnummer 2020-12-01-D-592, IQWiG Bericht Nr. 1076. https://www.dgho.de/publikationen/stellungnahmen/fruehe-nutzenbewertung/acalabrutinib/acalabrutinib-mono-cll-erstlinie-stellungnahme-20210406.pdf. Accessed 03 February 2024

[148]: Deutsche Gesellschaft für Hämatologie und medizinische Onkologie (2021) Stellungnahme zur Nutzenbewertung gemäß § 35a SGB V: Acalabrutinib in Kombination mit Obinutuzumab (Erstlinie), Vorgangsnummer 2020-12-01-D-593, IQWiG Bericht Nr. 1077. https://www.dgho.de/publikationen/stellungnahmen/fruehe-nutzenbewertung/acalabrutinib/acalabrutinib-obinutuzumab-cll-erstlinie-stellungnahme-20210406.pdf. Accessed 03 February 2024

[149]: Deutsche Gesellschaft für Hämatologie und medizinische Onkologie (2021) Stellungnahme zur Nutzenbewertung gemäß § 35a SGB V: Acalabrutinib (Zweitlinie), Vorgangsnummer 2020-12-01-D-594, IQWiG Bericht Nr. 1071. https://www.dgho.de/publikationen/stellungnahmen/fruehe-nutzenbewertung/acalabrutinib/acalabrutinib-cll-zweitlinie-stellungnahme-20210406.pdf. Accessed 03 February 2024

[150]: Deutsche Gesellschaft für Hämatologie und medizinische Onkologie (2020) Stellungnahme zur Nutzenbewertung des G-BA von Arzneimitteln gemäß § 35a SGB V: Alpelisib (Kombination mit Fulvestrant), Vorgangsnummer 2020-09-01-D-574, IQWiG Bericht Nr. 1002. https://www.dgho.de/publikationen/stellungnahmen/fruehe-nutzenbewertung/alpelisib/alpelisib-dgho-stellungnahme-20201222.pdf. Accessed 03 February 2024

[151]: Deutsche Gesellschaft für Hämatologie und medizinische Onkologie (2021) Stellungnahme zur Nutzenbewertung des G-BA von Arzneimitteln für seltene Leiden gemäß § 35a SGB V: Avapritinib, Vorgangsnummer 2020-11-01-D-583, IQWiG Bericht Nr. 1027. https://www.dgho.de/publikationen/stellungnahmen/fruehe-nutzenbewertung/avapritinib/avapritinib-aio-dgho-dgvs-stellungnahme-20210217.pdf. Accessed 03 February 2024

[152]: Deutsche Gesellschaft für Hämatologie und medizinische Onkologie (2022) Stellungnahme zur Nutzenbewertung des G-BA von Arzneimitteln für seltene Leiden gemäß § 35a SGB V: Avapritinib (neues Anwendungsgebiet: fortgeschrittene systemische Mastozytose), Vorgangsnummer 2022-04-01-D-798, IQWiG Bericht Nr. 1372. https://www.dgho.de/publikationen/stellungnahmen/fruehe-nutzenbewertung/avapritinib/avapritinib-stellungnahme-20220722.pdf. Accessed 03 February 2024

[153]: Deutsche Gesellschaft für Hämatologie und medizinische Onkologie (2020) Stellungnahme zur Nutzenbewertung gemäß § 35a SGB V: Entrectinib bei soliden Tumoren mit NTRK-Genfusion, Vorgangsnummer 2020-09-01-D-559, IQWiG Bericht Nr. 1007. https://www.dgho.de/publikationen/stellungnahmen/fruehe-nutzenbewertung/entrectinib/entrectinib-ntrk-dgho-gpoh-stellungnahme-20201222.pdf. Accessed 03 February 2024

[154]: Deutsche Gesellschaft für Hämatologie und medizinische Onkologie (2020) Stellungnahme zur Nutzenbewertung gemäß § 35a SGB V: Entrectinib beim ROS1-positiven nicht-kleinzelligen Lungenkarzinom (NSCLC), Vorgangsnummer 2020-09-01-D-558, IQWiG Bericht Nr. 1003. https://www.dgho.de/publikationen/stellungnahmen/fruehe-nutzenbewertung/entrectinib/entrectinib-ros1-dgho-dgp-aio-stellungnahme-20201222.pdf. Accessed 03 February 2024

[155]: Deutsche Gesellschaft für Hämatologie und medizinische Onkologie (2021) Stellungnahme zur Nutzenbewertung des G-BA von Arzneimitteln für seltene Leiden gemäß § 35a SGB V: Fedratinib, Vorgangsnummer 2021-03-15-D-650, IQWiG Bericht Nr. 1135. https://www.dgho.de/publikationen/stellungnahmen/fruehe-nutzenbewertung/fedratinib/fedratinib-dgho-stellungnahme-20210706.pdf. Accessed 03 February 2024

[156]: Deutsche Gesellschaft für Hämatologie und medizinische Onkologie (2021) Stellungnahme zur Nutzenbewertung des G-BA von Arzneimitteln für seltene Leiden gemäß § 35a SGB V: Pemigatinib, Vorgangsnummer 2021-04-15-D-670, IQWiG Bericht Nr. 1157. https://www.dgho.de/publikationen/stellungnahmen/fruehe-nutzenbewertung/pemigatinib/pemigatinib-stellungnahme-20210805.pdf. Accessed 03 February 2024

[157]: Deutsche Gesellschaft für Hämatologie und medizinische Onkologie (2021) Stellungnahme zur Nutzenbewertung gemäß § 35a SGB V: Selpercatinib beim RET-fusionspositiven nicht-kleinzelligen Lungenkarzinom (NSCLC), Vorgangsnummer 2021-03-15-D-664, IQWiG Bericht Nr. 1130. https://www.dgho.de/publikationen/stellungnahmen/fruehe-nutzenbewertung/selpercatinib/selpercatinib-nsclc-dgho-dgp-aio-stellungnahme-20210706.pdf. Accessed 03 February 2024

[158]: Deutsche Gesellschaft für Hämatologie und medizinische Onkologie (2021) Stellungnahme zur Nutzenbewertung gemäß § 35a SGB V: Selpercatinib beim RET-mutierten, medullären Schilddrüsenkarzinom, Vorgangsnummer 2021-03-15-D-656, IQWiG Bericht Nr. 1132. https://www.dgho.de/publikationen/stellungnahmen/fruehe-nutzenbewertung/selpercatinib/selpercatinib-sd-medullaer-stellungnahme-20210706.pdf. Accessed 03 February 2024

[159]: Deutsche Gesellschaft für Hämatologie und medizinische Onkologie (2021) Stellungnahme zur Nutzenbewertung gemäß § 35a SGB V: Selpercatinib beim RET-fusionspositiven Schilddrüsenkarzinom, Vorgangsnummer 2021-03-15-D-657, IQWiG Bericht Nr. 1133. https://www.dgho.de/publikationen/stellungnahmen/fruehe-nutzenbewertung/selpercatinib/selpercatinib-sd-dgho-stellungnahme-20210706.pdf. Accessed 03 February 2024

[160]: Deutsche Gesellschaft für Hämatologie und medizinische Onkologie (2022) Stellungnahme zur Nutzenbewertung gemäß § 35a SGB V: Selpercatinib beim RET-fusionspositiven nicht-kleinzelligen Lungenkarzinom (NSCLC), Vorgangsnummer 2022-07-01-D-832, IQWiG Bericht Nr. 1427. https://www.dgho.de/publikationen/stellungnahmen/fruehe-nutzenbewertung/selpercatinib/selpercatinib-dgho-nsclc-stellungnahme-20221025.pdf. Accessed 03 February 2024

[161]: Deutsche Gesellschaft für Hämatologie und medizinische Onkologie (2023) Gemeinsame Stellungnahme zur Nutzenbewertung des G-BA von Arzneimitteln gemäß § 35a SGB V: Selpercatinib, Vorgangsnummer 2022-10-01-D-874, IQWiG Bericht Nr. 1479. https://www.dgho.de/publikationen/stellungnahmen/fruehe-nutzenbewertung/selpercatinib/selpercatinib-dgho-stellungnahme-20230123.pdf. Accessed 03 February 2024

[162]: Deutsche Gesellschaft für Hämatologie und medizinische Onkologie (2021) Stellungnahme zur Nutzenbewertung des G-BA von Arzneimitteln gemäß § 35a SGB V: Tucatinib (in Kombination mit Trastuzumab + Capecitabin), Vorgangsnummer 2021-03-15-D-654, IQWiG Bericht Nr. 1129. https://www.dgho.de/publikationen/stellungnahmen/fruehe-nutzenbewertung/tucatinib/tucatinib-dgho-degro-stellungnahme-20210706.pdf. Accessed 03 February 2024

**Reference Table S 4:** IUPHAR/BPS Guide to Pharmacology: https://www.guidetopharmacology.org. Accessed 08 March 2024
